# Semiartificial CO_2_ Fixation Using Metal-Dependent Formate Dehydrogenase

**DOI:** 10.1021/acs.chemrev.6c00095

**Published:** 2026-06-24

**Authors:** Yongpeng Liu, Beverly Q. L. Low, William E. Robinson, Rita R. Manuel, Ana Rita Oliveira, Inês A. C. Pereira, Erwin Reisner

**Affiliations:** † Yusuf Hamied Department of Chemistry, 2152 University of Cambridge, Cambridge CB2 1EW, U.K.; ‡ Institute for Molecules and Materials, Radboud University Nijmegen, Heyendaalseweg 135, 6525 AJ Nijmegen, The Netherlands; § Instituto de Tecnologia Química e Biológica António Xavier (ITQB NOVA), 98819 Universidade NOVA de Lisboa, Av. da República, 2780-157 Oeiras, Portugal

## Abstract

Semiartificial photosynthesis exploits synergies between synthetic light absorbers and biological catalysts, offering a promising strategy for solar chemistry. Unlike conventional synthetic catalysts for carbon dioxide (CO_2_) fixation, biological systems employ enzymes, most notably formate dehydrogenases (Fdhs), to catalyze the interconversion between CO_2_, protons and electrons into formate, a central hub molecule in energy and carbon metabolism. This review focuses on the deployment of metal-dependent Fdhs in semiartificial photosynthesis, with an emphasis on molybdenum- and tungsten-dependent enzymes directly wired to electrodes and synthetic light absorbers. We first examine the structural, mechanistic, and redox properties of relevant Fdhs *in vivo* and *in vitro*, highlighting reaction pathways and inherent challenges. Subsequent sections discuss the central role of biotic–abiotic interfaces in constructing functional biohybrid systems, highlighting how advanced interfacial characterization techniques inform enzyme loading, charge carrier dynamics, and reaction intermediates. We then summarize progress and challenges in (photo)­electrochemical and photochemical systems leveraging Fdhs unique properties as a model catalyst for CO_2_-to-formate conversion. This review aims to clarify the current state of the semiartificial photosynthesis field employing metal-dependent Fdhs *in vitro*, and guide future research at the interface of enzymology, photo­(electro)­chemistry, and materials science.

## Graphical abstract

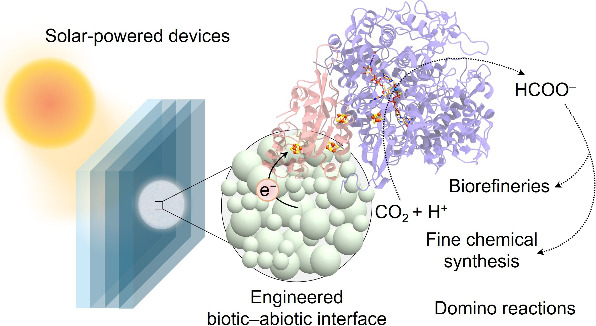

## 1 Introduction

The anthropogenic greenhouse-gas emissions resulting from fossil fuel combustion are raising the atmospheric carbon dioxide (CO_2_) concentration to increasingly high levels, presenting one of the most pressing global challenges of our time.^1–6^ While mitigation of CO_2_ emissions remains important,^7–11^ the valorization of CO_2_ to value-added chemicals and fuels through catalytic processes offers a complementary route toward a circular chemical industry.^12–16^ This approach repositions CO_2_ from a waste into a valuable carbon feedstock, reducing atmospheric greenhouse levels while providing sustainable alternatives to petrochemical building blocks. In particular, the two-electron CO_2_ reduction product, formate, is a versatile hub molecule, being stable in aqueous solution, readily separable, and usable as a carbon and energy source, a liquid-hydrogen carrier, a metabolite, or an intermediate for downstream chemistry.^17–19^

However, the valorization of CO_2_ is hindered by its thermodynamic stability and sluggish kinetics, as synthetic electrocatalysts typically require high overpotentials and often exhibit poor selectivity for formate, thereby wasting energy and producing mixed gaseous and liquid products.^20–24^ These inherent limitations of artificial approaches motivate the exploration of alternative catalytic platforms capable of selective and efficient operation under ambient conditions.

Natural CO_2_ fixation, including geochemical processes, inorganic pathways, and photosynthesis, is essential for nearly all forms of life.^25–28^ A notable feature of natural photosynthesis is the use of enzymes, such as ribulose-1,5-bisphosphate carboxylase/oxygenase (RuBisCO, the most studied carboxylase),^29–31^ carbon monoxide dehydrogenase,^32–34^ and formate dehydrogenase (Fdh),^35–37^ which catalyze reactions with high efficiency, defined as the rapid conversion of substrates to products with minimal energy input, and with high selectivity under ambient conditions. Understanding how nature achieves these transformations provides inspiration for designing semiartificial or fully artificial systems that combine the robustness and scalability of synthetic materials with the precision and efficiency of enzymatic catalysis.^38–41^ Thus, biological carbon fixation not only sustains ecosystems but also provides a blueprint for sustainable technologies to recycle CO_2_.

Fdhs are a family of enzymes that catalyze the reversible interconversion between CO_2_, protons (H^+^), electrons (e^–^), and formate (HCOO^–^) at neutral pH (Figure 1a, eq 1).^35,42–44^

1 $${\mathrm{CO}}_{2}+H^{+}+2e^{-}↔{\mathrm{HCOO}}^{-}$$

$$E=E^{{}^{\circ}\prime }-\frac{RT}{2F}\times \ln\left(\frac{1+K_{\mathrm{ox}1}/\left[H^{+}\right]\left(1+K_{\mathrm{ox}2}/\left[H^{+}\right]\right)}{1+K_{\mathrm{red}1}/\left[H^{+}\right]}\times \frac{1}{{\left[H^{+}\right]}^{2}}\right)$$ 2

**Figure 1:**
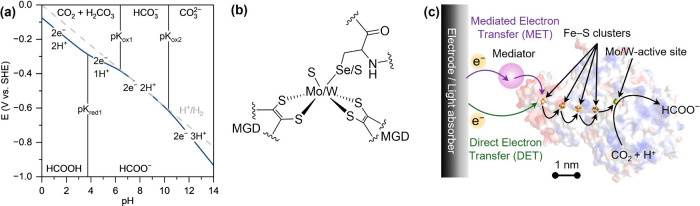
(a) Pourbaix diagram for the interconversion among formic acid, formate, CO_2_, carbonate, bicarbonate, and carbonic acid at 25 °C. The pH-dependent reduction potentials (blue solid line) were calculated using the Nernst equation (eq 2) using the following parameters: p*K*_red1_ = 3.75, p*K*_ox1_ = 6.39, p*K*_ox2_ = 10.32, and formal potential (*E*°) of −0.075 V.^45–49^ (b) A simplified general representation of the Mo or W active site in a metal-dependent Fdh. (c) Schematic of mediated and direct electron transfer pathways from an electrode or a light absorber to the Mo or W active site through four Fe–S clusters for CO_2_ reduction to formate.

Metal-dependent Fdhs (see Section 2.2 for classification) contain a molybdenum (Mo) or tungsten (W) active site that is hexacoordinated by four sulfur atoms from two molybdopterin guanine dinucleotide (MGD) pyranopterins, one terminal sulfur ligand, and either a cysteine (Cys) or selenocysteine (Sec) to complete the primary coordination sphere (Figure 1b).^50–54^ This metal active site underpins their unique catalytic properties (see Section 3 for details), including electrocatalytic reversibility, which is the ability to operate at the thermodynamic potential and thus drive both forward and backward reactions at a marginal overpotential under mild conditions (see Section 5.1 for electrochemistry). This unique molecular architecture also enables Fdhs to operate at a level of efficiency and rate of catalysis (turnover frequency, TOF) that has not yet been matched by synthetic catalysts.^55^

In a metal-dependent Fdh, multiple iron–sulfur (Fe–S) clusters connect the deeply buried active site to the enzyme surface (Figure 1c), providing a natural ‘wire’ that enables efficient electron transfer pathways to physiological redox partners (see Section 2.3 for details).^36,56–58^ Using the CO_2_ reduction reaction as an illustration, electrons are either transferred directly through physical contact at the interface (direct electron transfer, DET)^59^ or shuttled via a small molecular redox-active mediator (mediated electron transfer, MET)^60^ from a source (e.g., an electrode or light absorber) to the distal Fe–S cluster of Fdh (Figure 1c). Subsequently, the electrons are relayed through the Fe–S clusters to the Mo or W active site for CO_2_ reduction to formate. This structural feature enables certain metal-dependent Fdhs to be directly interfaced with electrodes and light absorbers to achieve DET in a semiartificial assembly.^49,61^ In contrast to metal-independent Fdhs (see Section 2.1 for classification), metal-dependent Fdhs display substantially higher catalytic activities, particularly for CO_2_ reduction. Despite these advantages, several challenges persist, including oxygen sensitivity, difficulties and high cost in expression and purification, higher catalytic turnover rates for formate oxidation than for CO_2_ reduction, inhibition of cofactors, and the large carbon footprint in producing enzymes^62–64^ compared to synthetic catalysts.

The concept of semiartificial photosynthesis, which integrates the selectivity of enzymes with synthetic light absorbers, has emerged as a promising strategy for solar energy conversion and storage.^65–68^ By bypassing natural redox partners (see Section 2.3 for details), this approach enables renewable electrons to be directly delivered to biological catalysts, resulting in highly efficient and selective catalytic transformations. Semiartificial systems have been successfully demonstrated with a range of enzymes, including hydrogenases for proton reduction to hydrogen,^69–74^ nitrogenases for dinitrogen (N_2_) reduction to ammonia (NH_3_),^75–77^ carbon monoxide dehydrogenases for CO_2_ reduction to carbon monoxide (CO),^78–81^ as well as Photosystem I for light harvesting^82–86^ and Photosystem II for water oxidation to molecular oxygen (O_2_).^87–94^ The implementation of Fdh in semiartificial photosynthesis offers a promising route for solar-driven CO_2_ reduction to formate and constitutes the focus of this review. Complementary reviews covering semiartificial photosynthesis,^65–68,95–97^ as well as the use of Fdh in catalytic CO_2_ fixation,^35–37,43,98–102^ are available elsewhere.

In this review, we provide a comprehensive overview and critical perspective on metal-dependent Fdhs and their applications in semiartificial CO_2_ fixation with a focus on establishing DET. We first examine the mechanism, structures, and purification of the major classes of Fdhs and their variants, with particular attention to their behavior *in vivo* and *in vitro*. We then discuss the central role of interfacial engineering at the biotic–abiotic interfaces for constructing functional biohybrid systems, highlighting how advanced interfacial characterization techniques can provide essential insights into enzyme loading, desorption, reaction intermediates, and charge transfer and recombination processes. This is followed by a summary of the historical development and recent progress in photo­(electro)­catalytic systems for semiartificial CO_2_ reduction, with emphasis on establishing DET to Fdhs in electrochemical, photoelectrochemical, and photocatalytic applications. Finally, we identify knowledge gaps, outline future directions, and evaluate the prospects of semiartificial CO_2_ fixation with metal-dependent Fdhs as a sustainable route for CO_2_ valorization beyond formate.

## 2 Fdh *in* *Vivo*

This section focuses on the roles and applications of Fdhs *in vivo*, providing an overview of the known types of Fdh. It introduces the classification of Fdhs, with particular emphasis on metal-dependent enzymes, especially W-dependent Fdhs that have been widely used in semiartificial photosynthesis, and discusses their structural features and evolutionary origins. The section also examines the metabolic pathways in which metal-dependent Fdhs operate *in vivo*, highlighting their catalytic chemistry and ability to catalyze CO_2_ reduction and formate oxidation reversibly. Finally, the distribution of these enzymes across diverse organisms is outlined.

### 2.1 Metal-Independent Fdhs

Fdhs are found across a wide range of organisms and can be divided into two main groups. The first group, metal-independent Fdhs, occurs in diverse organisms including aerobic bacteria, yeasts, and plants.^103^ These enzymes are O_2_ tolerant and belong to the D-specific dehydrogenases of the 2-oxyacid family (also known as d-isomer specific 2-hydroxyacid dehydrogenases).^104–107^ Structurally, metal-independent Fdhs are composed of two subunits containing two independent active sites and do not require a metal center or a prosthetic group (i.e., a nonamino acid molecule, either organic or inorganic, that binds tightly to a protein and is required for its biological function).^103^ The primary *in vivo* role of metal-independent Fdhs is the oxidation of formate to CO_2_ coupled to the regeneration of the nicotinamide adenine dinucleotide (NAD) cofactor by reducing its oxidized form, although some metal-independent Fdhs can also catalyze CO_2_ reduction to formate under specific conditions (see Section 2.3 for metabolic pathways). The NAD cofactor consists of two nucleotides, one containing an adenine base and the other a nicotinamide base, joined together through their phosphate groups (see Section 2.3 for details). This cofactor exists in every living cell and is essential for cellular metabolism, energy production, deoxyribonucleic acid (DNA) repair, cell signaling, and functions by accepting/donating electrons in metabolic reactions, while shuttling between its oxidized (NAD^+^) and reduced (NADH) forms.^108,109^ The catalytic turnover rates for formate oxidation in metal-independent Fdhs typically range from 1 to 7 s^–1^, which are considerably lower than those of metal-dependent Fdhs (from several tens to several thousands of s^–1^, see Section 4.2 for details), and they exhibit very low activity for CO_2_ reduction (<1 s^–1^).^107^ CO_2_ reduction by these enzymes is only feasible at very high concentrations of NADH, as this reaction is thermodynamically unfavorable under physiological conditions (pH 7, 298 K), the formal potentials (E°^’^) for the NAD^+^/NADH and CO_2_/formate redox couples are −0.32 V^110,111^ and −0.389 V^61^ vs the standard hydrogen electrode (SHE), respectively.^112–114^

In metal-independent Fdhs, formate oxidation proceeds via direct hydride transfer from formate to the C4 carbon of the nicotinamide moiety of NAD^+^.^37^ The most extensively studied example is the Fdh from *Candida boidinii* (*Cb*Fdh, Figure 2), which is commercially available.^115–117^ These enzymes have been widely applied for NADH regeneration, including at industrial scale.^118–121^ Comprehensive reviews of metal-independent Fdhs are available elsewhere.^122–124^ Given their low CO_2_ reduction activity and the unknown ability to establish DET, they are not discussed further in this review.

**Figure 2:**
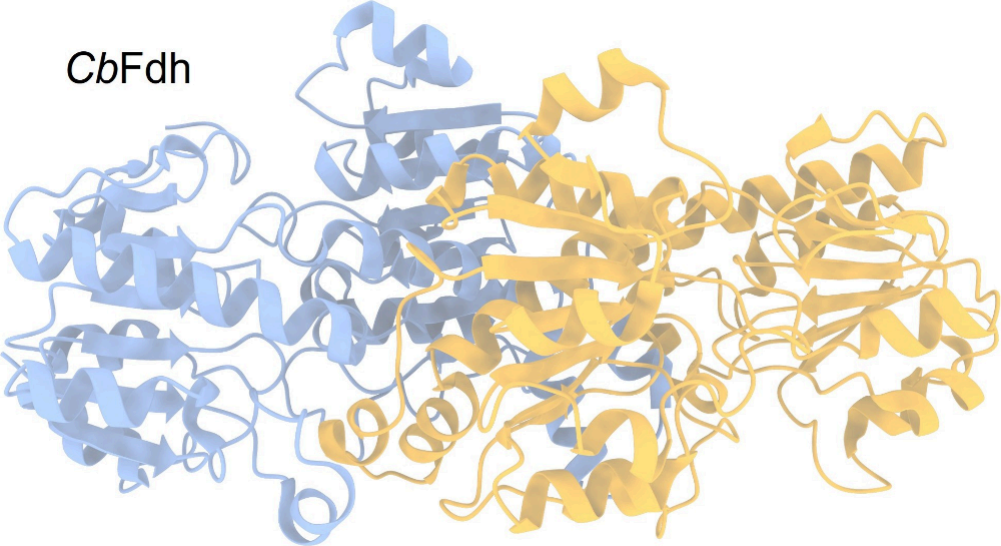
Structure of *Cb*Fdh (PDB: 5DN9).^117^

### 2.2 Metal-Dependent Fdhs

The second group, metal-dependent Fdhs, belongs to the dimethyl sulfoxide (DMSO) reductase family of Mo and W enzymes.^125–127^ This enzyme family is found exclusively in prokaryotes and contains a wide range of redox enzymes,^28,98,107^ including Mo- and W-dependent Fdhs, DMSO reductases, nitrate reductases, and other enzymes. Based on their metal cofactor, metal-dependent Fdhs can be classified into two categories, namely Mo-dependent and W-dependent Fdhs. Notably, W-dependent Fdhs generally exhibit higher CO_2_ reduction rates than their Mo-dependent counterparts,^51,128^ consistent with the more favorable redox properties of W compared to Mo for CO_2_ reduction (see Section 3.1 for redox states).^125^

Although the active site architecture is highly conserved across metal-dependent Fdhs, these enzymes display considerable diversity in their quaternary structures, including variations in subunit number and the composition of redox-active cofactors. Some metal-dependent Fdhs can also use NAD^+^ as an electron acceptor. Examples include Fdhs isolated from *Rhodobacter capsulatus* (*R. capsulatus*) and *Cupriavidus necator* (*C. necator*),^129,130^ which contain a dedicated subunit responsible for NAD^+^ reduction (see Section 2.4.3 for structures).

### 2.3 Metabolic Pathways and Natural Redox Partners

Fdhs can occur either as soluble enzymes located in the periplasm or cytoplasm, or as membrane-bound complexes (Figure 3).^50,98,131–133^ Soluble proteins are freely dispersed in the aqueous cellular environment, whereas membrane-bound proteins are embedded in or tightly associated with cell membranes. The inner membrane forms a selective barrier that encloses the cytoplasm and contains the cell’s primary metabolic and genetic machinery, while the periplasm is an aqueous compartment located between the inner and outer membranes that provides a specialized space for processes such as nutrient transformation and protein folding.

**Figure 3:**
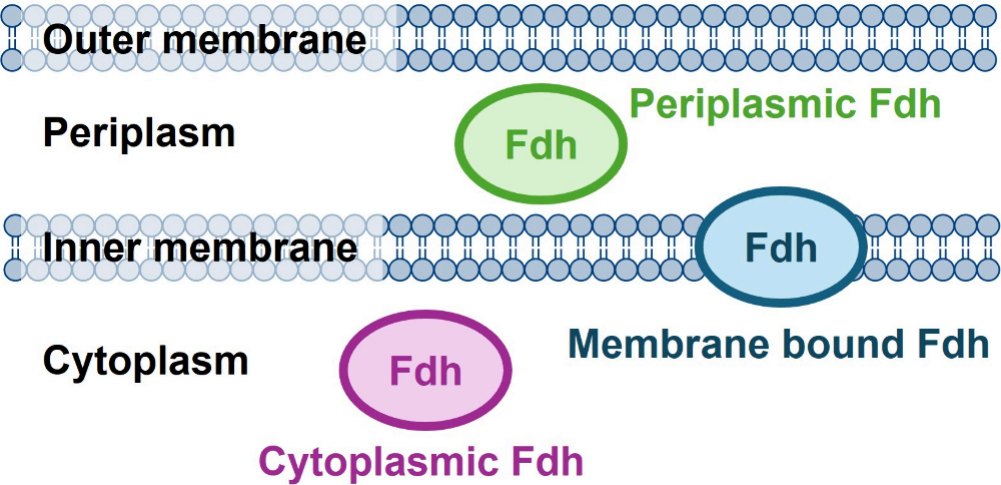
Schematic illustration showing cellular locations of Fdhs.

The unique ability of Fdhs to efficiently catalyze both formate oxidation and CO_2_ reduction enables their involvement in a wide range of metabolic pathways, including fermentation (Figure 4a), formate respiration (Figure 4b), methanogenesis (Figure 4c), acetogenesis (Figure 4d), and NAD^+^ reduction (Figure 4e). Fermentation is an anaerobic metabolic process in which microorganisms break down organic substrates to generate energy, producing simpler molecules such as formate.^134,135^ Formate respiration is a metabolic pathway in which formate is oxidized as an electron donor to drive energy conservation (conserve the energy of nutrient molecules) via an electron transport chain.^136,137^ Methanogenesis is an anaerobic metabolism used by methanogens in which CO_2_, formate, or simple carbon compounds are reduced to methane (CH_4_) as the final product.^138,139^ Acetogenesis is an anaerobic process for energy conservation and carbon fixation in which acetogens reduce CO_2_ to acetate.^140,141^ NAD^+^ reduction is the process by which the oxidized NAD cofactor gains electrons to form NAD^+^, allowing cells to store and transfer reducing power for metabolic and biosynthetic reactions.^142,143^ Fdhs that primarily catalyze CO_2_ reduction are typically cytoplasmic enzymes found in methanogens, acetogens, and some fermentative organisms.^144,145^ In contrast, Fdhs that function mainly in formate oxidation show more diverse cellular localization and can be found in either the cytoplasm or the periplasm.^144,145^

**Figure 4:**
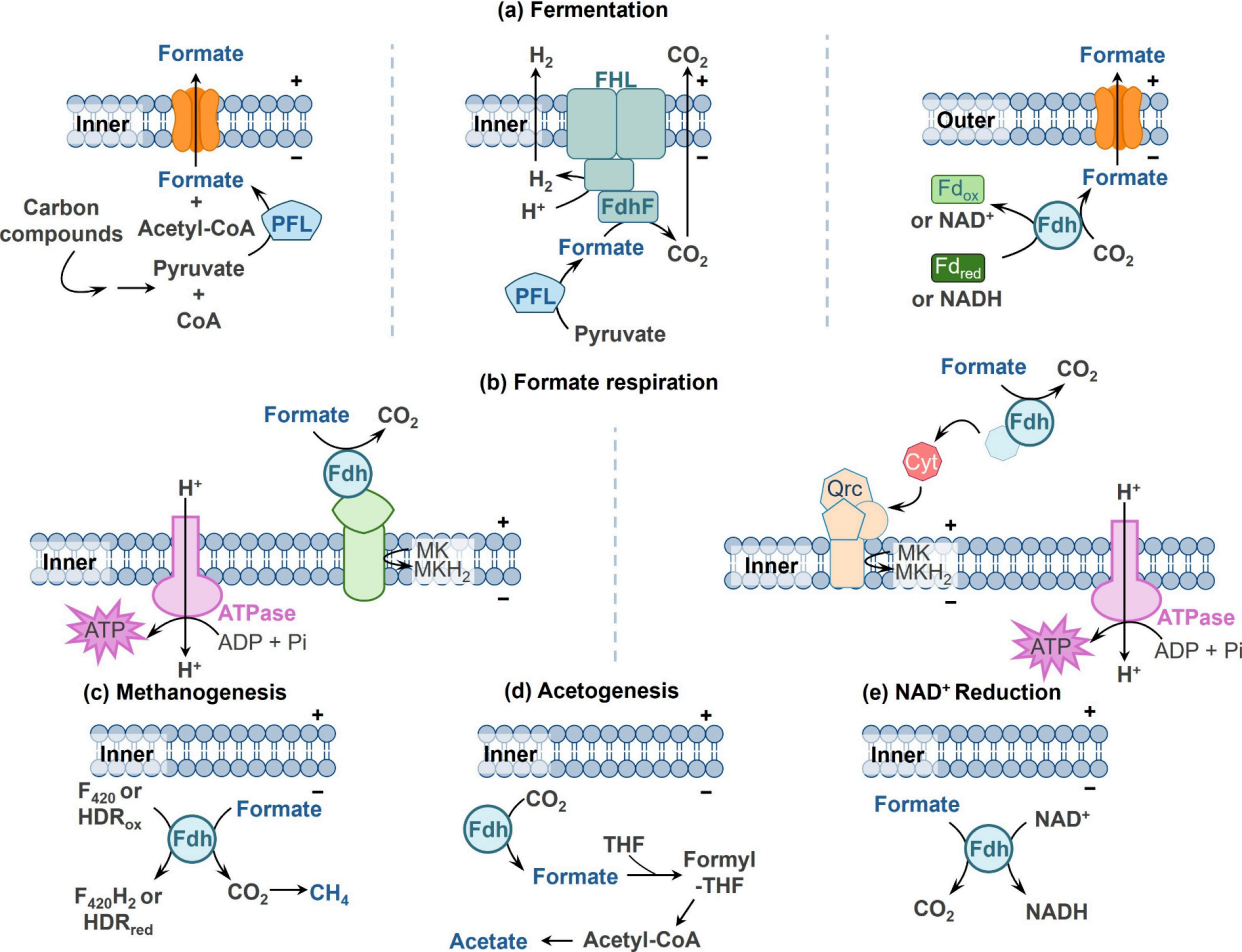
Schematic illustrations of the main metabolic pathways involving Fdhs. Acetyl-CoA: Acetyl-coenzyme A; CoA: coenzyme A; PFL: pyruvate formate lyase; FHL: formate hydrogenlyase; Fd: ferredoxin; MK: menaquinone; MKH_2_: menaquinol; Cyt: cytochrome (TpI*c*_3_); Qrc: quinone reductase complex; HDR: heterodisulfide reductase; Formyl-THF: tetrahydrofolate.

During fermentation, anaerobic or facultatively anaerobic bacteria, such as *Escherichia coli* (*E. coli*) produce formate as a major waste product from pyruvate cleavage (Figure 4a, left panel).^146^ This process is catalyzed by pyruvate formate lyase (PFL, also known as formate C-acetyltransferase),^147^ which reversibly converts pyruvate into formate and acetyl coenzyme A (Acetyl-CoA), a central metabolic intermediate consisting of a two-carbon acetyl group linked to coenzyme A.^148^ To prevent the toxic accumulation of formic acid, which would acidify the cytoplasm and collapse the proton motive force, the soluble Fdh associates with a hydrogenase to form the membrane-bound formate hydrogenlyase (FHL) complex (see Section 2.4.1 for structures).^149–151^ This complex converts formate into CO_2_ and H_2_, effectively transforming an acidic liquid metabolite into neutral gases that readily diffuse out of the cell (Figure 4a, middle panel), thereby helping to maintain intracellular pH during rapid fermentative growth. Periplasmic Fdhs can also catalyze CO_2_ reduction to formate, which serves as an electron shuttle in syntrophic interspecies electron transfer (IET),^128,152^ a cooperative microbial process in which different organisms exchange electrons to break down complex substrates. In this process, CO_2_ reduction is coupled to NADH or ferredoxin oxidation, and the resulting formate is exported across the outer membrane to initiate IET (Figure 4a, right panel).

In the presence of alternative electron acceptors such as nitrate or sulfate, many prokaryotes use formate as an electron donor for anaerobic respiration. In this process, formate is oxidized by Fdh at the periplasmic or cytoplasmic side of the membrane, releasing CO_2_ and two electrons. These electrons are transferred through internal Fe–S clusters to reduce menaquinone (MK) to menaquinol (MKH_2_) within the lipid bilayer (Figure 4b, left panel).^132,153^ MK and MKH_2_ are the oxidized and reduced forms of vitamin K_2_ and act as key membrane-bound electron carriers in bacteria and archaea, shuttling electrons during energy metabolism.^154^ In some organisms, such as sulfate-reducing bacteria (SRB), electron transfer from Fdh to MK is mediated by the quinone reductase complex (Qrc):^133,144,155,156^ electrons released from formate oxidation are first accepted by a soluble periplasmic cytochrome, the type I tetraheme cytochrome *c*_3_ (TpI*c*_3_), passed from this to the Qrc membrane complex, and then transferred to MK (Figure 4b, right panel). The resulting MKH_2_ diffuses within the membrane to deliver electrons to downstream terminal reductases or intermediate complexes. Together, the Qrc complex, the MK/MKH_2_ cycle and downstream complexes, form an electrogenic redox loop that links formate oxidation to the generation of a proton motive force and, ultimately, the synthesis of adenosine triphosphate (ATP). A cytochrome is a heme-containing protein that transfers electrons by reversibly changing the oxidation state of its Fe atom.^157^ Hemes are Fe-containing cofactors in which an Fe atom is coordinated by a tetrapyrrole ring, enabling electron transfer and, in some cases, small-molecule binding.^158^ ATP is the universal energy currency of the cell, storing and supplying energy to drive biochemical reactions such as metabolism, transport, and biosynthesis.^159^

In methanogens, Fdh works in generating reducing equivalents and CO_2_ for the methanogenic pathway and is typically located in the cytoplasm (Figure 4c).^160^ Fdh oxidizes formate to CO_2_ and transfers the released electrons to low-potential electron carriers, most commonly reducing coenzyme F_420_ to F_420_H_2_, and in some organisms also delivering electrons to the heterodisulfide reductase (HDR) complex.^161–163^ Coenzyme F_420_ is a specialized flavin derivative, named for its absorption maximum at 420 nm.^164^ HDR catalyzes the reduction of the heterodisulfide CoM–S–S-CoB back to its thiol forms, which is an essential step for sustaining CH_4_ production.^165^ This heterodisulfide is formed in the final step of methanogenesis and consists of coenzyme M (2-sulfanyl­ethanesulfonate, CoM-SH)^166^ and coenzyme B (7-mercaptoheptanoylthreoninephosphate, CoB-SH)^167^ linked by a disulfide bond. The reduced cofactors generated by Fdh activity, including F_420_H_2_ and reduced HDR, then contribute to the supply of electrons for the multistep reduction of CO_2_ to CH_4_. This tight coupling between Fdh, coenzyme F_420_, and HDR enables methanogens to conserve energy efficiently and sustain growth even under extremely low-energy environmental conditions.^168^

In acetogens, Fdh acts as the primary entry point for inorganic carbon into the Wood–Ljungdahl pathway (WLP), operating in the cytoplasm as a CO_2_ reductase (Figure 4d).^168,169^ The WLP (also known as the reductive acetyl-CoA pathway) is a central anaerobic pathway for carbon fixation that converts CO_2_ into organic carbon,^170–172^ ultimately producing acetate in a process known as acetogenesis. The pathway proceeds through two parallel branches: in the methyl branch, CO_2_ is first reduced to formate by Fdh and then sequentially reduced to a methyl group bound to tetrahydrofolate (Formyl-THF); in the carbonyl branch, a second CO_2_ molecule is reduced to CO by carbon monoxide dehydrogenase. These two units are condensed by Acetyl-CoA synthase to form Acetyl-CoA, which is subsequently converted to acetate with associated ATP formation via substrate-level phosphorylation, where a phosphate group is directly transferred from a high-energy intermediate to adenosine diphosphate (ADP) without the need for a membrane-bound electron transport chain. Through catalyzing the initial CO_2_-to-formate step, Fdh directly links inorganic carbon fixation to cellular energy conservation and biomass formation.

In many bacteria, cytoplasmic NAD^+^-dependent Fdhs play a central role in cellular redox metabolism by oxidizing formate to CO_2_ while simultaneously reducing NAD^+^ to NADH (Figure 4e).^129,130^ The NADH generated provides essential reducing power for carbon fixation, fermentation, and biosynthetic pathways, and helps maintain redox balance under anaerobic or microaerobic conditions. Through this activity, Fdh directly links formate metabolism to the universal NAD^+^/NADH pool that underpins energy conservation and cell growth. In many organisms, this NAD^+^-reducing function also supports survival under nutrient- or energy-limited conditions by ensuring a continuous supply of NADH when alternative electron donors or respiratory pathways are restricted.

Owing to their metabolic versatility *in vivo*, Fdhs can interact with a wide range of natural redox partners to mediate electron transfer under different physiological conditions. These partners include soluble cofactors such as NAD^+^ (Figure 5a), coenzyme F_420_ (Figure 5b), cytochrome *c* (Figure 5c), and ferredoxins (Figure 5d),^162,163,173^ which accept electrons during formate oxidation and channel them into biosynthetic pathways or methanogenesis.^107,174–176^ Membrane-associated redox carriers like quinones (e.g., MK, Figure 5e)^132,153^ and cytochrome *b* (Figure 5f) also act as electron acceptors, thereby coupling Fdh activity to energy conservation through proton or sodium ion gradients. In addition, multiheme cytochrome *c* can serve as electron acceptor for periplasmic Fdhs, for example, the TpI*c*_3_, which transfers electrons onward to the MK pool via the Qrc complex.^144,177^ In some systems, such cytochromes are integral components of the Fdh enzyme itself, forming dedicated electron-transfer subunits, as exemplified by the FdhABC_3_ complex.^133,178^

**Figure 5:**
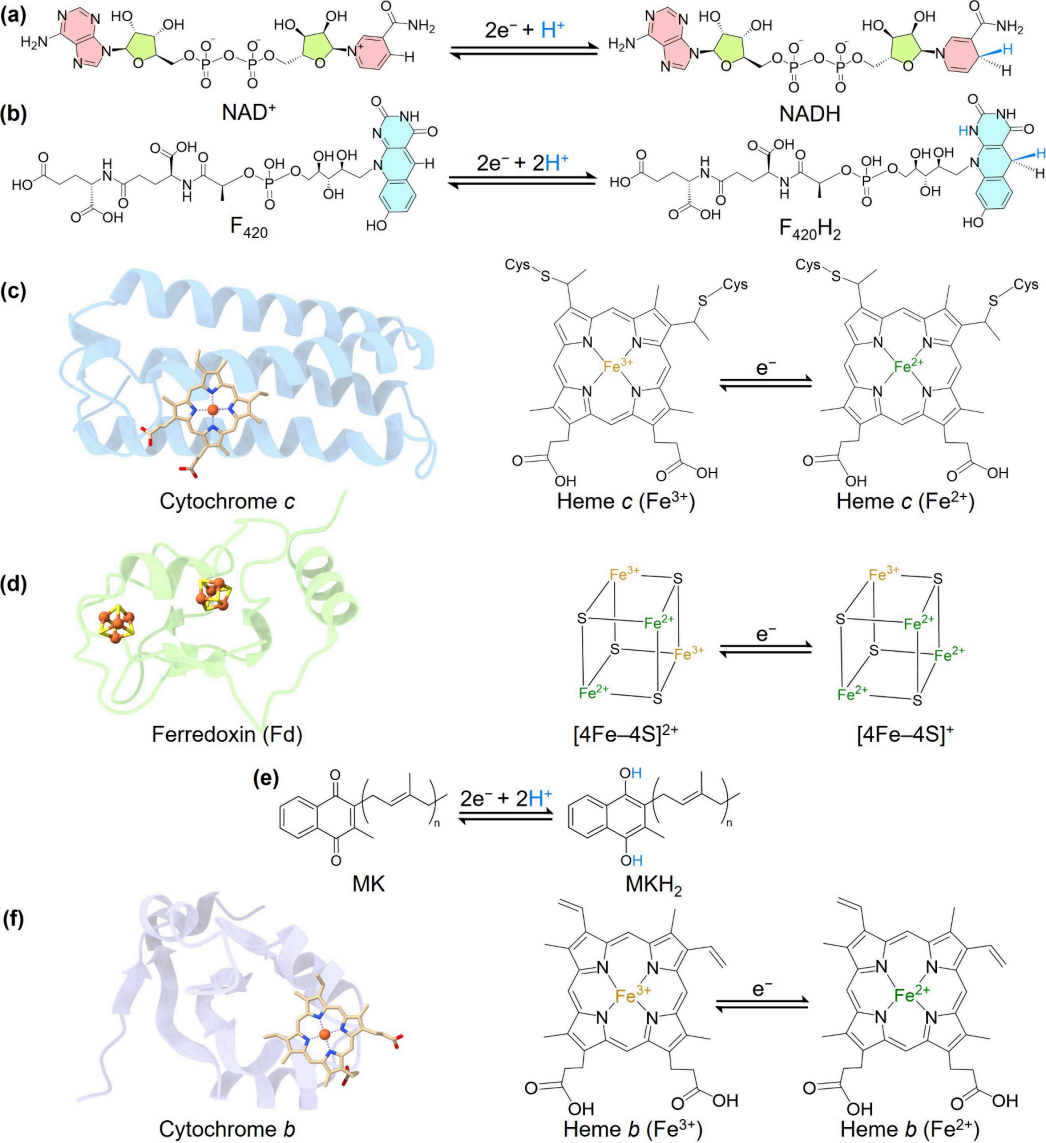
Chemical structures of natural redox partners for Fdh, (a) NAD^+^/NADH, (b) F_420_/F_420_H_2_, (c) cytochrome *c* (PDB: 1CGO)^179^ with its heme *c* center, (d) ferredoxin (PDB: 2ZVS)^180^ with its [4Fe–4S] clusters, (e) MK/MKH_2_, and (f) cytochrome *b* (PDB: 1CYO)^181^ with its Heme *b* center.

Hydrogenases are also natural redox partners of Fdh in pathways that directly link formate and hydrogen metabolism, forming tightly coupled enzyme assemblies such as hydrogen-dependent CO_2_ reductases (HDCRs, Figure 6a) and the FHL complex (Figure 6b). HDCRs catalyze the reversible interconversion of H_2_, CO_2_, and formate, enabling cells to store reducing equivalents in the form of formate or release them as H_2_.^107,131,182^ In contrast, the FHL system plays a key role in maintaining redox balance and pH homeostasis during *E. coli* fermentation by coupling cytoplasmic formate oxidation to proton reduction,^149^ as explained above. Together, HDCR and FHL illustrate how the Fdh and hydrogenase partnerships can provide metabolic flexibility by dynamically interconverting formate, CO_2_, and H_2_ in response to cellular demands. Main cellular locations of Fdhs and respective natural redox partners are listed in Table 1.

**Figure 6:**
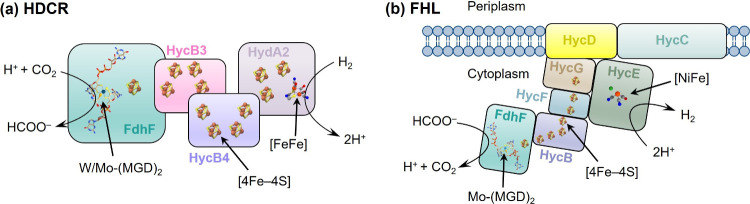
Schematic of the primary *in vivo* role of (a) hydrogen-dependent CO_2_ reductase (HDCR)^182^ and (b) formate hydrogenlyase (FHL) complex,^149,150^ highlighting the interconversion of formate/CO_2_ coupled with H^+^/H_2_.

**Table 1:** Main Cellular Locations of Fdhs and Respective Natural Redox Partners

Location	Redox Partners	ref.
**Cytoplasmic**	NAD^+^	^107^,^162^,^163^,^174–176^
	Ferredoxin, F_420_	
**Cytoplasmic (HDCR)**	Hydrogenase	^131^,^182^
**Inner membrane (FHL)**	Hydrogenase	^149^,^150^
**Inner membrane**	Menaquinone	^132^,^153^
**Periplasmic**	Multiheme cytochrome *c* (independent or subunit)	^133^,^144^,^155^,^178^

### 2.4 Protein Structures and Model Fdhs

Metal-dependent Fdhs exhibit a wide range of structural organizations, spanning from simple single subunit enzymes to large multi subunit complexes.^50,98,131–133^ The number and diversity of cofactors present in Fdhs also vary considerably and, in addition to the catalytic Mo or W center (see Section 3.1 for catalytic mechanism), may include different types of Fe–S clusters, hemes, flavin adenine dinucleotide (FAD), or flavin mononucleotide (FMN).^128,129,133,183^ Fe–S clusters are small assemblies of Fe and S atoms embedded within proteins that function as electron carriers.^184^ Hemes are cofactors in which an Fe atom is coordinated by a tetrapyrrole ring,^158^ as explained in Section 2.3. FAD is an organic cofactor capable of accepting and donating either one or two electrons, while FMN is a smaller flavin cofactor that, like FAD, participates in electron transfer by cycling between oxidized and reduced states in redox enzymes.^185,186^ The key characteristics of some metal-dependent Fdhs are summarized in Table 2.

**Table 2:** Main Characteristics of Model Fdhs and Respective Cellular Locations

Organism	Composition	Cofactor	Location	ref.
***E. coli***	Fdh-H	Sec-Mo	Cytoplasm	^49^,^150^,^187^
	α			
	Fdh-N	Sec-Mo	Membrane	^132^
	αβγ			
	Fdh-O	Sec-Mo	Membrane	^188^,^189^
	αβγ			
***D. desulfuricans***	FdhABC_3_	Sec-Mo	Periplasm	^133^
	αβγ			
***M. gigas***	FdhAB	Sec-W	Periplasm	^190^,^191^
	αβ			
***N. vulgaris*** **Hildenborough**	FdhAB	Sec-W	Periplasm	^51^
	αβ			
	FdhABC_3_	Sec-Mo	Periplasm	^178^,^192^
	αβγ			
	FdhM	Sec-W	Membrane	^192^,^193^
	αβ			
***Shewanella** **oneidensis*** **MR-1**	FdhAB	Cys-W	Membrane	^194^
	αβ			
***S. fumaroxidans***	Fdh1	Sec-W	Periplasm	^61^,^128^
	αβγ			
	Fdh2	Sec-W	Periplasm	^128^
	αβ			
***C. necator***	FdsDABG	Cys-Mo	Cytoplasm	^130^,^175^
	αβγδ			
	NAD^+^-dependent			
***R. capsulatus***	FdsABGD	Cys-Mo	Cytoplasm	^129^,^183^
	αβγδ			
	NAD^+^-dependent			
***B. subtilis***	ForC_4_E_4_	Cys-Mo	Cytoplasm	^195^
***A. woodii***	αβ	Sec-Mo	Cytoplasm (part of HDCR)	^131^
***T. kivui***	αβ	Cys-W	Cytoplasm (part of HDCR)	^182^

#### 2.4.1 *E. coli* Fdhs

The model organism *E. coli* produces three distinct Fdhs, they are Fdh-H (H for hydrogenase-linked), Fdh-N (N for nitrate-inducible), and Fdh-O (O for oxygen-tolerant), named according to their physiological roles. All three enzymes are Mo-dependent and contain Sec coordinated to their active sites, but they differ significantly in their cellular localization, subunit composition, and expression conditions, reflecting their distinct physiological functions for different environmental conditions and metabolic strategies.

Fdh-H is encoded by the *fdhF* gene^196^ and is therefore also referred to as FdhF.^150^ *In vivo*, Fdh-H can associate with the FHL complex (see Section 2.3 for physiological functions).^149^ For clarity, this review consistently uses the designation Fdh-H to refer to the isolated enzyme and FdhF when referring to the subunit of FHL. Fdh-H also occurs as a soluble, cytoplasmic enzyme dissociated from FHL, and represents one of the simplest and best-studied metal-dependent Fdhs,^49,50,187^ consisting solely of a FdhF (α) catalytic subunit that contains one [4Fe–4S] cluster and a Mo cofactor. The structure of the *E. coli* Fdh-H in its isolated form is shown in Figure 7a.^50^

**Figure 7:**
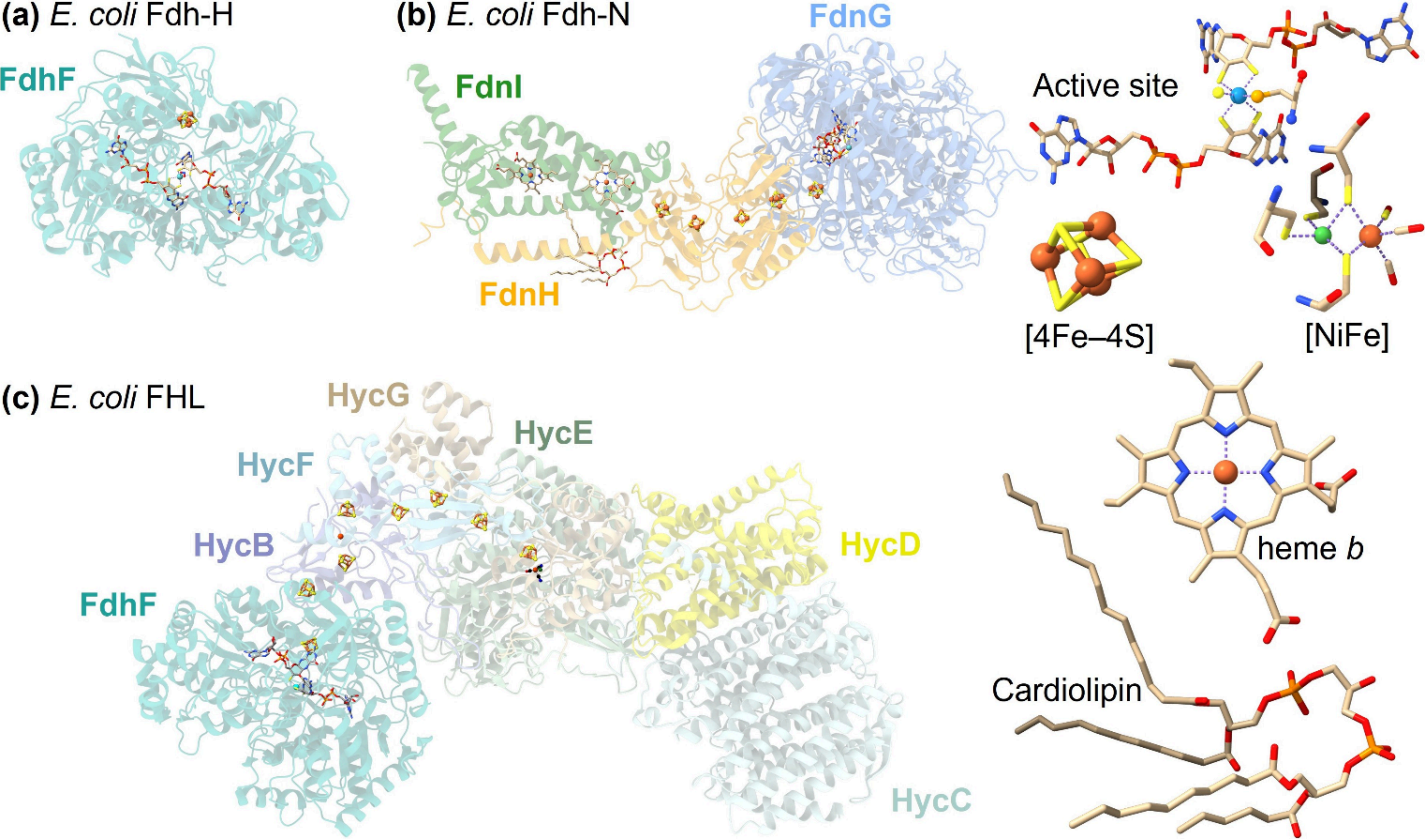
Structures of (a) Fdh-H (PDB: 1FDI),^50^ (b) Fdh-N (PDB: 1KQF),^132^ and (c) FHL (PDB: 7Z0T)^150^ from *E. coli*. Key components are highlighted on the right, including the Fdh Sec-Mo active site, the [NiFe] hydrogenase active site, the [4Fe–4S] cluster, heme *b*, and cardiolipin (1,3-bis­(sn-3′-phosphatidyl)-*sn*-glycerol).

The structure of the complete FHL complex was recently resolved by cryogenic electron microscopy (cryo-EM),^150^ revealing an L-shaped structure with a soluble arm for catalyzing redox reactions and a membrane arm for pumping protons. This is a characteristic structure of the complex I superfamily, a group of evolutionarily related membrane-bound redox enzymes that functions as redox-driven ion pumps.^197,198^ FHL is a heptameric (seven subunits) complex composed of FdhF and six additional subunits (HycB, HycC, HycD, HycE, HycF, and HycG), collectively denoted as FdhF-HycBCDEFG (Figure 7b). The hexameric (six subunits) HycBCDEFG is encoded by the *hyc* genes^199^ and fulfills the hydrogenase and membrane-coupling functions of the system.^150^ In brief, HycE is a [NiFe] hydrogenase that accepts electrons from FdhF, transferred through HycB and HycF, to reduce protons to H_2_, while HycD connects the soluble and membrane arms of the complex. FdhF interacts with the HycB subunit through hydrophobic interactions and hydrogen bonding, and with HycF primarily through electrostatic interactions, enabling efficient electron transfer within the FHL complex.

Fdh-N, encoded by the *fdnGHI* genes,^200^ is the major respiratory Fdh expressed by *E. coli* under anaerobic conditions in the presence of nitrate.^153,201^ This membrane-bound enzyme complex is oriented toward the periplasm and serves as the primary electron-donating component of the formate-nitrate respiratory chain,^202,203^ coupling formate oxidation to nitrate reduction catalyzed by nitrate reductase, a molybdoenzyme that reduces nitrate to nitrite.^204,205^ Electron transfer through this pathway is linked to proton translocation across the membrane by a redox-loop mechanism, generating a proton motive force that drives ATP synthesis and enables energy conservation in the absence of oxygen.^132,153^ The crystal structure of Fdh-N reveals a trimeric (three subunits) complex composed of FdnG, FdnH, and FdnI subunits organized in an (αβγ)_3_ configuration (Figure 7c).^132^ The catalytic FdnG (α) subunit closely resembles Fdh-H and contains a Mo cofactor coordinated by two MGDs, a Sec residue, and a terminal sulfur ligand, along with one [4Fe–4S] cluster.^132^ The FdnH (β) and FdnI (γ) subunits function in electron transfer to MK: the FdnH subunit contains four [4Fe–4S] clusters, while the FdnI subunit contains two heme *b* groups and a MK-binding site that mediates electron transfer to the quinone pool.^132^

Fdh-O is a membrane-associated Fdh that faces the periplasm and is structurally very similar to Fdh-N, sharing a high degree of amino acid sequence identity.^189,201,206^ Unlike Fdh-N, Fdh-O is expressed under both aerobic and anaerobic conditions.^189,201,206^ Its structure is predicted to be trimeric (three subunits), but has not yet been experimentally resolved, and it remains the least studied of the three *E. coli* Fdhs. Available evidence suggests that Fdh-O is coexpressed with a different nitrate reductase and participates in a formate-nitrate respiratory pathway active during the transition from aerobic respiration to anaerobic fermentation.^188,189,206^ This positioning is thought to enable rapid metabolic adaptation when oxygen becomes limiting, allowing cells to quickly redirect electron flow from oxygen to alternative electron acceptors.

#### 2.4.2 *Nitratidesulfovibrio* Fdhs

*Nitratidesulfovibrio* is a relatively newly defined genus of Gram-negative SRB created by reclassifying several species from the broader *Desulfovibrio* genus.^207–209^ These organisms are well-known for expressing high levels of diverse Fdhs and hydrogenases.^144,210,211^ Three species are widely used as sources for Fdh expression: *Nitratidesulfovibrio vulgaris* (*N. vulgaris*, formerly *Desulfovibrio vulgaris* (*D. vulgaris*)), *Megadesulfovibrio gigas* (*M. gigas*, formerly *Desulfovibrio gigas* (*D. gigas*)), and *Desulfovibrio desulfuricans* (*D. desulfuricans*). Originally isolated in Hildenborough, Kent, United Kingdom, *N. vulgaris* Hildenborough (*Nv*H, formerly *D. vulgaris* Hildenborough (*Dv*H)) is a strain of *N. vulgaris* widely used as a model organism for studying anaerobic metabolism, energy conservation, and stress responses in SRB.^212^ The most extensively studied Fdhs from SRB are soluble, periplasmic enzymes rather than membrane-bound complexes, and they typically associate with a TpI*c*_3_ to oxidize formate as an electron donor to the respiratory chain (see Section 2.3 for physiological functions).^144,152,155,177^

*Nv*H expresses three distinct Fdhs: two soluble periplasmic enzymes, FdhAB and FdhABC_3_, and one enzyme that is likely membrane-associated, FdhM.^155,192,207,213^ Among these, FdhAB is the simplest, consisting of two subunits (Figure 8a): a catalytic subunit (FdhA or α) and an electron-transfer subunit (FdhB or β).^51,178^ FdhA contains a W cofactor coordinated by a Sec residue with a histidine (His) and an arginine (Arg) in the second coordination sphere, two MGDs, a terminal sulfur ligand, and one [4Fe–4S] cluster, whereas FdhB contains three [4Fe–4S] clusters that provide efficient electron transfer to the enzyme surface.^51^ The presence of both W and Sec is associated with exceptionally high CO_2_ reduction turnover numbers.^51^ Remarkably, despite being a W-dependent enzyme, FdhAB displays significant oxygen tolerance and can be purified and handled in air. This unusual property arises from an allosteric disulfide bridge that stabilizes an O_2_-protected resting state (see Section 4.3).^214^ Deletion mutant studies have shown that FdhAB is the primary enzyme responsible for CO_2_ reduction under syntrophic and fermentative growth conditions.^152,215^

**Figure 8:**
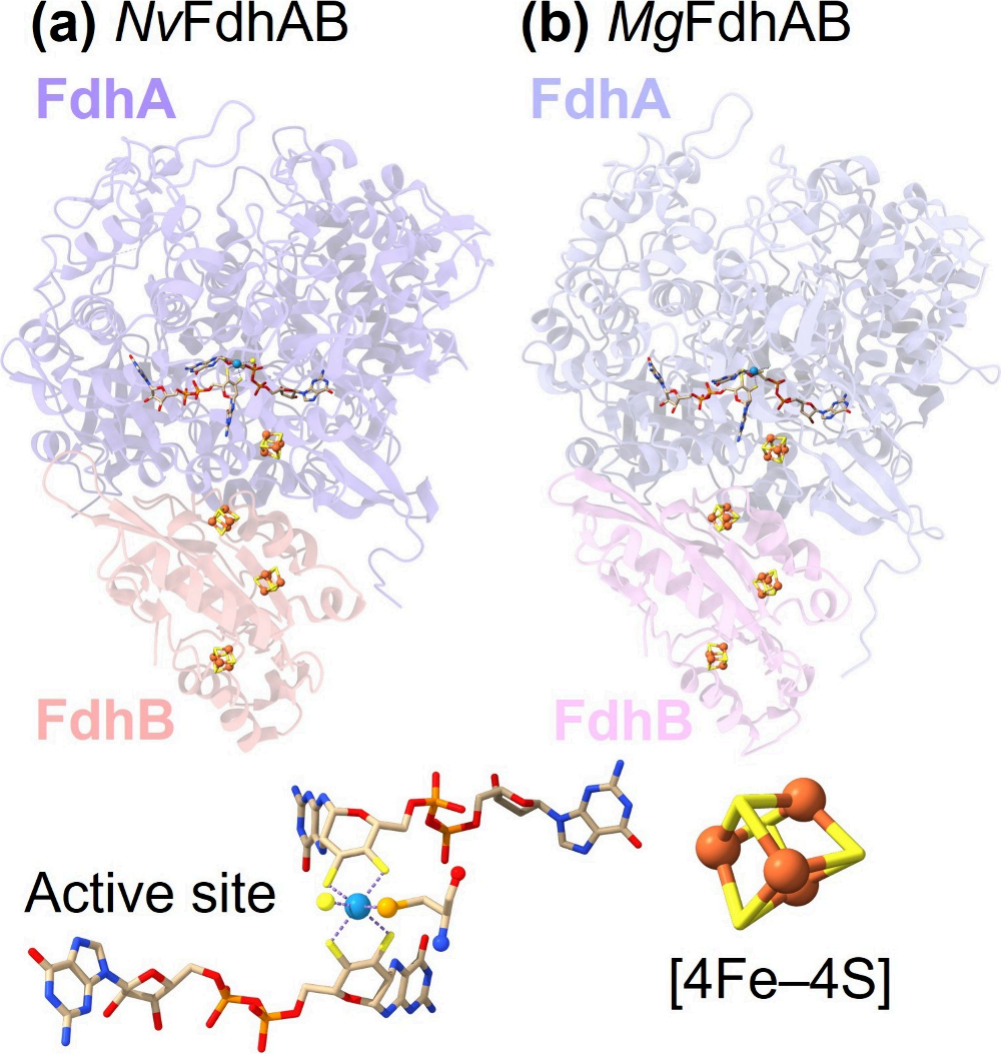
Structures of (a) *Nv*FdhAB (PDB: 6SDV)^51^ and (b) *Mg*FdhAB (PDB: 1H0H).^190^ Key components are highlighted on the bottom, including the Fdh Sec-W active site and the [4Fe–4S] cluster.

The second enzyme, FdhABC_3_, has catalytic (α) and electron-transfer (β) subunits that are closely related to those of FdhAB, but it is Mo-dependent and includes a third subunit: a tetraheme cytochrome *c* that facilitates electron transfer.^178^ The structure of FdhABC_3_ has not been experimentally resolved. FdhAB and FdhABC_3_ are the two main Fdhs expressed by *Nv*H, and their production is tightly regulated by the availability of Mo and W.^192^ Under Mo-rich conditions, FdhABC_3_ is the dominant enzyme, whereas in the presence of W, FdhABC_3_ is downregulated and FdhAB becomes the primary Fdh expressed.^192^ An FdhABC_3_ has also been identified and characterized in *D. desulfuricans*.^133,216,217^

The third enzyme, FdhM, is a Sec-W enzyme that is likely membrane-associated and oriented toward the periplasm.^192^ It is expressed at low levels and is weakly induced by W, and its gene cluster also encodes a multiheme cytochrome *c*, a cytochrome *c* oxidase, and an unidentified membrane protein (i.e., no assigned function).^192^ Cytochrome *c* oxidase catalyzes the reduction of O_2_ to water while simultaneously pumping protons to drive ATP synthesis.^218^ The two FdhM subunits, the catalytic (α) and electron-transfer (β) subunits, can be purified individually,^192^ showing activity toward formate oxidation under aerobic conditions.^106^ However, this activity is approximately 1 order of magnitude lower than that of FdhAB, and no CO_2_ reduction activity has been detected, suggesting that FdhM fulfills a physiological role distinct from those of FdhAB and FdhABC_3_. The structure of FdhM has not been experimentally resolved.

*M. gigas* expresses two Fdhs.^210^ The first is a soluble periplasmic enzyme *Mg*FdhAB composed of two subunits (Figure 8b)^191^ and homologous to *Nv*FdhAB, since it is the same enzyme in a closely related organism. Its structure, solved in 2002, was the first structure reported for a W-dependent enzyme from a mesophilic organism.^190^ This structure revealed a Sec-W active site, with one [4Fe–4S] cluster in the catalytic (FdhA or α) subunit and three [4Fe–4S] clusters in the electron transfer (FdhB or β) subunit. The second Fdh has not yet been biochemically characterized, but sequence analysis suggests it is a close homologue of the first enzyme, also comprising two similar subunits, with a Cys residue replacing Sec as the metal-coordinating ligand.^210^

#### 2.4.3 *Shewanella oneidensis* MR-1 Fdhs

A W-containing Fdh from *Shewanella oneidensis* MR-1 (*So*FdhAB) was identified via AI-assisted enzyme mining and exhibits both oxygen tolerance (see Section 4.3 for details) and intrinsic DET capability (see Section 5.1 for details).^194^ The enzyme was expressed in a dimeric (two subunits) architecture composed of a catalytic subunit (FdhA or α) and an electron transfer subunit (FdhB or β). FdhA contains a Cys-W cofactor with a terminal sulfur ligand, together with one [4Fe–4S] cluster. FdhB accommodates four [4Fe–4S] clusters, forming an extended electron transfer chain (Figure 9). Compared to the homologous dimeric (two subunits) enzyme *Nv*FdhAB (see Section 2.4.2 for details), *So*FdhA and *So*FdhB share modest sequence identities with *Nv*FdhA (30.5%) and *Nv*FdhB (22.5%). Notably, *So*FdhAB represents the first Fdh reported to combine complete oxygen tolerance with DET.^194^

**Figure 9:**
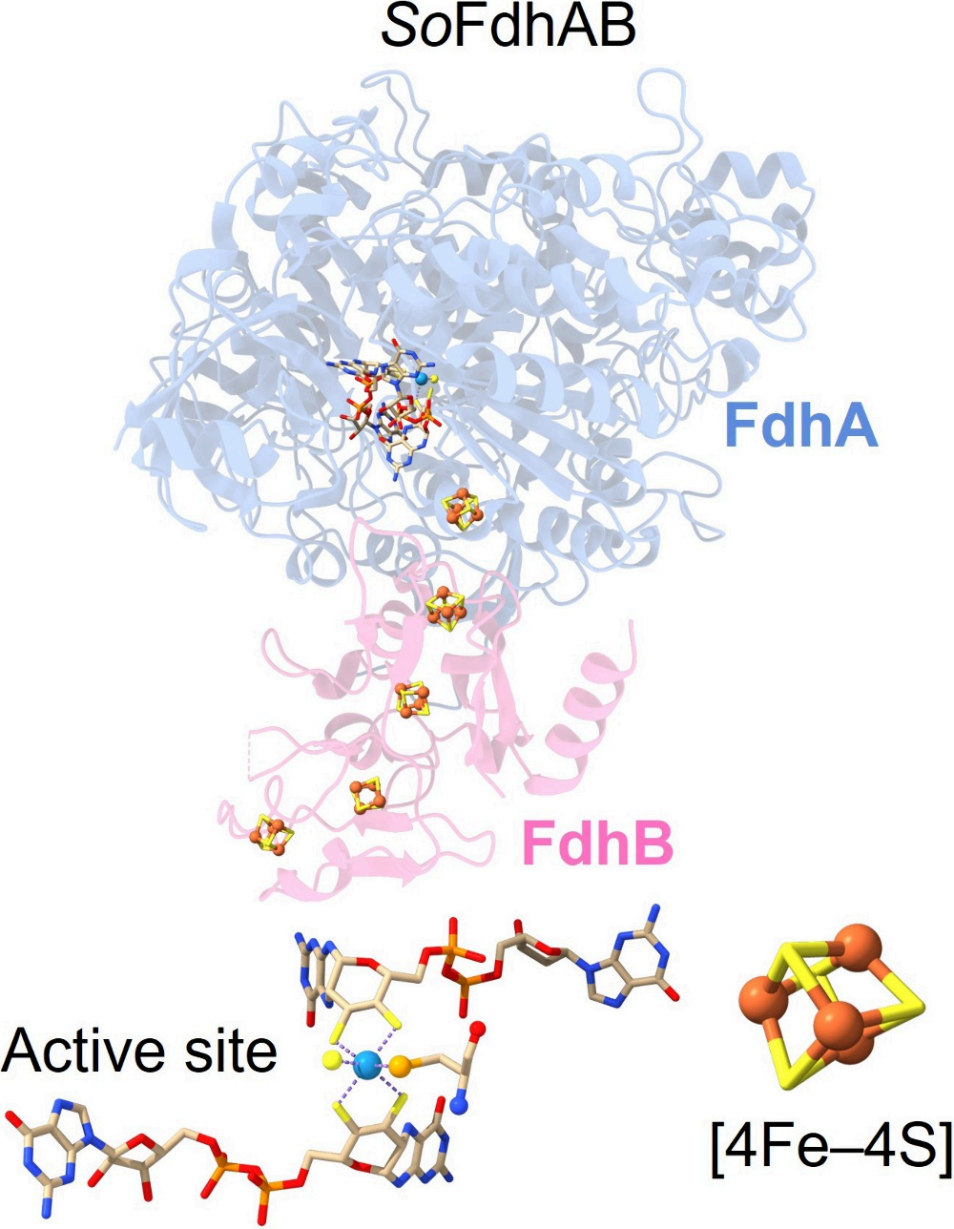
Structure of *So*FdhAB (PDB: 9VAP).^194^ Key components are highlighted on the bottom, including the Fdh Cys-W active site and the [4Fe–4S] cluster.

#### 2.4.4 Cytoplasmic NAD^+^-Dependent Fdhs

A distinct group of metal-dependent cytoplasmic Fdhs use NAD^+^ as their physiological electron acceptor. These enzymes are found in several bacteria and operate in diverse metabolic contexts, where they contribute to NADH generation for cellular redox balance and energy metabolism. They are typically dimeric (two subunits) or multimeric complexes containing multiple Fe–S clusters and a dedicated NAD^+^-reducing subunit that binds an FMN or FAD cofactor. Three representative systems have been studied in detail: *Me*FDH1 from *Methylorubrum extorquens* (*M. extorquens*) AM1, FdsDABG from *C. necator*, and FdsABGD from *R. capsulatus*.

*M. extorquens* AM1 is a pink Gram-negative bacterium widely used as the primary model organism for studying methylotrophy, the metabolic ability to grow aerobically on one-carbon (C1) compounds such as methanol and methylamine, with formate acting as a central metabolic intermediate and, in some conditions, a carbon and energy source. In this organism, *Me*FDH1 catalyzes the oxidation of formate produced during the metabolism of reduced C1 compounds such as CH_4_, methanol, or methylamine.^174^ This enzyme is dimeric (two subunits), composed of a catalytic (Fdh1A or α) subunit and a dedicated NAD^+^-reducing (Fdh1B or β) subunit (Figure 10a). The catalytic subunit contains a Cys-vW cofactor, three [4Fe–4S] clusters, and one [2Fe–2S] cluster, while the NAD^+^-reducing subunit binds one FMN cofactor, one [4Fe–4S] cluster, and one [2Fe–2S] cluster to facilitate electron transfer to NAD^+^. The structure of *Me*FDH1 has been resolved by cryo-EM.^219,220^

**Figure 10:**
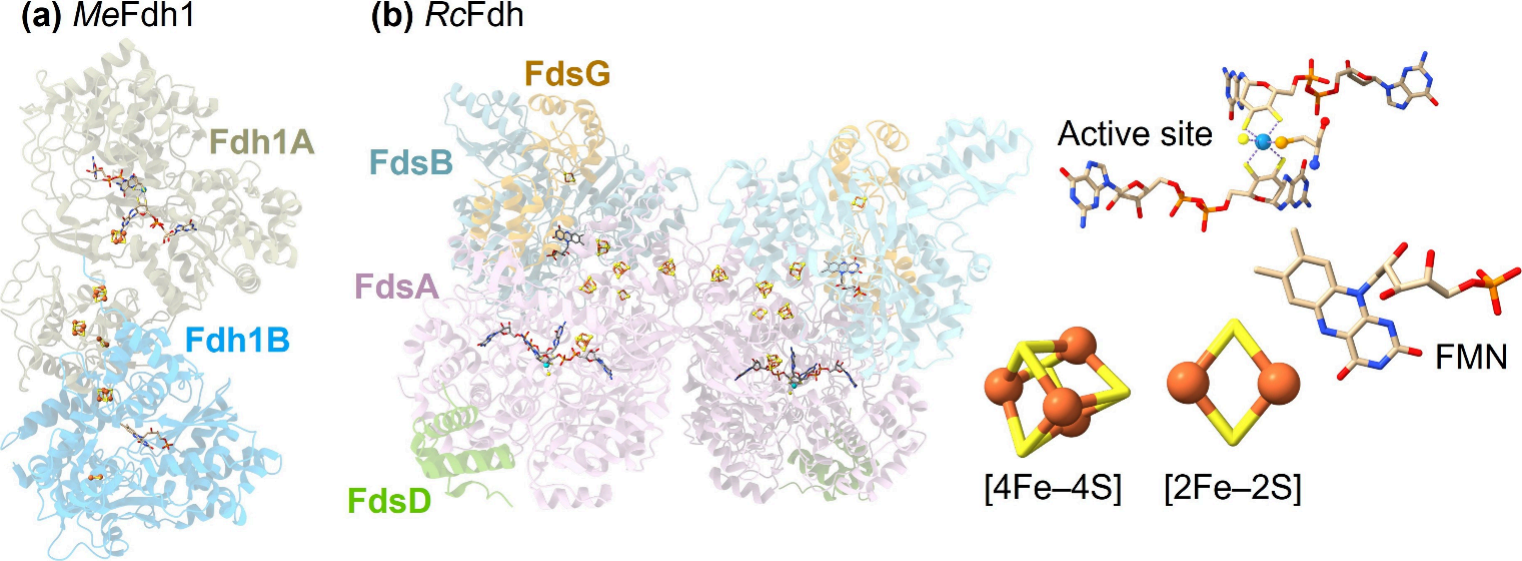
Structures of (a) *Me*Fdh1 (PDB: 7XQW)^17^ and (b) *Rc*Fdh dimer (PDB: 6TGA).^183^ Key components are highlighted on the right, including Cys-W active site for *Me*Fdh1, Cye-Mo active site for *Rc*Fdh, the [4Fe–4S] and [2Fe–2S] clusters, and FMN.

*C. necator* is a Gram-negative, aerobic, H_2_-oxidizing bacterium that is widely used as a model organism for studying the biosynthesis of biodegradable plastics, particularly polyhydroxy­alkanoates (PHAs).^221^ This organism expresses a tetrameric (four subunits), NAD^+^-dependent Fdh known as FdsDABG (*Cn*Fdh), where the designation Fds reflects that the enzyme is encoded by the *fds* gene, with the letter ‘s’ indicating its soluble nature.^130,175,222,223^ The redox potentials of these subunits have been characterized recently.^224^

*R. capsulatus* is a purple nonsulfur, Gram-negative bacterium noted for its remarkable metabolic versatility and ability to grow under both aerobic and anaerobic conditions. It is widely used as a model organism for studying photosynthesis, nitrogen fixation, and cellular electron-transfer processes.^225,226^ *R. capsulatus* expresses a similar tetrameric (four subunits), NAD^+^-dependent Fdh termed FdsABGD (*Rc*Fdh), which is homologous to the *C. necator* enzyme *Cn*Fdh.^54,129^ Structural studies by cryo-EM revealed that the catalytic FdsA subunit contains a Cys-coordinated Mo cofactor and one [2Fe–2S] cluster, along with four additional [4Fe–4S] clusters involved in intramolecular electron transfer. The FdsB subunit harbors one [4Fe–4S] cluster and an FMN cofactor that mediates NAD^+^ reduction, while the FdsG subunit contains one [2Fe–2S] cluster. The FdsD subunit does not bind cofactors but caps the Mo-containing domain of FdsA, contributing to structural stability and enzyme assembly (Figure 10b).^183^ Electron transfer has been shown to occur within each FdsABGD tetramer rather than between tetramers.^227^

Both Fdhs from *C. necator* and *R. capsulatus* are tailored for formate oxidation under physiological conditions, but they have also been shown to catalyze CO_2_ reduction when supplied with high concentrations of NADH, enabling *in vitro* NADH recycling.^129,175,228^ However, this reverse reaction proceeds at only moderate rates, as it is thermodynamically unfavorable and strongly limited by product inhibition, particularly by formate and NAD^+^ accumulation.^228^ These constraints indicate that CO_2_ reduction is unlikely to represent a significant physiological function for these enzymes, but rather reflects their intrinsic catalytic reversibility under artificially driven reducing conditions.

#### 2.4.5 *Bacillus subtilis* Fdhs

The observation that the Gram-positive bacterium *Bacillus subtilis* (*B. subtilis*) presents formate oxidation activity when grown aerobically, led to the identification of two noncanonical Fdhs, ForCE1 and ForCE2.^229^ These *Bs*Fdhs catalyze formate oxidation and are composed of two subunits: the catalytic subunit ForC and the essential partner subunit ForE.^195^ The structure of the asymmetric dimer unit of a complete ForCE hetero-octamer (eight subunits) is shown in Figure 11. The ForC subunit functions as the formate oxidoreductase and contains a Cys-Mo, together with four [4Fe–4S] clusters and one [2Fe–2S] cluster. Its amino acid sequence shows high homology to the catalytic subunit FdsA from *R. capsulatus* and *C. necator* (see Section 2.4.4 for details). In contrast, the ForE subunit does not directly participate in catalysis but is essential for enzyme function. It forms a tight complex with ForC and plays a structural and functional role in mediating electron transfer from formate oxidation to the membrane quinone pool. In particular, ForE contributes to the stabilization of quinone binding and to the overall structural integrity of the complex, as the catalytic activity of ForC is significantly diminished in the absence of ForE.^229^ Structural insights from cryo-EM and modeling suggest that the interface between ForC and ForE contains small cavities that are occupied by glycerophospholipid molecules. These lipids are proposed to seal the intersubunit interface and may facilitate transient association of the complex with the cytoplasmic membrane.^195,229^

**Figure 11:**
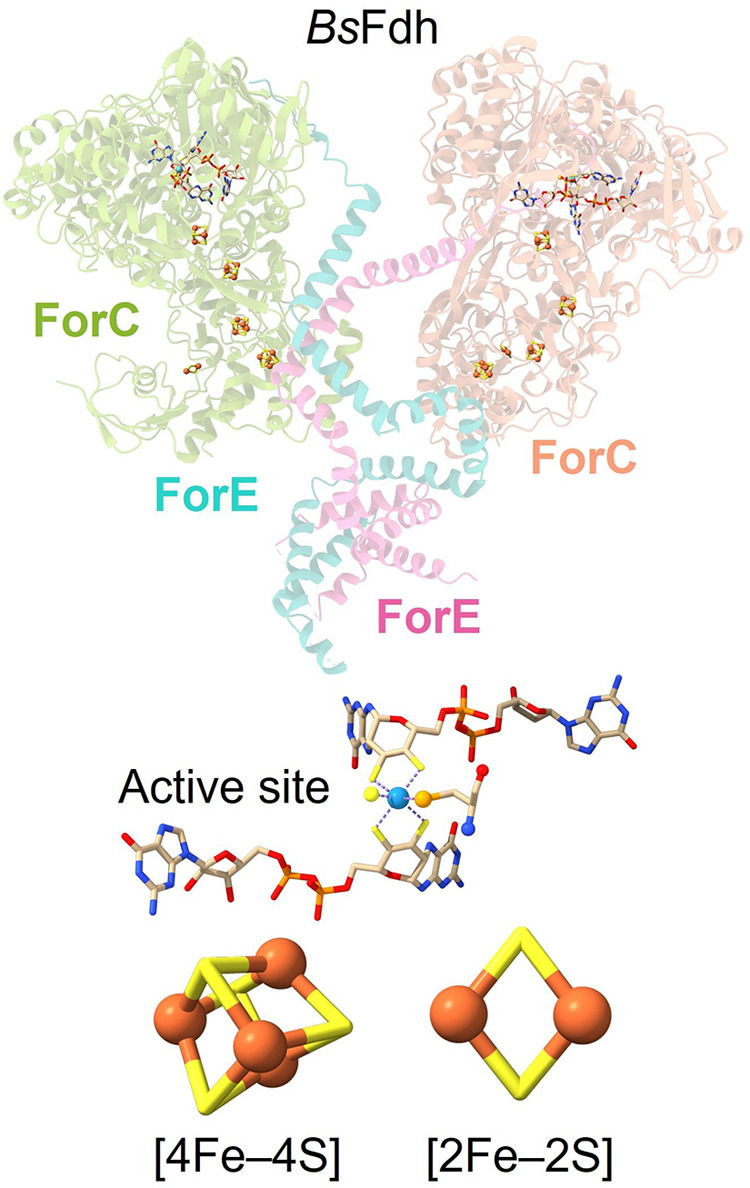
Structure of the asymmetric dimer unit of ForCE1 from *B. subtilis* (PDB: 8RQZ).^195^ Key components are highlighted on the bottom, including the Fdh Cys-Mo active site, the [4Fe–4S] cluster, and the [2Fe–2S] cluster.

#### 2.4.6 Hydrogen Dependent CO_2_ Reductases (HDCRs)

A type of Fdh has recently been characterized as part of HDCR, a multienzyme complex in which a hydrogenase is directly coupled to an Fdh to catalyze the reduction of CO_2_ to formate driven by H_2_ oxidation (see Section 2.3 for physiological functions).^131^ To date, only two HDCRs have been studied in detail, both from acetogenic bacteria in which the enzyme plays a central role in CO_2_ fixation: *Acetobacterium woodii* (*A. woodii*) and *Thermoanaerobacter kivui* (*T. kivui*).^131,182^

The HDCR from *A. woodii* (*Aw*HDCR) was the first to be characterized.^131^ It is a tetrameric (four subunits) complex comprising a FdhF subunit featuring a Sec-Mo cofactor, a HydA2 subunit with an [FeFe] hydrogenase, and two electron transfer subunits HycB2 and HycB3. *Aw*HDCR contains a total of 11 [4Fe–4S] clusters, with one located in the FdhF subunit, two in the HydA2 subunit, and four in each of the two electron transfer subunits HycB2 and HycB3, forming an extended intramolecular electron transfer pathway. The thermophilic HDCR from *T. kivui* (*Tk*HDCR) has a similar tetrameric (four subunits) structure containing FdhF, HydA2, HycB3 and HycB4 (Figure 12), but it differs from *Aw*HDCR in two key aspects. First, the FdhF subunit contains a Cys-coordinated W cofactor instead of Mo, and second, the HydA2 subunit harbors three [4Fe–4S] clusters, resulting in a total of 12 [4Fe–4S] clusters in *Tk*HDCR.^182,230^ Recently, the structure of *Tk*HDCR was resolved by cryo-EM, revealing that the complex assembles into long filaments *in vivo*, a supramolecular organization that enhances electron transfer efficiency and overall catalytic activity.^230^ Notably, in addition to H_2_-driven CO_2_ reduction, both HDCRs can perform H_2_ production from formate oxidation, and the Fdh and hydrogenase can also accept electrons from reduced ferredoxin to support CO_2_ or proton reduction.^231^

**Figure 12:**
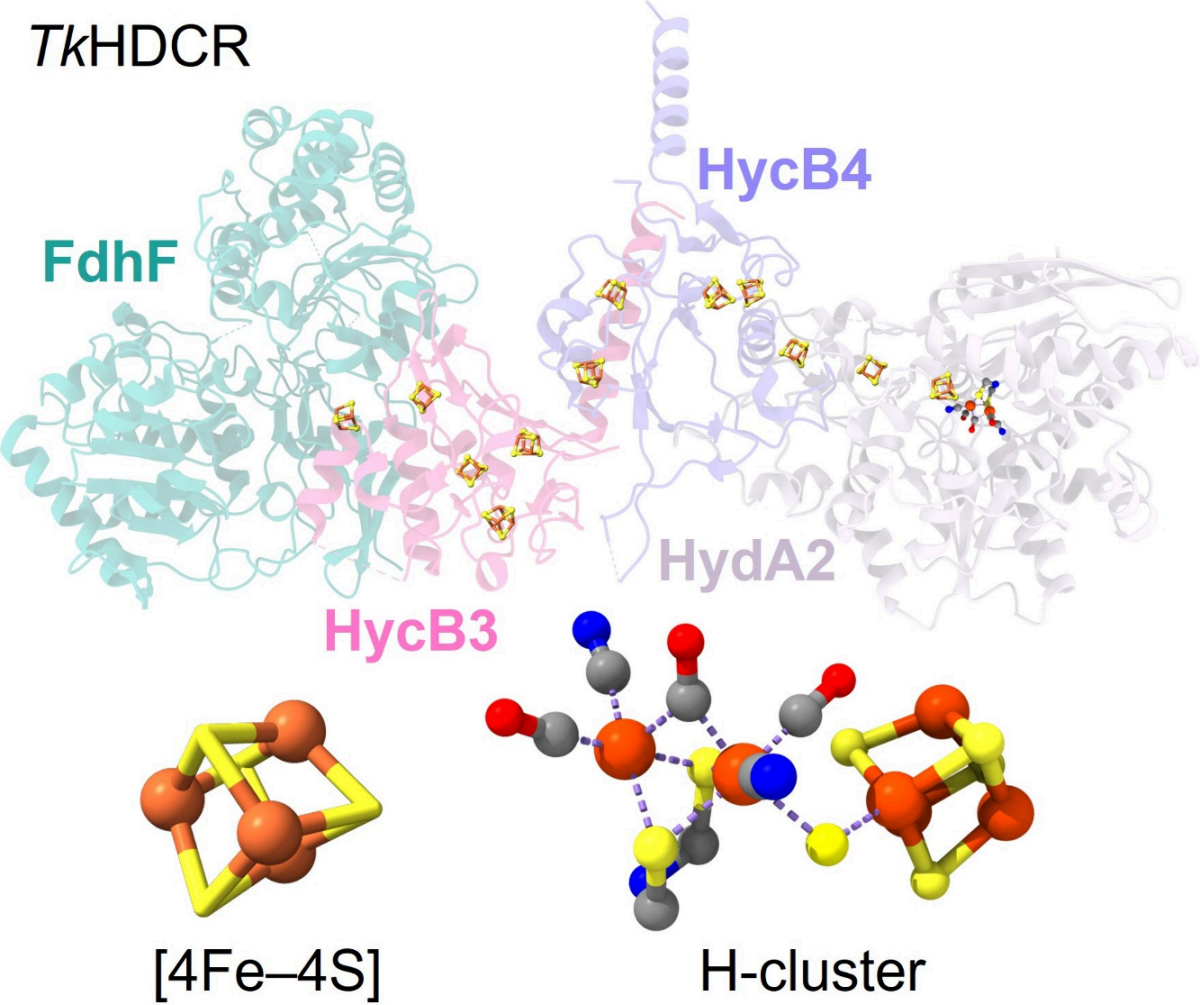
Structure of the asymmetric unit of *Tk*HDCR (PDB: 7QV7).^230^ Note that the W cofactor in FdhF is not resolved due to the resolution limit of the cryo-EM structure. Key components are highlighted on the bottom, including the [4Fe–4S] cluster, and the H-cluster.

#### 2.4.7 Formylmethanofuran Dehydrogenases (FMFdhs)

A formylmethanofuran dehydrogenase (FMFdh) is a Mo- or W-containing enzyme from methanogenic archaea that catalyzes the reduction of CO_2_ to formate and its subsequent condensation with methanofuran to form formylmethanofuran, using ferredoxin as the physiological electron donor.^161,232,233^ The crystal structure of FMFdh from *Methanothermobacter wolfeii* (FwdABCDFG) was resolved in two forms, as a dimer (two subunits) and as a tetramer (four subunits) of the FwdABCDFG hexamer (six subunits), revealing that catalysis is initiated by CO_2_ reduction to formate at an Fdh-like subunit (Figure 13).^232^ This complex contains twenty-four subunits with a total of forty-six [4Fe–4S] clusters that electronically couple the two catalytic modules: FwdB, a Cys-W Fdh subunit responsible for CO_2_ reduction, and FwdA, an amidohydrolase subunit harboring a binuclear Zn^2+^ center that catalyzes the condensation of formate with the methanofuran cofactor. Note that the metal cofactor is not resolved in Figure 12 due to the resolution limit of the cryo-EM structure, and it was modeled by comparing to the structure of *Mg*FdhAB. Remarkably, the W active site in FwdB is connected to the solvent by a narrow 40 Å long tunnel that permits access of CO_2_ but excludes formate to enforce directional catalysis. The formate generated at this site is then channeled to the FwdA active site through a separate 43 Å long hydrophilic tunnel linking the two catalytic centers. The remaining subunits play predominantly structural and electron transfer roles: FwdF contains eight [4Fe–4S] clusters and FwdG contains two [4Fe–4S] clusters to mediate long-range electron transfer, while FwdC forms a central scaffold that interfaces with FwdB, FwdA, and FwdF, stabilizing the overall architecture of the complex.^232^

**Figure 13:**
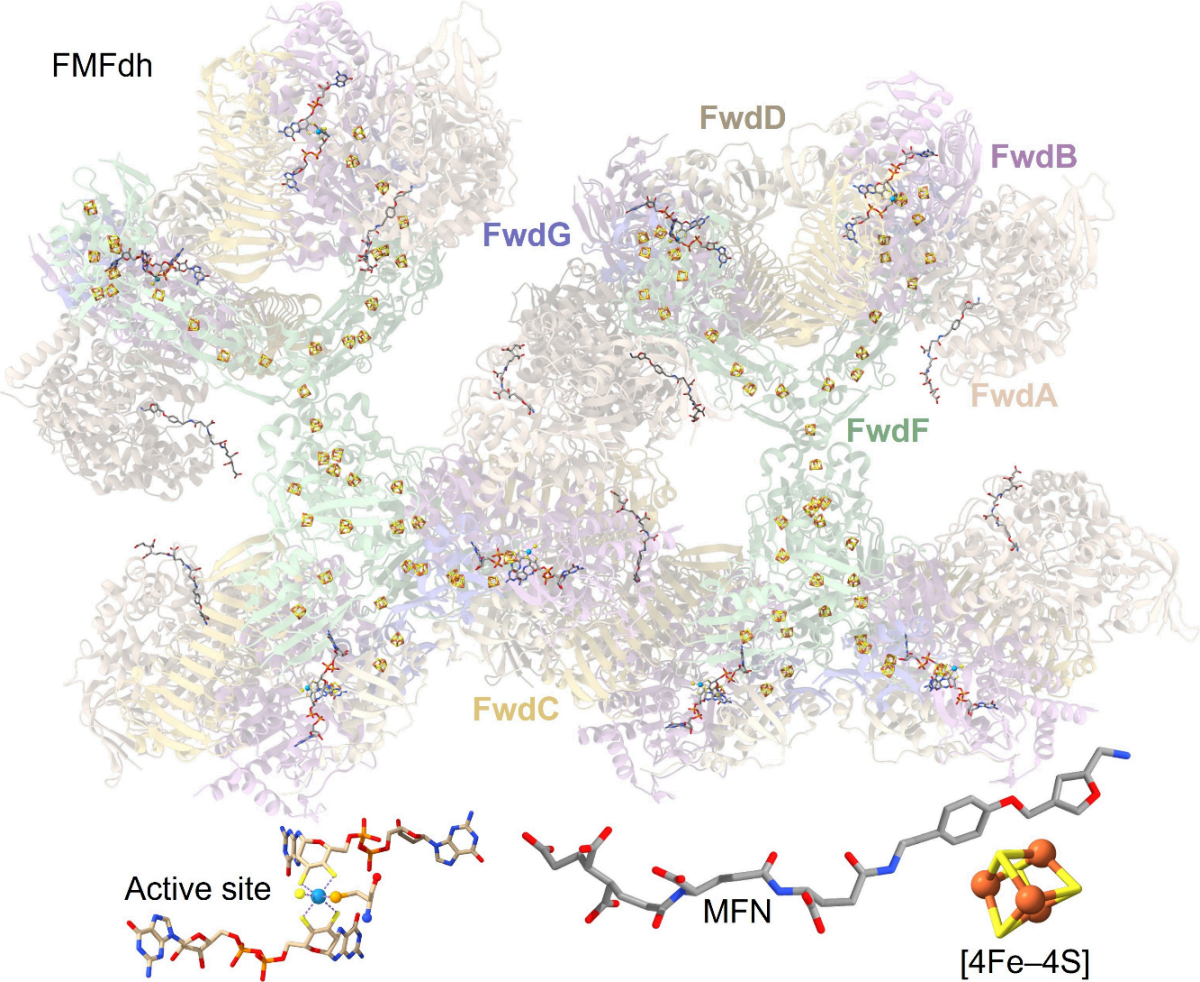
Structure of dimer form of the FwdABCDFG hexamer in FMFdh (PDB: 5T61).^232^ Key components are highlighted on the bottom, including the Fdh Cys-W active site, the [4Fe–4S] cluster, and MFN (N-[4,5,7-tricarboxyheptanoyl]-l-gamma-glutamyl-N-{2-[4-({5-[(formylamino)­methyl]-3-furyl}­methoxy)­phenyl]­ethyl}-d-glutamine).

### 2.5 Protein Evolution

Fdhs are considered among the most ancient enzymes within the DMSO reductase superfamily and are thought to have been vertically inherited from the last universal common ancestor (LUCA), the most recent organism from which all known life on Earth descended, which marks the branching point for Bacteria, Archaea, and Eukarya.^234,235^ Fdhs featuring Sec as a metal-coordinating ligand^127,236,237^ are the most common selenoproteins in the genomes of Sec-utilizing bacteria and archaea.^238^ The presence of both periplasmic and cytoplasmic Fdhs across the bacterial and archaeal domains suggests that LUCA already possessed distinct Fdhs adapted for periplasmic formate oxidation and cytoplasmic CO_2_ reduction prior to the divergence of these domains.^127^

Fdhs can be broadly divided into two major phylogenetic groups that correlate with their cellular localization, quaternary structure, and physiological roles. The first group comprises cytoplasmic Fdhs, including Fdh-H, methanogenic Fdhs that oxidize formate to CO_2_ during methanogenesis, acetogenic Fdhs that catalyze CO_2_ reduction to formate as the first step of acetogenesis, and multimeric NAD^+^-dependent Fdhs with complex architectures, such as those from *R. capsulatus* or *C. necator*.^129,130,175,183^ These enzymes are typically soluble, often multisubunit complexes, and are closely integrated into central redox metabolism through various natural redox partners. The second phylogenetic group consists of periplasmic or periplasmic-facing membrane Fdhs,^127,236^ exemplified by the Mo-dependent Fdh-N and Fdh-O from *E. coli*.^132^ These enzymes are usually trimeric (three subunits), comprising a catalytic subunit, an electron transfer subunit, and an integral membrane subunit that mediates electron transfer to the quinone pool. This group also includes periplasmic Fdhs from SRB, such as the dimeric (two subunits) FdhAB from *Nv*H^51,214,239^ and related organisms,^190,191^ as well as trimeric (three subunits) enzymes like FdhABC_3_ from *D. desulfuricans* and *Nv*H, which contain an additional tetraheme cytochrome *c* subunit.^133,178,216^ In these SRB, the Fdhs lack an integral membrane subunit and instead transfer electrons to the respiratory chain via the TpI*c*_3_ and the Qrc complex.^144,155^

The evolution of metal-dependent Fdhs is closely linked to the bioavailability of metal elements in the environment. Although Mo and W have similar average abundances in the Earth’s crust (1.2 mg kg^–1^ for Mo and 1.25 mg kg^–1^ for W), their concentrations in seawater differ markedly, at approximately 0.01 mg L^–1^ for Mo ions and 0.0001 mg L^–1^ for W ions.^240–242^ Importantly, elemental abundance does not directly translate into biological availability, which is strongly influenced by environmental conditions and has likely shaped the evolution of this enzyme family.

The rise of atmospheric oxygen and the associated oxidation of Mo to the highly soluble molybdate ion may have favored the incorporation of Mo over the more ancient W in the active sites of some enzymes.^125,243^ Consistent with this view, Mo-dependent Fdhs are found in both anaerobic and aerobic prokaryotes, as well as in facultative anaerobes,^129,130,216^ whereas W-dependent Fdhs are predominantly restricted to obligate anaerobes.^51,128,244^ A notable exception is the aerobic methylotrophic *Methylobacterium extorquens*,^174^ in which the presence of a W-dependent Fdh is likely the result of horizontal gene transfer, a process by which an organism acquires genetic material directly from another organism rather than inheriting it from a parent,^245^ thereby enabling the rapid acquisition of new traits such as antibiotic resistance.

In addition, W is strictly essential for some hyperthermophilic organisms, possibly reflecting its higher bioavailability in ancient anoxic environments, such as sulfide-rich waters where early life may have emerged. Under these conditions, W sulfides are significantly more soluble than Mo sulfides, rendering Mo comparatively poorly bioavailable.^246^ Finally, the expression of Fdhs in several organisms is differentially regulated by the presence of Mo or W ions, highlighting the continued influence of metal availability on Fdh biology.^192,247^

## 3 Catalytic Mechanisms of Fdhs

It is generally assumed that the interconversion of CO_2_ and formate proceeds through a reversible catalytic mechanism.^36^ Under this framework, mechanistic insights gained from formate oxidation are directly relevant to understanding CO_2_ reduction, and vice versa. Although Fdhs display notable diversity at their active sites including variations in the Mo or W center, Sec or Cys ligand, and surrounding amino acid residues, it is widely accepted that these enzymes operate via fundamentally similar catalytic mechanisms, despite exhibiting different catalytic efficiencies for formate oxidation and CO_2_ reduction, defined by the ratio between turnover number and the Michaelis constant (see Section 4.2 for Fdh catalysis). Accordingly, differences in activity among Fdhs are best viewed as tuning of overall catalytic activity rather than reflecting distinct mechanistic pathways. Several mechanistic hypotheses have been proposed and comprehensively reviewed elsewhere.^98,248,249^ In this section, we highlight key structural features of Fdh active sites, summarize proposed catalytic intermediates, and discuss experimental evidence that informs current understanding of active site structure and reactivity.

### 3.1 CO_2_ and Formate as the Substrates

Experimental evidence indicates that Fdh catalyzes the interconversion between CO_2_ and formate, rather than involving bicarbonate (HCO_3_^–^) as a direct substrate or product (Figure 14).^250^ Early studies on Fdh from *Clostridium pasteurianum* showed that incorporation of ^14^C into formate occurs more rapidly when ^14^CO_2_ is supplied than when than when H^14^CO_3_^–^ is used.^251^ When carbonic anhydrase, a zinc-containing enzyme that catalyzes the interconversion of CO_2_ and water with bicarbonate and protons, is added to the assay, the uptake rates from ^14^CO_2_ and H^14^CO_3_^–^ become identical.^251^ Electrochemical measurements further support this conclusion, demonstrating an immediate catalytic response upon addition of CO_2_, whereas bicarbonate addition produces a delayed increase in the reduction rate as reflected by the electrocatalytic current (see Section 5.2).^250,252,253^ Together, these observations indicate that Fdhs do not directly react with HCO_3_^–^, and instead catalyze CO_2_ reduction.

**Figure 14:**
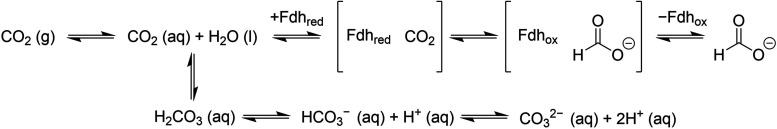
Schematic illustration of how Fdhs reduce CO_2_ to formate, and oxidize formate to CO_2_, while CO_2_ equilibrates between the aqueous/gaseous phases, carbonic acid, bicarbonate, and carbonate.

In the oxidative direction, it has been established that Fdhs use formate as the sole substrate (Figure 14). First, mass spectrometric analysis of the headspace during formate oxidation by Fdh-H shows that no oxygen atoms from the water solvent are incorporated into the CO_2_ product.^254^ Specifically, oxidation of ^13^C-labeled formate for 10 s in ^18^O-labeled water at pH 6.5 yields exclusively ^16^O-containing CO_2_. At higher pH values and with longer reaction times, increased incorporation of ^18^O into CO_2_ is observed, but this incorporation can be explained by the background hydration equilibrium between CO_2_ and HCO_3_^−^. More recent studies reported that *Rc*Fdh accelerates solvent oxygen incorporation into CO_2_,^255^ but this process is slow, and similar incorporation was also observed with catalytically inactive *Cn*Fdh.^256^ These findings indicate that the observed oxygen exchange arises from a slow postcatalytic equilibration between CO_2_ and HCO_3_^−^ (Figure 14), rather than from an oxygen atom transfer mechanism at the Mo active site.^256^ Collectively, these data support the conclusion that the sole product of formate oxidation by Fdh is CO_2_, which retains both oxygen atoms originally present in the formate molecule.^256^

### 3.2 Structure of Metal Active Sites

As shown in the Fdh crystal structures in Section 2.4, the Mo or W metal at the active site is coordinated by MGDs, a terminal sulfur ligand, and either a Cys or Sec in the primary coordination sphere (Figure 1b and Figure 15). W and Mo are both group 6 transition metals with high versatility and biological availability, and as 4d and 5d transition metals they exhibit similar ionic radii, coordination chemistries, and redox properties.^125,257^ Under physiological conditions, both W and Mo can access the IV, V, and VI oxidation states.^176^

**Figure 15:**
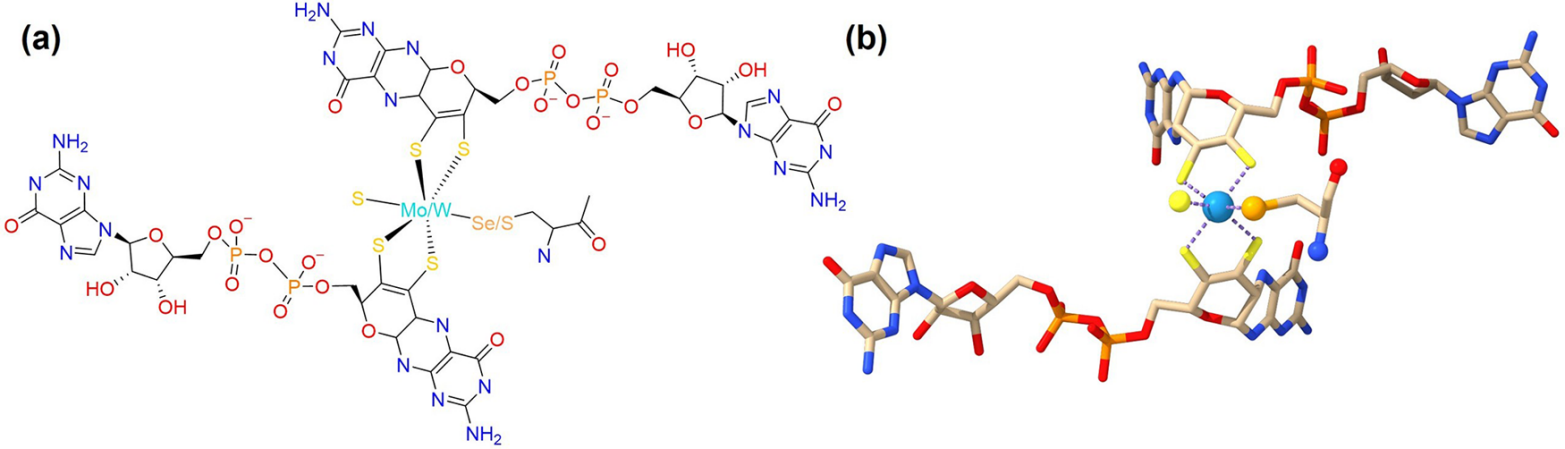
(a) Chemical structure and (b) 3D model of the metal active site in the first coordination sphere of metal-dependent Fdhs consisting of a metal center (Mo or W) bound to 2 molybdopterin guanine dinucleotides (MGDs) as well as a cysteine residue (Mo or W coordination by S) or selenocysteine residue (Mo or W coordination by Se).

In general, W-dependent enzymes show higher activity in low potential redox reactions, such as CO_2_ reduction, reflecting the lower redox potential of the W­(VI)/W­(V) and W­(V)/W­(IV) transitions compared with Mo.^246^ For example, in *Rhodobacter capsulatus* DMSO reductases, Mo could be substituted for W, and these transitions were 220 and 334 mV lower, respectively, than those of the Mo-enzymes.^258^ W-enzymes in general (e.g., aldehyde oxidoreductases, acetylene hydratases, class II benzoyl-CoA reductases, Fdhs, and FMFdhs) catalyze very low potential redox reactions (E^0^′ < −420 mV vs SHE).^246,258^ W tends to introduce a reductive bias, whereas Mo favors oxidative reactions.^246,258,259^

It is generally assumed that Mo- and W-dependent enzymes operate by cycling the metal center through the IV, V, and VI oxidation states during catalysis. Accordingly, Fdhs are proposed to cycle these redox states during both CO_2_ reduction, which initiates from the IV state, and formate oxidation, which initiates from the VI state.^176^ Although the VI and IV states are required to accomplish the two-electron redox reaction of formate and CO_2_ interconversion, only the V state in Fdhs is directly observable by electron paramagnetic resonance (EPR) spectroscopy.^130,133,191,227,254,260,261^ This observation suggests that regeneration of the active site proceeds through a one-electron pathway. Such a mechanism is consistent with the fact that the associated Fe–S clusters transfer electrons one at a time and that the Mo­(V) or W­(V) state is generated upon reduction of the enzyme by formate or other physiological electron donors. Kinetic models incorporating stepwise single-electron regeneration of the active site have successfully reproduced key features of Fdh catalytic behavior.^252,253^

An important feature of Mo- and W-dependent Fdhs is the presence of either Sec or Cys as a protein ligand coordinating the metal center (Figure 15). The group 16 elements, selenium and sulfur, share similar physicochemical properties, including electronegativity and accessible oxidation states.^262^ However, Sec is more acidic and exhibits higher nucleophilicity than Cys.^263^ The presence of Sec in redox enzymes is commonly associated with enhanced catalytic activity and increased resistance to oxidative inactivation.^262,264–266^ In addition to the Sec or Cys ligand, the first coordination sphere of the metal contains five sulfur ligands, four derived from the two MGDs and one terminal sulfur ligand (Figure 15). It is firmly established that this terminal sulfur ligand is a sulfur atom in both Mo- and W-dependent Fdhs, as well as in FMFdhs.^232,267^ This sulfur atom is essential for catalytic activity and is introduced by a dedicated chaperone protein.^268–270^ Chaperone proteins assist in the correct folding, assembly, and transport of other proteins within a cell, while also preventing misfolding and aggregation, without becoming a permanent part of the final functional structure.

The MGD ligands in the active site of Fdhs are not thought to participate directly in substrate binding. However, they likely play a role in mediating electron exchange with the metal center throughout the catalytic cycle. Multiple EPR studies of the Mo­(V) state have reported signals with g values near 2.094, close to that of a free electron.^36,133,217,254,260,271^ These observations suggest delocalization of an electron onto the pterin ligands, consistent with their redox sensitivity and with the known electrochemical behavior of synthetic Mo-dithiolene complexes.^272^ The MGD ligands are therefore considered redox noninnocent and are proposed to interconvert between dihydro and tetrahydro states through a coupled two-electron, two-proton process.^273–279^

The metal active site of Fdhs is surrounded by conserved His and Arg residues in the second coordination sphere, which are thought to contribute to proton transfer and substrate binding. For example, crystallographic studies of *Nv*FdhAB show redox dependent rearrangements of the protein backbone and side chains,^51,280,281^ most notably involving His residue,^282^ suggesting a role in binding substrates or inhibitors. This is supported by the pH dependence of azide binding to the Mo­(VI) state, which indicates involvement of a residue with a p*K*_a_ around 6.5, consistent with His.^252^ In *Rc*Fdh, mutation of a conserved His (H387M) lowers affinity for azide and cyanate by about an order of magnitude, further implicating His in ligand binding.^283^ These redox linked conformational changes, including His side chain reorientation, likely underlie reductive activation of Fdhs,^49,51,191,216,252,284^ rather than dissociation of the Sec or Cys ligand.^252,285,286^ Conserved Arg residues are also present in all Fdh active sites, including metal independent enzymes, and are likewise proposed to participate in proton transfer and substrate binding.^54,103,283,287^

### 3.3 Consensus Mechanism for Fdh Catalysis

In the CO_2_ reduction direction, Fdhs are widely thought to operate via formal hydride transfer rather than proton transfer (Figure 16). Protonation of CO_2_ is thermodynamically unfavorable because the effective p*K*a of the carbon atom is extremely low, making involvement of biological acids unrealistic, even within a protein environment.^130,216,249^ Instead, the reduced active site, commonly presented in the protonated form as Mo­(IV)-SH or W­(IV)-SH, acts as a hydride donor to CO_2_, reducing it to formate in the second coordination sphere without direct substrate binding to the metal. This hydride transfer mechanism is consistent with formate formation in both metal-dependent and metal-independent Fdhs.^106,116^

**Figure 16:**
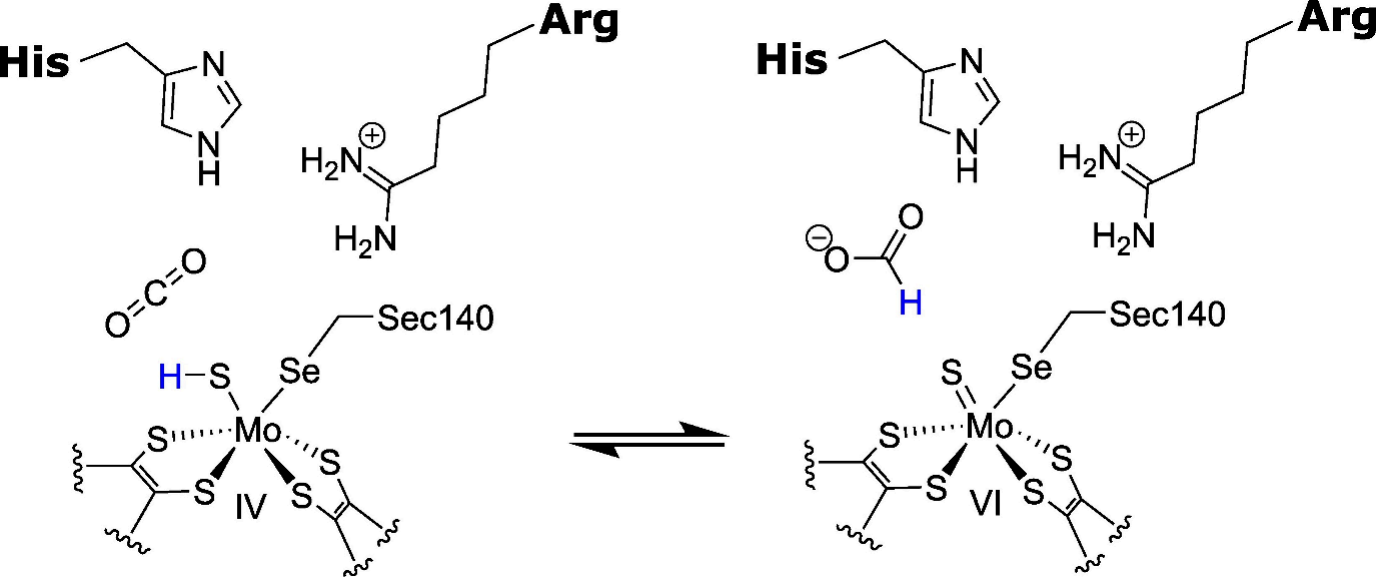
Consensus mechanism for Fdh catalysis showing CO_2_, formate, His, and Arg in the second coordination sphere.^130,216^

In the reverse reaction, formate oxidation likewise proceeds through hydride transfer (Figure 16). Formate binds in the second coordination sphere and donates a hydride to the terminal sulfur ligand in the metal active site, Mo­(VI)=S or W­(VI)=S.^106,130,216^ EPR studies show strong coupling between the C_α_ hydrogen of formate and the metal center, indicating close proximity of the transferred hydrogen to the first coordination sphere via the terminal sulfur ligand.^130,217,254^ Multiple spectroscopic and kinetic studies support Mo-SH or W-SH as a key intermediate in both reaction directions, analogous to hydride transfer intermediates proposed for other Mo-dependent enzymes such as xanthine oxidases.^176^

Nevertheless, several mechanisms have been previously proposed for Fdh catalysis.^50,54,252,267,285,286,288^ A central question has been whether the Sec or Cys ligand dissociates from the metal center during turnover or remains bound throughout the catalytic cycle, in both the CO_2_ reduction and formate oxidation directions. Current evidence supports a mechanism in which the Sec or Cys ligand remains coordinated to the metal, and CO_2_ is reduced to formate in the second coordination sphere, without direct binding of the substrate to the metal center (Figure 16).^106,130,216,248,249,252,269,285–287,289^

Multiple lines of evidence support continued coordination of the Sec or Cys ligand to the metal center throughout Fdh catalysis. Early crystallographic analysis of oxidized Fdh-H showed Sec coordinated to Mo,^50^ a feature later confirmed in structures of several other Fdhs, including Fdh-N,^132^ *Nv*FdhAB,^51,280–282^ and *Mg*FdhAB.^190^ Importantly, the original Fdh-H structure is essentially unchanged from its reduced form, indicating stable metal coordination during turnover.^50^ This conclusion is reinforced by numerous recent structures of *Nv*FdhAB, including reduced-state and time-resolved crystallographic studies, which consistently show Sec remaining bound to the metal during reduction and support a second coordination sphere catalytic mechanism.^51,214,239,280,281,284^

Spectroscopic and electrochemical data further corroborate this view. EPR studies of Fdh-H^254,260^ and *Nv*FdhAB^284^ detected strong ^77^Se coupling to the Mo­(V) or W­(V) state, providing strong evidence that the Sec ligand remains coordinated to the metal during catalysis. Consistent with these findings, X-ray absorption spectroscopy (XAS) of oxidized and dithionite-reduced forms of Fdh-H shows that both states have very similar metal coordination environments.^290^ Comparable results for oxidized and reduced *D. desulfuricans* Fdh (*Dd*Fdh)^291^ and *Rc*Fdh^269^ also indicate little change in coordination environment around Mo upon dithionite reduction. Electrochemical studies across multiple Fdhs likewise support formate binding outside the first coordination sphere.^292^ In contrast, crystallographic observations of Sec dissociation in *Nv*FdhAB arise only under oxidative damage conditions, where O_2_ or peroxide replaces Sec at the metal center, leading to irreversible enzyme inactivation.^281^ These inactive species indicate that Sec dissociation reflects oxidative damage rather than a physiologically relevant catalytic state.

### 3.4 Inhibition of the Fdh Metal Active Site

The Fdh metal active site can bind a range of small molecules,^187,251,265,293,294^ mainly anionic ions, such as azide,^51,217,252,283,288^ nitrite,^50,252^ nitrate,^51,252^ cyanide^216,217,288^ and derivatives such as cyanate^283^ and thiocyanate,^252^ which are isoelectronic and isostructural with CO_2_ or formate. These species act as inhibitors and have been widely used as probes of Fdh catalytic function. Most inhibitors display competitive inhibition with respect to formate oxidation, with inhibitory strength correlating with their electron-donating ability (azide > cyanate > thiocyanate > nitrite > nitrate). In contrast, CO_2_ reduction generally shows apparently noncompetitive inhibition, except for nitrite. For all inhibitors, the degree of inhibition depends on their redox potentials, indicating that inhibitor binding at the Fdh active site is redox dependent.^252^

Metal-independent Fdhs are also inhibited by small molecules such as azide^295^ and iodoacetamide,^103,296^ indicating that inhibitor binding in metal-dependent Fdhs need not involve direct coordination to the Mo center, although similar substrate scopes likely lead to comparable small-ion affinities. Consistent with this view, IR and DFT studies suggest that azide and cyanate bind in the second coordination sphere of *Rc*Fdh rather than directly to the Mo metal center.^283^ In addition, crystallographic analysis of the dithionite-reduced U192C mutant of *Nv*FdhAB has been interpreted as showing a sulfur dioxide (SO_2_) molecule bound at a Cys-associated active site.^284^ In this structure, SO_2_ is stabilized by hydrogen bonding to an active site Arg residue and may weakly interact with the Cys sulfur (Figure 17).

**Figure 17:**
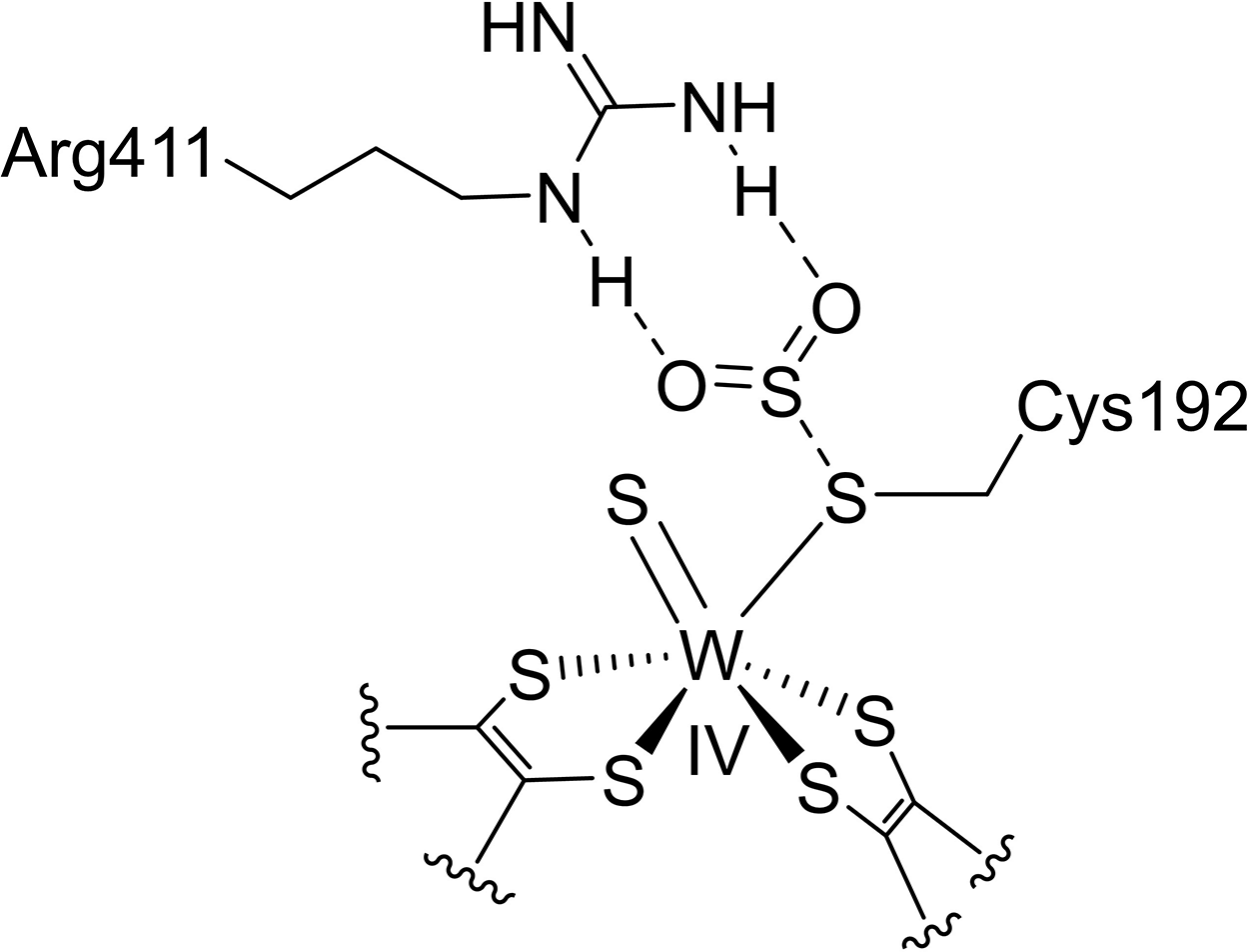
SO_2_ binding to the W­(IV) active site, based on reported crystal structure modeling and analogous calculated intermediates.^284,287^

Iodoacetamide tagging experiments have been reported for many Fdh types and typically monitor enzyme inactivation during formate oxidation.^51,54,103,265^ Early studies on Fdh-H showed that iodoacetamide inactivation is more common at higher pH,^265^ with the Cys variant requiring higher pH than the native Sec enzyme.^265^ A similar increase in iodoacetamide sensitivity with pH has been observed for Cys-containing *Rc*Fdh.^54^ Inactivation at pH 6 requires the presence of formate for both Sec and Cys variants,^265^ and nitrate promotes inactivation over a pH range of 6 to 10.^54^ In *Rc*Fdh, the H387M mutant exhibits faster iodoacetamide inactivation than the wild type, consistent with loss of a His residue that elevates the p*K*a of the neighboring Cys, thereby increasing its nucleophilicity.^54^

These trends led to the proposal that iodoacetamide inactivation arises from alkylation of the Sec or Cys ligands in the first coordination sphere, with the lower p*K*_a_ of Sec enabling formation of Se^–^ at lower pH than S^–^.^297^ However, mass spectrometric analysis of iodoacetamide-treated *Nv*FdhAB revealed alkylation of multiple Cys residues, including those coordinating Fe–S clusters and surface Cys, while Sec remained unmodified.^51^ Moreover, iodoacetamide alkylation can occur without loss of metal coordination.^298^ Collectively, these results indicate that iodoacetamide labeling is unselective and therefore an unreliable probe for assessing the specific role of the active site Sec or Cys residue.

## 4 Fdh *in* *Vitro*

This section focuses on the *in vitro* applications of Fdhs, with particular emphasis on properties relevant to biotechnological deployment. We cover strategies for enzyme isolation and purification, robustness and scalability under operational conditions, and distinctive catalytic features that differentiate Fdhs from other CO_2_-converting enzymes. The discussion also addresses oxygen tolerance and stability outside the cellular environment, as well as how *in vitro* studies have enabled detailed mechanistic insights into catalytic activity.

### 4.1 Isolation and Purification

The first step of *in vitro* application of Fdhs is the isolation of the target enzyme from a mixture of other enzymes and components in the source organism. These separation and purification procedures must not only retain the biological activity and chemical integrity of the enzyme, but also effectively remove contaminants (e.g., other proteins, nucleic acids, viruses, cell culture media) as well as other isoforms (i.e., closely related versions of the same protein).^299^ Enzymes can either be isolated in the native form directly from the natural source organism (native purification) or expressed in the recombinant form, where genetic engineering is used to produce the target enzyme in a host cell (e.g., bacteria or yeast).

The native purification of Fdh was first reported for the cytoplasmic Fdh-H from *E. coli*.^187^ Two column chromatographic steps were employed, the first of which leveraged on the hydrophobic character of Fdh-H to adhere the enzymes to a phenyl Sepharose column, whereas the second step utilized hydroxylapatite to separate Fdh-H from bulk contaminants. The nearly homogeneously purified Fdh-H had a light, yellow-brown color and was determined by electrophoresis to have a molecular weight of 80 kDa. Adapting from these procedures, the same enzyme was also purified using a Q-Sepharose column (anion-exchange column) in the second step with an additional third step involving a gel filtration column based on size exclusion chromatography.^49^

While native purification preserves the original structure of the natural enzyme without surface modification, such enzyme production can be complex and time-consuming, as well as limited in supply and scalability. Alternatively, enzymes expressed in the recombinant form can be purified by affinity purification, which is faster, and achieves higher yield and activity of the isolated enzymes.^300^ Recombinant expression involves introducing a cloned gene encoding the target enzyme into a host organism for enzyme production, where the host organism can either be identical or relative to the native source organism, known as homologous or heterologous expression, respectively. Furthermore, recombinant expression allows target enzymes to be modified with specific affinity tags such as Histidine tag (His-tag) or streptavidin tag (Strep-tag) to enhance selective binding to Ni^2+^ or biotin, respectively. These tags are short protein sequences or peptides that have highly specific binding partners. Affinity purification (or affinity chromatography) can then be used,^301^ leveraging on these specific binding interactions to isolate the target enzyme in a facile, one-step procedure with very high purity.^302,303^

The first heterologous expression of a metal containing Fdh was executed for *Rc*Fdh.^129^ The His-tagged *Rc*Fdh was expressed in *E. coli* and purified with a Ni^2+^–nitrilotriacetic acid resin and size exclusion chromatography, resulting in the isolation of enzyme in high purity (90–95%).^129^ Strep-tagged *Cn*Fdh expressed in *E. coli* was similarly purified with a Strep column, followed by an ion exchange and ammonium sulfate precipitation to obtain the active enzyme.^304^ It should be noted that the expression of W-dependent enzymes in *E. coli* has been reported, which is considered an important challenge in the field.^305^

Homologous expression of Strep-tagged *Nv*FdhAB,^51,214^ and FdhM from the same organism,^193^ also yielded high purity enzymes in a one-step affinity purification. Remarkably, the recombinant *Nv*FdhAB could be purified and handled under aerobic conditions with nitrate and glycerol as stabilizing agents, wherein the nitrate acts as an inhibitor that protects the enzyme likely by preventing the loss of the labile sulfido group.^51,306^

Recombinant expression systems are also advantageous in that protein variants can be generated to investigate several features, including the catalytic mechanism. The first variant produced was the Sec-to-Cys variant of the Cys-FhL from *E. coli* which significantly affected formate oxidation activity,^265^ as well as the CO_2_ reducing activity.^307^ The same mutation in the *Nv*FdhAB enzyme confirmed the strong impact on activity, and revealed that the Sec residue is also important to increase O_2_ tolerance and may function in H^+^ transfer to/from the active site.^284^ O_2_ tolerance of *Nv*FdhAB was also further investigated with the mutation of a surface disulfide bond which revealed the presence of a redox switch mechanism for protection against transient O_2_ exposure (see Section 4.3).^214,282^ Meanwhile, variants of active site residues of the *Rc*Fdh confirmed the important role of the conserved His and Arg residues in catalysis.^54^

### 4.2 Fdh Catalysis *in Vitro*

The catalytic activity of Fdh toward CO_2_ reduction or formate oxidation can be quantified in a solution assay by measuring the initial reaction velocity (ν_0_) at different substrate (CO_2_ or formate) concentrations, in the presence of electron donors or acceptors, respectively. Typically, a redox mediator such as methyl viologen or benzyl viologen with a characteristic absorption maximum is employed. By monitoring the change in absorbance at the characteristic wavelength of the redox mediator upon charge transfer, ν_0_ for CO_2_ reduction or formate oxidation can be found (Figure 18). Thereafter, by employing the Michaelis–Menten model (eq 3), where *V*_max_ is the maximum reactions rate reached at a saturated substrate concentration and [S] is the substrate concentration, key kinetic parameters such as the Michaelis constant (*K*_M_), turnover number (*k*_cat_, it can also be denoted as TON but is typically represented as *k*_cat_ in enzyme catalysis) and the catalytic efficiency (*k*_cat_/*K*_M_) can be found. The definition of these properties and their derivation is detailed in other reviews.^308,309^

3 $$v_{0}=\frac{V_{\max}\left[S\right]}{K_{M}+\left[S\right]}$$

The kinetic properties of the best characterized metal-dependent Fdhs, for which both formate oxidation and CO_2_ reduction are reported, are summarized in Table 3.

**Figure 18:**
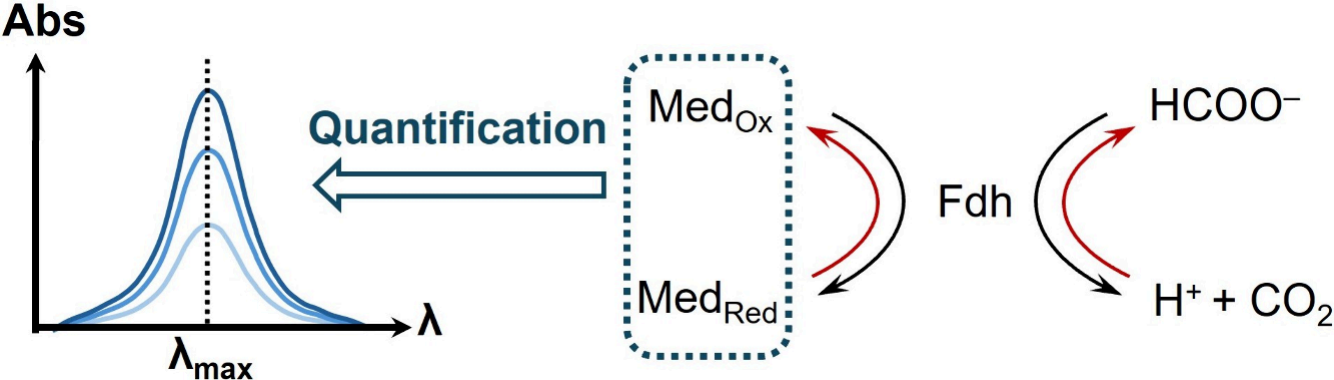
Brief concept diagram of solution assay measurements for quantifying Fdh activity toward formate oxidation and CO_2_ reduction via the use of a redox mediator with a characteristic absorption maximum.

**Table 3:** Kinetic Properties of Some Metal Dependent Fdhs for Both Formate Oxidation and CO_2_ Reduction^a^

		*k*_cat_ (s^–1^)			*K*_M_ (μM)			*k*_cat_/*K*_M_ (s^–1^ mM^–1^)		
Organism	Enzyme	e^–^ acceptor/donor	Formate Oxidation	CO_2_ Reduction	e^–^ acceptor/donor	Formate	CO_2_	Formate Oxidation	CO_2_Reduction	ref
***E. coli***	Fdh-H	BV^2+^/MV^+^	160	<1	graphite-epoxy electrode	800	2500	200	<1	^49^,^252^
***N. vulgaris*** **Hildenborough**	FdhAB	BV^2+^/MV^+^	1310	345	BV^2+^/MV^+^	17	324	77,515	1 090	^214^
***S. fumaroxidans***	Fdh1	BV^2+^/MV^+^	1900	2460	BV^2+^/MV^+^	40	^a^	47,500	^a^	^61^,^128^
		MV^2+^/MV^+^	3380	280	^a^	^a^	^a^	^a^	^a^	
	Fdh2	BV^2+^/MV^+^	5600	185	BV^2+^/MV^+^	10	^a^	560,000	^a^	^128^
***C. necator***	FdsDABG	NAD^+^/NADH	200	11	NAD^+^/NADH	310	2700	648	4	^130^,^175^
***R. capsulatus***	FdsDABG	NAD^+^/NADH	37	2	NAD^+^/NADH	280	^a^	132	^a^	^129^
***A. woodii***	FdhF	MV^2+^/MV^+^	1690	372	MV^2+^/MV^+^	1000	37,000	1690	10	^131^
***T. kivui***	FdhF	MV^2+^/MV^+^	1355	HDCR complex:	MV^2+^/MV^+^	550	^a^	2464	^a^	^182^
				3228 (70 °C)						
				2660 (60 °C)						
				515 (30 °C)						

a Not reported.

In general, the activity for formate oxidation is higher than for CO_2_ reduction, with two notable known exceptions. For the *S. fumaroxidans* Sec-W-Fdh1, the *k*_cat_ toward CO_2_ reduction measured using dithionite-reduced methyl viologen as electron donor was ∼2460 s^–1^ at pH 7.3, higher than that for formate oxidation (∼1900 s^–1^ at pH 8) measured using benzyl viologen as electron acceptor.^128^ However, a later study reported a lower turnover rate toward CO_2_ reduction (282 s^–1^ at pH 7.5) than toward formate oxidation activity (3380 s^–1^ at pH 8) for the same enzyme.^61^ Despite the high catalytic activity, this enzyme is extremely susceptible to O_2_ damage.

The second exception is the fastest enzyme in CO_2_ reduction reported so far, the *Tk*HDCR, which has a *k*_cat_ of 515 s^–1^ at 30 °C, but higher at 70 °C (3228 s^–1^) as expected for a thermophilic enzyme.^182,310^ However, the *Tk*HDCR enzyme is also very sensitive to O_2_, requiring strictly anaerobic conditions and the presence of dithiothreitol (DTT) in the activity assays.^182^ In contrast, the *Nv*FdhAB is an interesting enzyme for practical applications, since it couples one of the highest CO_2_ reduction rates under mild conditions (315 s^–1^) with the ability to be handled aerobically.^51^ Furthermore, a homologous recombinant expression system is in place that facilitates its production and purification.^51^

Note that Fdh catalysis involves both chemical steps at the active site and subsequent intraprotein electron transfer steps, and the relative rates of these processes strongly influence the observed enzyme behavior. Activity-based electrochemistry, solution assays, and presteady state kinetics have shown that electron transfer can be rate limiting during formate oxidation by Fdh-H.^253^ When interfacial electron transfer is slow, key catalytic parameters can be masked, leading to apparent values of *V*_max_, *K*_M_, and kinetic isotope effects (KIEs) that do not directly reflect the intrinsic catalytic rates at the active site. For example, solution assays of formate oxidation using benzyl viologen as the electron acceptor yielded a KIE on *V*_max_ (V_max_^H^/V_max_^D^) of approximately 1, whereas electrochemical measurements (see Section 5.1) gave a value of 2.44 at 0 V vs SHE, and presteady state stopped-flow experiments reported a KIE of about 3.2.^253^ The apparent *K*_M_ values for formate were also isotope dependent and method dependent, being much lower in solution assays (a *K*_M_^H^ of 58 μM and a *K*_M_^D^ of 158 μM) than in electrochemical measurements (a *K*_M_^H^ of 790 μM).

These seemingly contradictory results can be reconciled using a unified kinetic scheme that contains separate steps for substrate binding, chemical reduction/oxidation, and the electron/proton transfer required to regenerate the active site (Figure 19). By fitting this model, with partially shared parameters, simultaneously to solution assay, electrochemical, and stopped-flow data, it was shown that the different experimental methods probe different rate-limiting steps.^253^ As a result, variations in electron transfer rates, rather than changes in the intrinsic active-site chemistry, account for the observed differences in *V*_max_, *K*_M_, and KIE across techniques.

**Figure 19:**
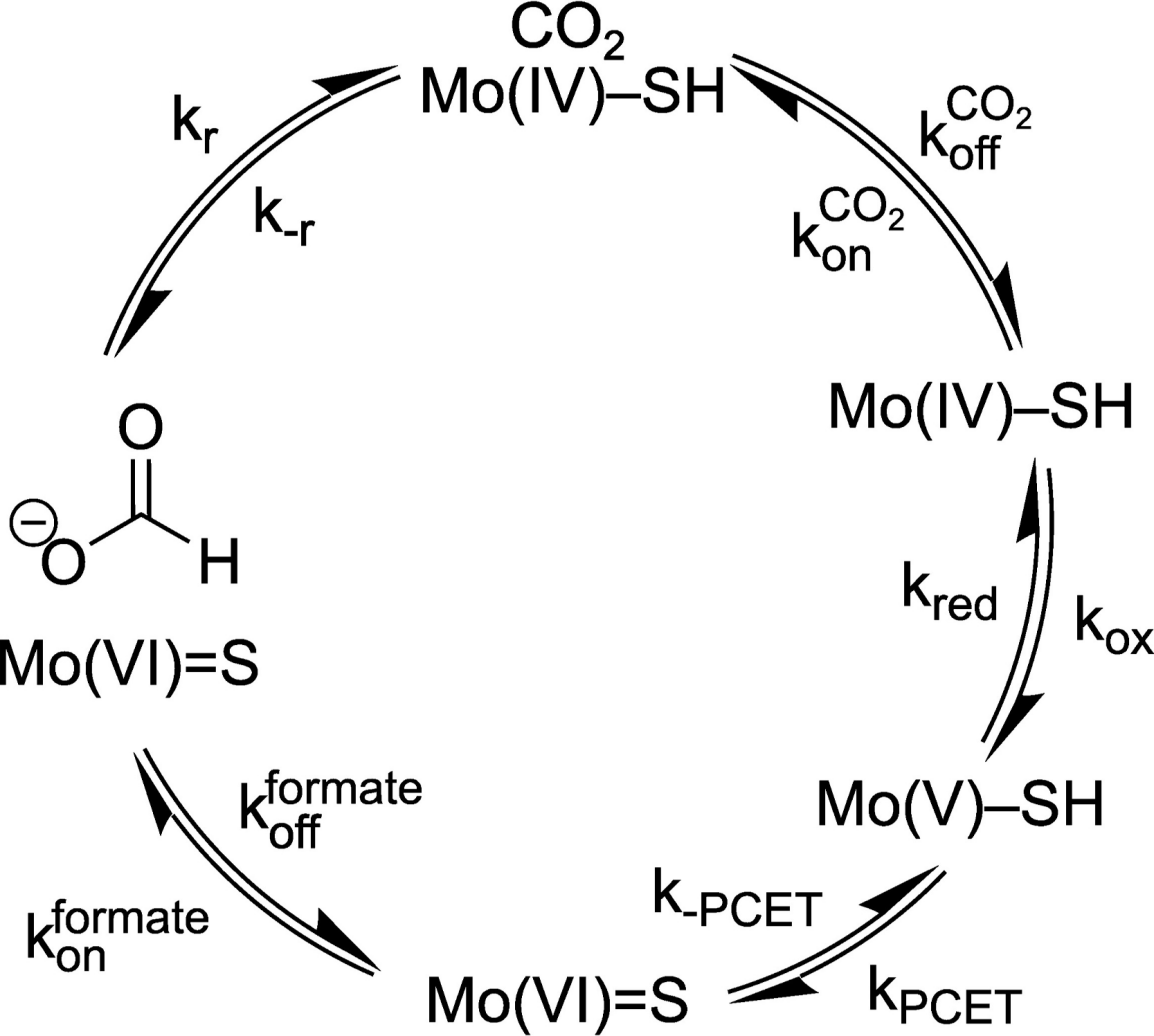
A kinetic scheme for Fdhs which incorporates substrate binding, active site turnover, and electron transfer from the active site to regenerate it.^253^ This scheme is agnostic to the precise mechanism of the interconversion between CO_2_ and formate at the active site. PCET: Proton Coupled Electron Transfer.

### 4.3 Oxygen Tolerance

Metal-dependent Fdhs display high turnover rates for CO_2_ reduction with little energy loss, but most face challenges of oxygen sensitivity, which is more acute for the most active enzymes, containing W and Sec.^61,128,182^ In fact, the majority of both Mo- or W-dependent Fdhs are quickly inhibited by O_2_ and need to be handled anaerobically. Examples are Fdh-H from *E. coli*,^187^ and Fdh1 and Fdh2 from *S. fumaroxidans*,^128^ which lose activity irreversibly after short durations of oxygen exposure.^128,187^ The Mo-containing NAD^+^-dependent enzymes from *R. capsulatus* ^129^ and from *C. necator* ^175,228^ are considered oxygen-tolerant enzymes and their NAD^+^-mediated formate oxidation activity can be measured aerobically, although the activity declines gradually especially if a stabilizing agent (e.g., nitrate) is not present.^311^ As the presence and binding of formate results in oxygen sensitivity of Fdh, stabilizing agents that competitively inhibit the binding of formate can enable Fdh to be handled or stored aerobically.

The FdhM enzyme from *Nv*H has been purified aerobically in the absence of any stabilizing agent.^193^ This enzyme is also capable of aerobic formate oxidation with high-potential electron acceptors or with O_2_ itself, which is reduced to H_2_O_2_ without apparently affecting the active site, but is not reported to reduce CO_2_.^193^ For all Fdhs, CO_2_ reduction in the presence of O_2_ is difficult due to the low reduction potentials involved in the reaction, since the reductants used can react directly with O_2_.

While most Fdhs require anoxic conditions and a reductive activation step for catalysis, some Fdhs can be purified and handled aerobically. A striking example is the *Nv*FdhAB, which would be expected to be extremely O_2_-sensitive, similar to other tungstoenzymes and W-complexes mimicking their active site,^246,258,259^ but it can be purified and handled aerobically.^51,239^ The mechanism behind this was recently described and involves an allosteric disulfide bond at the enzyme surface that controls the activity and O_2_ stability of the enzyme (Figure 20).^214^ When the disulfide bond is present (resting state) the enzyme displays negligible activity and presents a very high *K*_M_ for formate (2.5 mM) that precludes its reduction *in vivo*, and keeps it stable to O_2_. Upon reduction of the disulfide bond the enzyme is converted to the fully active conformation, which involves structural changes that propagate from the surface to the active site. In this state, the enzyme is more sensitive to O_2_ and can be readily reduced by physiological formate concentrations (*K*_M_ for formate 17 μM) to the O_2_-sensitive W­(IV)-SH state.^214^ This mechanism allows protection of FdhAB when *Nv*H is transiently exposed to O_2_, as it happens in its natural habitats.

**Figure 20:**
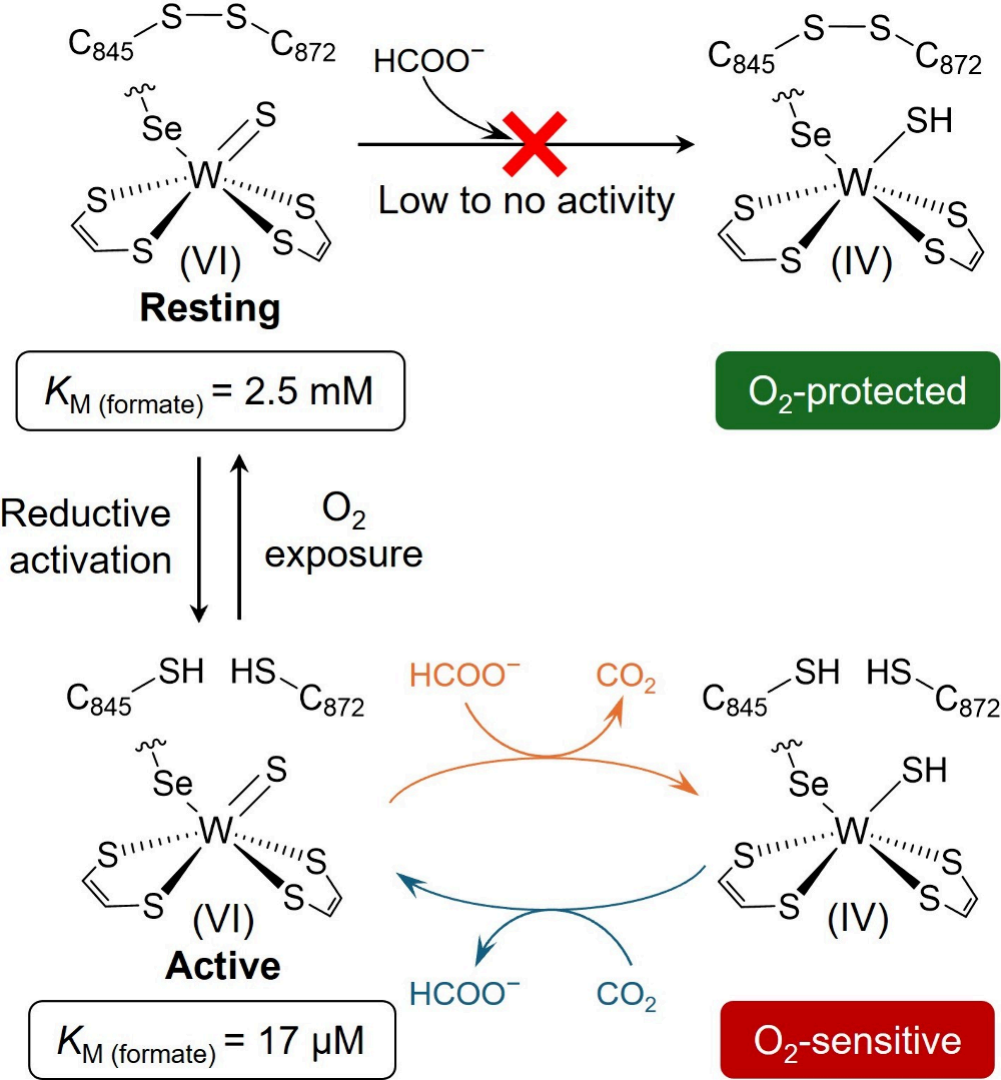
Schematic highlighting the different physiological states in the *Nv*FdhAB cycle and how the reductive activation of the disulfide bond affects the activation and oxygen sensitivity of the enzyme.^214^

Another Fdh that displayed remarkable oxygen tolerance is the *So*FdhAB which, unlike *Nv*FdhAB, maintains its activity before and after treatment with the reducing agent, DTT.^194^ Cryo-EM analysis of *So*FdhAB revealed a different substrate channel from *Nv*FdhAB, wherein the exit of the gas substrate tunnel was controlled by the residues H653 and V666. More crucially, the bulky residue Y776 which blocks the tunnel and another residue T927 on the opposite side of the channel, play a vital role in oxygen protection, evidenced by the difference in activities for the constructed *So*FdhAB-Y776A/T927A variants under aerobic and anaerobic conditions. NAD-dependent Fdh from *Clostridium carboxidivorans* (*Cc*Fdh) was also highlighted for its ability to reduce CO_2_ to formate under aerobic conditions.^244^ Coupled with the ability to use NAD­(P)H as an electron source, *cc*FDH was found to be suitable for supporting CO_2_ fixation via the Calvin–Benson–Bassham (CBB) cycle in oxygenic phototrophs.^312^

Several possible mechanisms have been identified in the oxidative inactivation of Fdhs. The most general one involves the loss of the essential sulfur ligand, which can occur faster in the reduced W­(IV)-SH or Mo­(IV)-SH state than in the oxidized W­(VI) or Mo­(VI) state and at higher pHs,^269,311^ involving exchange with hydroxide. In the absence of a stabilizing agent, the reduced *Cn*Fdh was inactivated within minutes of O_2_ exposure.^304^ Upon contact with O_2_, the reduced enzyme was shown to produce superoxide that reacts with the terminal sulfur ligand to form sulfite leading to enzyme inactivation, which could have been prevented by the presence of superoxide dismutase and catalase.^304^ This process also explains why inhibitors like nitrate can stabilize the enzyme by sterically preventing access to the terminal sulfur ligand.^306^ Partial loss of this ligand was also observed in the aerobically obtained structure of the active C872A variant of *Nv*FdhAB.^214^

However, an additional mechanism for oxidative inactivation was recently described for this enzyme. Although *Nv*FdhAB could perform formate oxidation with O_2_ as electron acceptor and does not suffer oxidative damage for short periods of O_2_ contact (<12 min), structural studies revealed that upon prolonged O_2_ exposure in the presence of either substrate (formate or CO_2_), dissociation of the Sec ligand occurs, which is displaced by a dioxygen or peroxide molecule, leading to irreversible inactivation.^281^ Remarkably, this was also observed for the enzyme in the presence of CO_2_, where the metal is not reduced. Although a small loss of the terminal sulfur ligand was also observed, addition of superoxide dismutase and catalase did not offer significant protection, indicating that the main mechanism involved in inactivation was the displacement of the Sec ligand from W coordination. These results suggest that binding of either substrate induces a conformational state that is more susceptible to O_2_ attack and Sec displacement,^281^ and suggest that previous reports of Sec dissociation in Fdh-H^267^ and Cys dissociation in *R. capsulatus* Fdh, accompanied with oxygen ligation,^288^ are probably associated with inactivated forms.

## 5 Characterizations at the Fdh–Abiotic Interface

Characterizing Fdh–abiotic interactions is crucial to achieve and optimize DET through controlling enzyme orientation, surface modifications, and overall system efficiency.^313–315^ This section introduces a complementary set of interfacial characterization techniques and analysis that enable real-time monitoring of Fdh adsorption and desorption, structural integrity, as well as substrate/inhibitor binding. These techniques provide invaluable fundamental insights into Fdh photo­(electro)­catalysis, facilitating the rational design and development of efficient and robust semiartificial photosynthesis. The electrochemistry section focuses on the use of Fdh as a model electrocatalyst for the electrochemical interconversion of CO_2_ and formate. This will cover the unique advantages of metal-dependent Fdh compared to synthetic electrocatalysts in terms of overpotential, reversibility, and tunability. It will also highlight the key applications of Fdh for CO_2_ reduction in electrocatalysis to produce chemicals and for formate oxidation in biofuel cells to generate electricity.

### 5.1 Fdh Electrochemistry

Metal-dependent Fdhs, featuring Mo or W cofactors, contain Fe–S clusters that efficiently shuttle electrons across the insulating protein scaffold to the buried metal active site (Figure 21a). These Fdhs can also be adsorbed onto an electrode to accept or donate electrons via DET, enabling the activity of these enzymes to be probed directly with electrochemical techniques such as protein film voltammetry (PFV) or more generally, protein film electrochemistry (PFE).^313,316–319^

**Figure 21:**
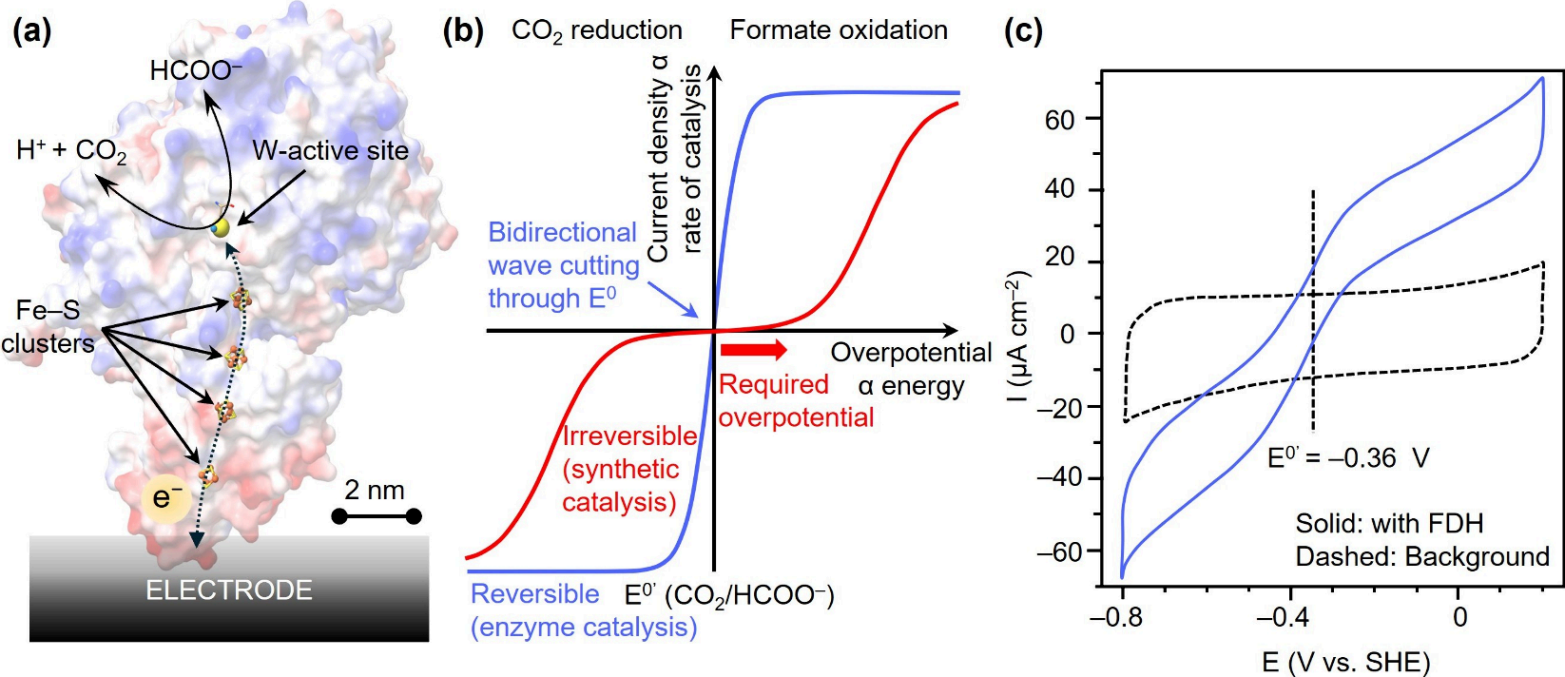
(a) Structure of W-dependent *Nv*FdhAB (PDB: 6SDV)^51^ adsorbed onto an electrode, highlighting the reversible electron transfer to and from the W-active site through the [Fe–S] clusters.^61^ (b) Cyclic voltammograms of ideal irreversible and reversible electrochemical reactions. Numerical data adapted from ref 318 with permission from National Academy of Sciences, copyright 2011. (c) Protein film voltammetry of Fdh from *S. fumaroxidans* adsorbed onto polished pyrolytic graphite edge electrode in 10 mM CO_2_ and 10 mM sodium formate (pH 6.4) showcasing the interconversion of CO_2_ and formate at the reduction potential (black dashed line). The background current is represented by the dashed line. The conditions employed were 100 mV s^–1^ scan rate, 37 °C and 1000 rpm electrode rotation. Numerical data adapted from ref 61 with permission. Copyright 2008 National Academy of Sciences.

Electrochemical reversibility refers to when the kinetics of interfacial electron transfer is sufficiently fast to maintain Nernstian equilibrium at an electrode surface, wherein a small amount of additional energy input beyond the thermodynamic requirements, known as overpotential, produces a significant catalytic current that reflects the shifting Nernstian equilibrium.^318,320–322^ In an ideal scenario (Figure 21b),^318^ a reversible reaction gives rise to a single sigmoidal curve (blue) that intercepts through zero current at the equilibrium potential (E^0^’) and reaches a limiting current independent of applied potential in both directions. The magnitude of the equivalent oxidation and reduction currents at E^0^’ is known as the exchange current, which is high in reversible systems.^323^ Meanwhile, irreversible systems display low exchange currents and have negligible response to potential changes near E^0^’.^323^ A significant overpotential is required before current increases, reflected in the two waves (red) increasing exponentially with potential, each in one direction. Consequently, a significant overpotential is required to match the current produced in the reversible system, which results in a loss in energy and selectivity.

In the case of an enzyme-modified electrode, electrochemical reversibility is better described with electrocatalytic exchange currents, which encompasses the interfacial electron transfer as well as the enzyme’s turnover and intramolecular electron transfer, each having the ability to limit electrocatalysis.^318^ In this regard, metal-dependent Fdhs are reversible electrocatalysts that showcases high electrocatalytic exchange currents and correspondingly, very minimal overpotential requirement in either direction. Metal-dependent Fdhs are capable of performing the reversible interconversion of CO_2_ and formate and hence, are suitable model electrocatalysts for probing CO_2_ reduction or formate oxidation reactions.

A polished pyrolytic graphite edge (PGE) electrode adsorbed with W-dependent Fdh from *Syntrophobacter fumaroxidans* demonstrated an onset potential for CO_2_ reduction and formate oxidation at approximately −0.4 V vs SHE (Figure 21c), close to the thermodynamic potential of −0.36 V vs SHE (pH 6.5).^61^ Notably, the PFV showcased a sigmoidal onset reflecting the high electrocatalytic exchange currents characteristic to a reversible system. This onset is followed by a linear relationship as the overpotential is increased which highlights that even at the highest driving force applied, the rate of IET is still slower than the rapid active-site turnover, hence the limiting factor to electrocatalysis.

A subsequent study^49^ also demonstrated the reversible interconversion of CO_2_ and formate using Mo-containing Fdh-H from *E. coli* adsorbed onto a graphite-epoxy rotating disk and similarly concluded that the electrocatalytic rate of the enzyme is limited by interfacial electron transfer. Nonetheless, the catalysis remained reversible across a variety of conditions (pH, applied E vs SHE), following the Pourbaix diagram in Figure 1a. Controlled potential electrolysis with the Fdh-H adsorbed on the electrode at applied potentials of −0.5 and −0.6 V vs SHE (pH 6.9) resulted in the quantitative conversion of CO_2_ to the single product formate with a Faradaic efficiency (FE) of 102 ± 2%.^49^

The reversible nature and CO_2_ reduction activity of W-dependent *Nv*FdhAB was also established,^324^ demonstrating the interfacial electron transfer from a metal oxide-based cathode to the Fe–S clusters of Fdh and an onset potential (−0.36 V vs SHE, pH 6.5) close to the thermodynamic potential.^325^ The Fdh cathode achieved a current density of −240 μΑ cm^–2^ at −0.6 V vs SHE and a FE of around 78% toward formate production.

Similar DET-type bioelectrocatalysis was achieved with the engineered *Tk*HDCR.^326^ The study delved into the heterologous expression of *Tk*HDCR in *E. coli* and subsequently the truncation of *Tk*HDCR to obtain Fdh variants displaying catalytic activity toward both the reduction of CO_2_ and formate oxidation. Formate oxidation through a DET mechanism was also studied with the immobilization of *Bs*Fdh on PGE rotating-disc electrodes.^292^ A model that describes the cycling of the enzyme’s active site between three redox states (IV, V, and VI) was fitted to baseline-subtracted voltammograms, enabling deeper understanding of how substrate binding and deprotonation are coupled to electron-transfer steps during catalytic formate oxidation.

Benefitting from the electrochemical reversibility of metal-dependent Fdh, these enzymes have been employed for formate oxidation in biofuel cells,^327,328^ as well as in CO_2_ reduction for the sustainable production of formate.^55^ With the early reports of high turnover numbers (112 s^–1^) attained and the low overpotential (∼50 mV) required for CO_2_ reduction,^61^ metal-dependent Fdh serve as a model electrocatalyst with catalytic performance superior to state-of-the-art synthetic molecular catalysts (e.g., Fe-porphyrins) or heterogeneous metal catalysts (e.g., Au, Pb, Bi and Sn), hence paving the rational design of synthetic CO_2_ reduction electrocatalysts.^55,289,329–332^ Moreover, the high catalytic activity of metal-dependent Fdhs and their ability to perform DET spurred the development of electrodes over the years (Figure 22), to achieve stable immobilization of enzymes, to increase enzyme loading and to improve the interfacial electron transfer between electrodes and Fdh.

**Figure 22:**
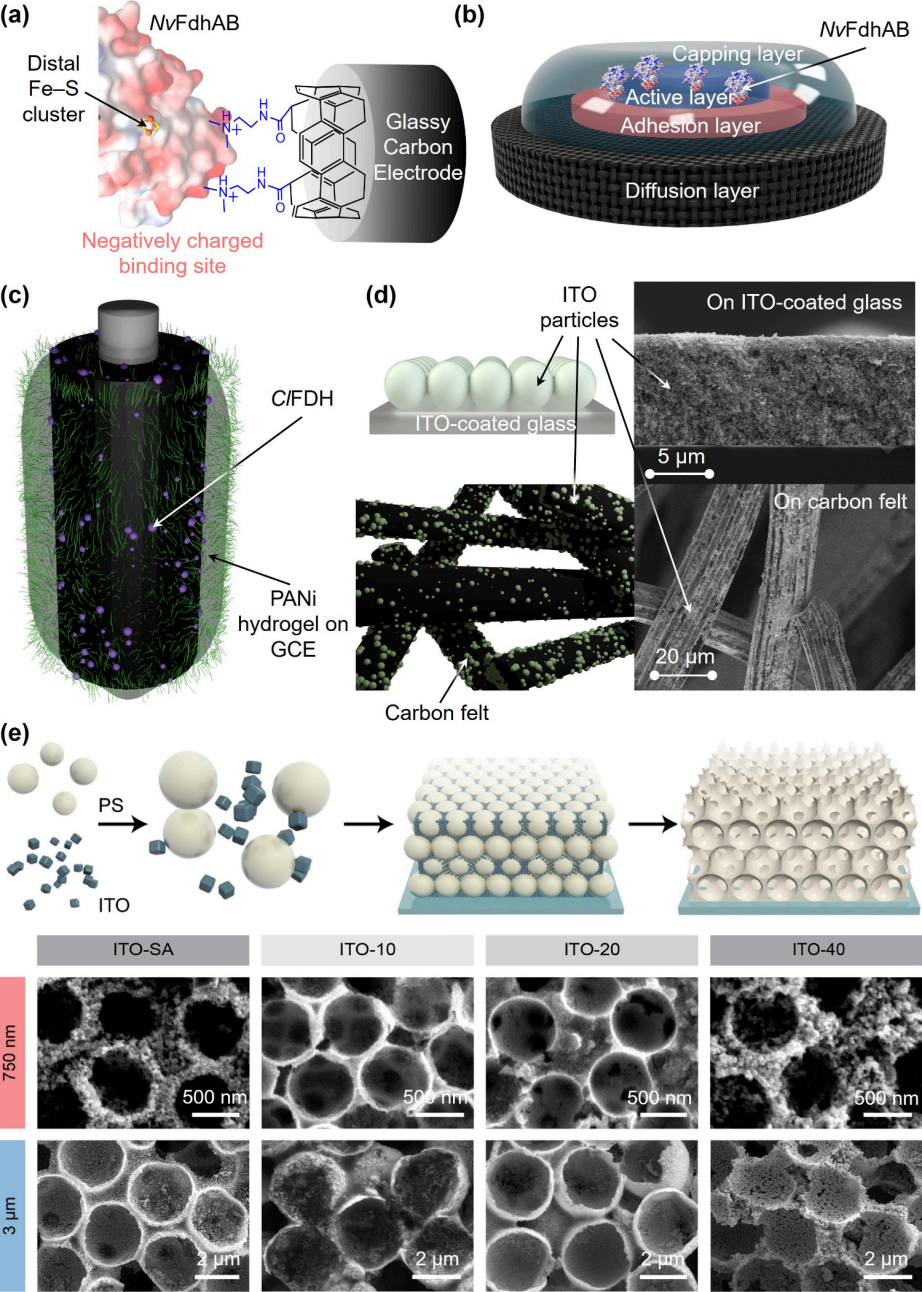
Electrode materials for Fdh integration. (a) CNT-NHMe_2_^+^ modified glassy carbon electrode (GCE) for adsorption of Fdh via electrostatic interactions.^338^ (b) Viologen-modified polymer matrix on carbon cloth-based gas diffusion layer for wiring Fdh.^335^ (c) Polyaniline (PANi) hydrogel conjugated with *Cl*Fdh on a GCE.^305^ (d) Schematics and corresponding SEM images of mesoporous ITO particles sintered on ITO-coated glass^324^ and carbon felt.^353^ The SEM image of ITO|ITO-coated glass is reproduced from ref 324. Copyright 2019 the Authors, published by Wiley-VCH Verlag GmbH & Co. KGaA, under CC BY. The SEM image of ITO|carbon felt is reproduced from ref 353. Copyright 2025 the Authors, published by American Chemical Society, under CC BY. (e) Schematic of the procedures involved in fabricating IO-ITO electrodes using polystyrene (PS) beads to provide a templated structure for the pores along with the SEM images of the electrodes with different pore sizes (750 nm and 3 μm) made with commercial polydispersed ITO nanoparticles (Sigma-Aldrich, ITO-SA) or synthesized monodispersed ITO nanoparticles with average sizes of 10 nm (ITO-10), 20 nm (ITO-20) and 40 nm (ITO-40). The schematic is reproduced from ref 354. Copyright 2020 the Authors, published by National Academy of Sciences, under CC BY. The SEM image of IO-ITO are reproduced from ref 93. Copyright 2019 American Chemical Society, under CC BY.

The reversibility and electrochemical CO_2_ reduction ability of Fdh was first characterized using enzyme-adsorbed graphite electrodes, e.g. PGE electrode, which showcased the fast and selective performance of Fdh.^61,333,334^ Thereafter, studies probed the interfacial association between Fdh and the electrode using electrodes with various functionalization and morphologies, investigating the immobilization of Fdh in the correct electroactive orientation. Beyond that, to achieve high loadings of Fdh and therefore higher current densities, greater formate concentrations and extended stability, 3D-structured porous electrodes such as conjugated polymer matrices and porous metal oxide electrodes were developed. The key performance metrics of notable works on Fdh-immobilized electrodes are listed and compared in Table 4.

**Table 4:** Comparison of Key Performance Metrics across Reported Fdh-Immobilized Electrodes for CO_2_ Reduction in 3-Electrode Configuration^a^

Year	Electrode	Electrode type	Electron transfer type	Electrode area (cm^2^)	Organism	Enzyme(loading)	–J (mA cm^2^)	*E*_app_ (V_SHE_)	FE (%)	Stability (h) (post J%)	Electrolyte	ref
2008	PGE	Stationary	DET	3.94	*S. fumaroxidans*	Fdh1 (5 μg)	0.008	–0.81	97.3	1 (N/A)	MES (0.1 M), NaCl (1 M), NaHCO_3_ (20 mM), pH 6.5	^61^
2014	Graphite-epoxy	Stationary	DET	5.3	*E. coli*	Fdh-H (1 mg mL^–1^)	0.4	–0.60	101.7	1 (2.5%)	MES (0.1 M), Na_2_CO_3_ (20 mM), pH 6.9	^49^
2019	PANi	Hydrogel	DET	1	*C. ljungdahlii*	Fdh (NA)	0.5	–0.40	92.7	12 (40%)	NaHCO_3_ (50 mM), Na_3_PO_4_ (0.1 M), pH 6.5	^305^
2019	ITO|mesoTiO_2_	Stationary	DET	0.25	*Nv*H	FdhAB (43 pmol)	0.1	–0.60	92	2 (88%)	NaHCO_3_ (0.1 M), KCl (50 mM), pH 6.5	^324^
2020	Carbon cloth/redox polymer	Gas diffusion	MET	0.636	*Nv*H	FdhAB (95.4 μg)	0.533	–0.59	3.7	45 (80%)	Phosphate buffer (50 mM), pH 6	^335^
2021	Functionalized LDG	Stationary	DET	0.07	*Nv*H	FdhAB (162 pmol)	0.16	–0.60	>99	1.5 (34%)	Sodium citrate (0.1 M), NaHCO_3_ (50 mM), pH 6	^336^
2021	FTO|IO-TiO_2_	Stationary	DET	0.19	*Nv*H	FdhAB (104 pmol)	4.75	–0.80	96	10 (80%)	MOPS (0.1 M), NaHCO_3_ (1 mM), CsCl (50 mM), pH 4.6	^337^
2022	Functionalized CNT	Stationary	DET	0.071	*Nv*H	FdhAB (40 pmol)	0.25	–0.60	>90	2 (40%)	NaHCO_3_ (0.1 M), KCl (50 mM), pH 6.7	^338^
2022	ITO|mesoITO	Stationary	DET	0.19	*Nv*H	FdhAB (50 pmol)	0.24	N/A^b^	95	2 (100%)^b^	KHCO_3_ (0.1 M), KCl (50 mM), pH 6.67	^339^
2022	FTO|IO-TiO_2_	Stationary	DET	0.19	*Nv*H	FdhAB (104 pmol)	3.6	–0.80	96	10 (72%)	MOPS (0.1 M), NaHCO_3_ (1 mM), KCl (50 mM), pH 4.6	^340^
2023	ITO|mesoITO	Stationary	DET	0.19	*Nv*H	FdhAB^c^ (40 pmol)	0.244	–0.60	96	N/A	MOPS (0.1 M), KCl (50 mM), pH 7^d^	^341^
2023	PGE	Rotating disk (3000 rpm)	DET	0.06	*C. ljungdahlii*	Fdh (180 nmol)	0.125	–0.60	99.3	2 (>100%)	HEPES (0.1 M), NaCl (0.1 M), NaHCO_3_ (0.1 M), pH 7	^333^
2025	Ti foil|IO-TiO_2_	Stationary	DET	0.19	*Nv*H	FdhAB (100 pmol)	1.2	–0.78	95	10 (90%)	NaHCO_3_ (50 mM), KCl (50 mM), pH 6.45	^342^
2024	CNT|CP	Stationary	DET	1	*Tk*	FdhF_HycB3Δ159–184 (95 μg)	0.27	–0.50	99.1^e^	14 (14.8%)	HEPES/NaOH (0.1 M), NaCl (0.1 M), pH 7	^326^
2025	PGE	Stationary	DET	0.1237	*Dd*	Fdh (30 pmol)	1.68	–0.46	N/A	N/A	Acetate/phosphate buffer (0.1 M), NaCl (0.1 M), pH 7	^334^
2025	CF|TiO_2_	Stationary	DET	0.25	*Nv*H	FdhAB (100 pmol)	0.11	–0.59	>99	1 (>99%)	NaHCO_3_ (0.1 M), KCl (50 mM), pH 6.7	^343^
2026	Functionalized CNT	Stationary	DET	1	*S. oneidensis*	*So*FdhAB (150 μg)	–2.9	–0.6	93.1	64 (50%)	HEPES/NaOH (0.1 M) NaCl (0.1 M), pH 7^f^	^194^

a FE: Faradaic efficiency, ITO: indium tin oxide, FTO: fluorine-doped tin oxide, CP: carbon paper.

b Galvanostatic measurements.

c Co-immobilized with carbonic anhydrase.

d 10% CO_2_ instead of saturated CO_2_.

e Measured at 4 h.

f Electrolyte was occasionally refreshed.

Although adsorption of Fdh through drop-casting is often sufficient to enable enzymatic CO_2_ electroreduction,^49^ the orientation of Fdh on the electrode surface is crucial for effective DET. The direct electrocatalysis of CO_2_ by *Dd*Fdh on PGE was found to produce a significantly lower current compared to when a mediator (e.g., methyl viologen) was used to shuttle electrons between *Dd*Fdh and the PGE, i.e. mediated electron transfer (MET).^334^ This was likely due to the limited number of electroactive *Dd*FDH properly oriented on the electrode surface for effective DET, which highlights how desirable enzyme orientation is in the DET regime.

The low potential mediator, methyl viologen (E^0^′ = −0.45 V vs SHE), also enabled the reversible interconversion of HCOO^–^ and CO_2_ by Mo-containing Fdh (FdsDABG) from *C. necator*, consistent with the low Mo^V/IV^ redox potential (E^0^′ = −0.47 V vs NHE, pH 7.5).^224^ Meanwhile, other artificial mediators with higher potentials such as methylene blue (E^0^′ = +0.015 V vs NHE, pH 7) and phenazinum (E^0^′ = +0.085 V vs NHE, pH 7) were more effective as electron acceptors for FdsDABG in formate oxidation.^60,224^

Although effective DET demands specific oriented enzyme immobilization, DET circumvents the disadvantages of MET such as the mass-transport limited kinetics (slow diffusing mediators), the possibility of short-circuit (back) reactions, energy loss, the debilitating costs of mediators and their potential toxicity to microorganisms.^324,344,345^ Therefore, this spurred the development and engineering of electrodes that can immobilize large numbers of Fdh in an electroactive configuration, thereby achieving effective CO_2_ reduction via DET.

In some enzymes, such as the *Nv*FdhAB, the protein surface around the distal Fe–S cluster which is responsible for exchanging electrons with the electrode (Figure 1c and Figure 21e) is negatively charged. Thus, tuning the surface functionalization and electrostatics of the electrode surface is an effective tool to control enzyme immobilization and orientation for DET. Carbon nanotube (CNT) electrodes functionalized with positive (−NHMe_2_^+^) and negative (−COO^–^) functional groups were employed for *Nv*FdhAB immobilization (Figure 22a),^338^ where reversible electrochemical CO_2_ reduction and high catalytic currents were observed for the CNT–NHMe_2_^+^ electrodes, whereas negligible catalytic current was measured for CNT–COO^–^ electrodes. This contrast showcases that the oriented binding of *Nv*FdhAB via the negatively charged distal Fe–S cluster is essential for effective DET and hence the importance of surface charges for enzymatic CO_2_ reduction.

Likewise, low-density graphite (LDG) electrodes functionalized with aminophenyl groups electrostatically oriented the negatively charged acceptor binding site (i.e., the distal Fe–S cluster) of *Nv*FdhAB to the electrode surface, allowing *Nv*FdhAB to be subsequently covalently anchored in an electroactive orientation desirable for DET.^336^ As such, the covalently bound *Nv*FdhAB electrode resulted in high electrocatalytic current by DET for both formate oxidation (−700 μΑ cm^–2^) and CO_2_ reduction (−200 μΑ cm^–2^), obtaining ∼100% FE toward formate during chronoamperometric measurements at −0.6 V vs SHE (pH 6).

Using *Tk*HDCR as a template for AI-assisted mining, an oxygentolerant enzyme, *So*FdhAB, was employed for DET electrocatalytic performance.^194^ The constructed *So*FdhAB/carbon nanotube/glassy carbon electrode (*So*FdhAB/CNT/GC) demonstrated the reversible interconversion of CO_2_ and formate with an onset for CO_2_ at −420 mV vs SHE at pH 7. Notably, the interfacial electron transfer efficiency achieved was >0.9 for CO_2_ reduction at −550 mV vs SHE (pH 6.2) and higher current densities were achieved in the amino-modified and pyrene methylamine-doped CNT electrodes. This was attributed to the hydrogen bond and π-π interactions, respectively, between *So*FdhAB and the electrodes, which work synergistically to drive the oriented immobilization of *So*FdhAB. Most remarkably, excellent oxygen tolerance was demonstrated when a current density of 1.6 mA cm^–2^ was achieved during CO_2_ reduction in gas mixtures containing 20% O_2_, albeit the FE decreased.

While functionalized-2D electrodes (e.g., PGE, LDG) have achieved effective immobilization for CO_2_ reduction by DET, the amount of enzyme that can be effectively immobilized with the correct orientation is restricted to a monolayer and consequently, the current densities and overall performance of the bioelectrocatalytic system is limited.^335,336^

To address this limitation, bespoke 3D electrodes such as polymer matrices and porous metal oxides have been developed for Fdh attachment.^305,324,335^ One example is the viologen-modified polymer matrix (Figure 22b).^335^ This heterogenized MET approach involves the wiring of *Nv*FdhAB to carbon cloth-based gas diffusion layers via an organic low-potential viologen polymer matrix. However, the stability of such mediator-modified polymer electrode is limited due to the narrow range of operating potentials suitable for the mediator and energy (potential)-losses associated with MET. The FE measured toward formate was also observed to be lower than unity. On the contrary, conjugating W-dependent Fdh from *Clostridium ljungdahlii* (*Cl*Fdh) to a conductive polyaniline (PANi) hydrogel (Figure 22c) achieved DET CO_2_ reduction to formate with a FE of 93%.^305^ *Cl*Fdh was previously identified among 20 screened Fdhs to have the highest CO_2_ reduction activity with a *k*_cat_/*K*_M_ value of 183 mM^–1^ s^–1^, and an optimum pH and temperature of 8.6 and 45 °C, respectively.^346^ The quantum mechanics/molecular mechanics-based computation study conducted for the *Cl*Fdh-conjugated PANi hydrogel suggested a possible electron transfer pathway from the benzenoid amines in the PANi hydrogel to the Arg36 surface residue, the [4Fe–4S] cluster and finally to the W metal center.^305^

Another type of 3D electrode is the porous metal oxide electrode, which allows for high enzyme loading, stable enzyme binding, and efficient DET to the active site of Fdh (Figure 22d).^337,347^ The strong and active enzyme attachment is attributed to the high affinity between the glutamic and aspartic acid residues on the protein surface to metal oxides, which has been originally reported for [NiFeSe]-hydrogenase from *Desulfomicrobium baculatum* and TiO_2_.^69,348–350^ This established binding hence forms the basis for the further development of metal oxide electrodes for immobilizing enzymes, such as Fdh for CO_2_ reduction. The two types of metal oxides commonly used for Fdh immobilization are indium tin oxide (ITO) and TiO_2_. ITO is a conductive material (degenerate semiconductor) but is unstable at more reducing potentials (<−0.6 V vs SHE, pH 6.7) and is hence more suited for probing the reversible nature of Fdh at mild potentials.^351^ Meanwhile, TiO_2_ is a semiconductor insulating at mild potentials but stable and conducting at more reducing potentials (<−0.6 V vs SHE, pH 6.7), which makes it suitable for application in CO_2_ reduction devices.^352^

The use of metal oxides was investigated for PFV by immobilizing *Nv*FdhAB on mesoporous ITO (Fdh|mesoITO) and TiO_2_ (Fdh|mesoTiO_2_) electrodes with a film thickness of ∼2.5 μm.^324^ The particle sizes of the mesoporous ITO and TiO_2_ were <50 nm and ∼21 nm, respectively. After demonstrating the reversible interconversion of CO_2_ and formate using the Fdh|mesoITO electrode, an Fdh|mesoTiO_2_ electrode was employed for CO_2_ reduction, achieving a current density of −100 μA cm^–2^ at −0.6 V vs SHE (pH 6.5). The interaction of *Nv*FdhAB with TiO_2_ was also probed using a quartz crystal microbalance (QCM) cell (see Section 5.3), which suggested contributions from chemisorption beyond pure electrostatic interaction. Hence, strong attachment between the enzyme and TiO_2_ is established for efficient electron transfer and thereby, CO_2_ reduction catalysis. The saturated enzyme loading on TiO_2_ was shown to be approximately 3.5 pmol cm^–2^.

Progressing from flat and mesoporous electrodes, metal oxide electrodes with a hierarchical macroporous inverse opal (IO) architecture (Figure 22e) were also developed,^89^ significantly improving enzyme adsorption and the penetration of substrates and products. These IO structures are highly ordered, 3D nanoporous materials suitable for high loading of enzymes. The endowment of a mesoporous frame further increases the effective surface area for enzyme interactions and enables stable enzyme immobilization. Furthermore, the tunability of the dimensions of macro/meso pores and film thickness render these hierarchical electrodes versatile for enzyme integration. Pore sizes of 750 nm are typically used for enzyme immobilization, but for larger microbiological components such as bacteria, 10 μm pore sizes are typically used. Following the first report of ITO electrode with IO architecture and mesoporous skeleton,^89^ similar electrodes were employed for the immobilization of Fdh as (photo)­electrodes for CO_2_ reduction.^151,337^ IO-TiO_2_ electrodes adsorbed with *Nv*FdhAB (IO-TiO_2_|Fdh) attained a remarkably high current density of (−4.75 mA cm^–2^ at −0.53 V vs RHE) with the use of CO_2_-saturated CsCl electrolyte (pH 4.6).^337^ After 10 h of controlled potential electrolysis, a FE of 96% toward formate, a turnover number (TON) of 1.5 × 10^6^, and TOF of 42 s^–1^ were achieved. The IO-TiO_2_|Fdh electrode was also combined with a perovskite to assemble a photoelectrode (see Section 7).

### 5.2 Understanding the Local Environments in Fdh Electrocatalysis

The use of porous 3D electrodes results in the nanoconfinement of enzymes, which over the course of catalysis, establishes local chemical environments at the electrode surface (pH, substrate and product concentration) distinct from the bulk solution.^339,355,356^ As enzyme activity is highly sensitive to the precise conditions of these local chemical environments, in depth understanding and careful tuning of the local environment is essential to achieve optimal performance of the enzyme-immobilized porous electrodes.^340^ While direct measurement of these local environments is either challenging or currently not possible, the use of finite element modeling (FEM) is a powerful technique for analyzing and simulating these systems to gain insights inaccessible by experimental methods.^339^ Briefly, FEM models are built using fundamental physical equations and known enzyme-activity dependencies, and is then validated with experimental results before applying the model to simulate new case scenarios.

For example, a 2D axisymmetric tertiary current distribution model was developed to represent an Fdh-immobilized mesoITO film on FTO-coated glass electrode (Figure 23a).^340^ Using analytical expressions such as the Nernst–Planck equation, Michaelis–Menten kinetics and mass transport model, the dependence of enzyme catalytic current on applied potential, pH and substrate concentrations were calculated over a range of conditions. Upon validating the model, i.e. predicted currents matched well with experimental values, the model was employed to simulate changes in local pH and other species concentrations.

**Figure 23:**
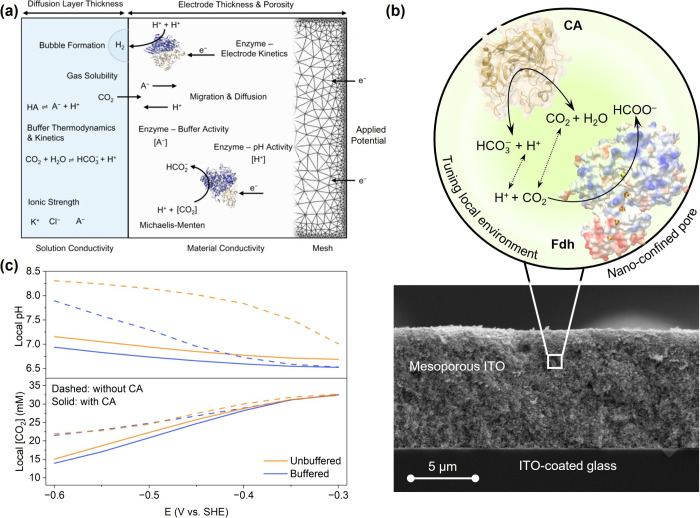
Tuning of local electrode environment for enhanced catalytic performance. (a) Illustration of the different parameters considered in developing the finite element model (FEM), such as enzyme activity dependence, enzyme–electrode kinetics, geometry parameters, solution properties, and potential distributions. Reproduced from ref 340. Copyright 2022 the Authors, published by PNAS, under CC BY. (b) Schematic of coimmobilized carbonic anhydrase (CA, PDB: 1V9E)^364^ with *Nv*FdhAB (PDB: 6SDV),^51^ depicting the nanoconfinement of both enzymes. The SEM image of a mesoporous ITO electrode is reproduced from ref 339. Copyright 2022 the Authors and Springer Nature Limited. (c) FEM simulation results for the average pH and CO_2_ concentration within the mesoporous electrode at steady state. Solid lines and dashed lines represent results with and without CA respectively. Solution conditions: orange, CO_2_-purged 0.1 M KHCO_3_+0.05 M KCl (pH 6.67); blue, CO_2_-purged 0.05 M KHCO_3_+0.05 M MES+0.05 M KCl (pH 6.45). Numerical data adapted from ref 339. Copyright 2022 the Authors and Springer Nature Limited.

Local pH is an important parameter of the local environment that affects the activity of *Nv*FdhAB as seen in previously reported pH-activity solution assays, revealing the optimal pH of *Nv*FdhAB to be 7.1.^51^ Given the net consumption of protons during CO_2_ electroreduction to formate by Fdh, the local pH at the electrode surface is expected to increase during the electroreduction of CO_2_. Employing the model built, the local pH in *Nv*FdhAB-immobilized mesoITO electrodes was found to increase by around 2 pH units, resulting in a drastic decrease in activity (∼5 times lower).^340^ The local pH was better maintained when the buffer capacity was increased with the addition of zwitterionic Good’s buffers or by adding carbonic anhydrase (CA) to accelerate the conversion of CO_2_ to H^+^ and HCO_3_^–^. Furthermore, instead of selecting the bulk pH for optimal enzyme activity, a lower bulk pH should be selected to cater for subsequent local pH changes, which then leads to the optimum pH for enzyme activity within the local environment. Apart from tuning the buffer components in the electrolyte, a less dense porous electrode structure (e.g., hierarchical IO electrodes) was also shown to benefit mass transport and alleviate unfavorable local environments.

Good’s buffers are prevalently used for enzymatic electrocatalysis due to the several advantages they possess, namely, (i) consisting of fast-buffering species, (ii) having good buffer capacity at the local pH optimum, (iii) not prone to coordinate with the metals in the enzymes, and (iv) physically and chemically inert (i.e., noninteracting) toward the reference electrode.^357–360^ However, the presence of Good’s buffers has shown to inhibit enzyme activity,^340,361^ actively contribute to catalytic mechanism as a proton donor,^362,363^ and act as noninnocent electrolytes which can be oxidized at mild potentials in the presence of an anode or photoelectrochemical setups (e.g., in a one-compartment cell).^337^ The extra components introduced with the use of additional buffers also complicate product separation or downstream use of products after catalysis.^339^

Instead of utilizing additional buffers (e.g., Good’s buffer), the effects of local pH changes can also be mitigated by increasing the kinetics of CO_2_ hydration such that CO_2_/HCO_3_^‑^ can act as an active buffer for the local environment. This has been explored by the coimmobilization of CA and *Nv*FdhAB on planar ITO (Figure 23b).^339^ By building a FEM model that comprises electrode and diffusion layer geometry, enzyme and solution kinetics as well as enzyme activity factors and mass transport, the role of CA in changing the local pH and local concentration of CO_2_ and proton donors was understood. Upon CA coimmobilization, enhanced hydration of CO_2_ (i.e., equilibrium shifts toward to HCO_3_^‑^ and H^+^) in response to proton consumption during catalysis maintains the optimal local pH (Figure 23c), resulting in increased *Nv*FdhAB activity and formate production. Notably, the lowered CO_2_ concentration arising from the shift in the equilibrium has little effect on *Nv*FdhAB activity due to the high affinity of *Nv*FdhAB for CO_2_ (Michaelis–Menten constant, *K*_M_ = 0.420 mM). For comparison, the coimmobilization of CA worsens the performance of heterogeneous Au catalyst wherein the increased concentration of H^+^ leads to greater hydrogen evolution which is compounded by the reduced concentration of CO_2_ that adversely affects the rate of CO_2_ reduction.

The advantages of coimmobilizing CA for enzymatic catalysis have been confirmed by recent works, highlighting how CA tunes the local chemical equilibrium, enhances (photo)­electrochemical performance, and replaces the use of Good’s buffers to enable the deployment of integrated devices in one-compartment systems.^342,365^

Apart from mitigating local pH changes, CA was also employed to accelerate the interconversion between CO_2_ and HCO_3_^−^ to conclusively prove that CO_2_ is indiscriminately the substrate for the reductive reaction of Fdh, rather than HCO_3_^–^.^250^ This was carried out in a PFE system with Fdh immobilized on a rotating disc electrode and the evolution of catalytic current with time was observed upon injections of CO_2_ or HCO_3_^–^ in the presence or absence of CA in solution. In the absence of CA, the current increases slowly upon injection of carbonate, corresponding to the slow transformation of bicarbonate into CO_2_, while when CO_2_ was injected, a rapid current increase is observed suggesting the CO_2_ is the actual substrate of the enzyme. In the presence of CA, whether CO_2_ or HCO_3_^–^ was injected, CA quickly shifts the equilibrium away or toward CO_2_ respectively, resulting in comparable current traces.

CA has also been employed to increase local CO_2_ concentration for utilization.^366^ By biomimicking a carboxysome, CA was utilized to accelerate the conversion of HCO_3_^–^ to CO_2_ with the aim of raising local CO_2_ levels in a nanoconfined electrode to enable CO_2_ electrolysis in dilute CO_2_ streams.^341^ However, the fast hydration kinetics of CA coimmobilization does not improve formate production under conditions of low CO_2_ concentration. While the current densities with and without CA were comparable, the employed FEM model revealed that CO_2_ now acts as both the substrate and buffer, and local CO_2_ concentration becomes critical toward Fdh activity. As a result, the decrease in local pH to maintain optimal local pH is counteracted by the additional lowering of local CO_2_ concentration, overall inhibiting enzymatic activity under dilute CO_2_ conditions. To address this issue, CA was used together with kinetically fast Good’s buffer (e.g., MOPS) to counterbalance the local increase in pH. In this case, CA coimmobilization promotes the conversion of HCO_3_^−^ and H^+^ to CO_2_, which leads to the replenishment of CO_2_ that depleted during electrolysis, resulting in a significant improvement in CO_2_ reduction activity under the conditions of dilute CO_2_ concentrations down to atmospheric concentrations.^341^ By mitigating local pH increases and enabling the utilization of dilute CO_2_ concentrations, these studies pave the development of Fdh systems toward longer term operational stability and applicability.

### 5.3 Quartz Crystal Microbalance and ECQCM

Quartz crystal microbalance (QCM) is a highly sensitive technique used to measure changes in mass per unit area by detecting variations in the frequency of a quartz crystal oscillator. It operates using the piezoelectric effect, where an alternating voltage causes the quartz crystal to oscillate at its resonant frequency.^367–369^ When a biomolecule, such as Fdh, adsorbs onto the surface of the crystal, the added mass causes a decrease in the resonant frequency. This frequency shift (Δ*f*) is directly proportional to the mass change on the surface of the quartz crystal oscillator, defined by the Sauerbrey equation (eq 4).^370–372^

4 $$\Delta f=-\frac{2f_{0}^{2}}{A\sqrt{p_{q}\mu_{q}}}\Delta m$$

where *f*_0_ is the resonant frequency of the quartz crystal oscillator, *A* is the piezoelectrically active crystal area, Δ*m* is the change in mass, *p*_*q*_ is the density of quartz, and μ_*q*_ is the shear modulus of quartz. QCM is capable of detecting mass changes in the range of nanogram to microgram per square centimeter. This sensitivity makes it an invaluable tool for studying surface interactions between Fdh and electrodes, enabling precise analysis of adsorption processes (Figure 24).

**Figure 24:**
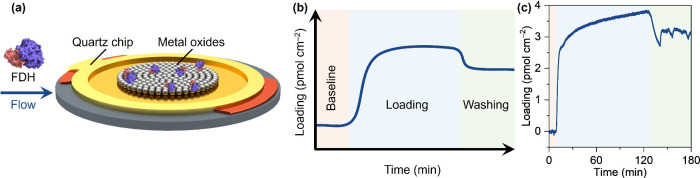
(a) Schematic illustration of a metal oxide-functionalized quartz chip. (b) An idealized schematic QCM profile for monitoring enzyme adsorption and desorption. (c) A representative experimental QCM profile of Fdh immobilization on a SrTiO_3_-functionalized quartz chip. Numerical data adapted from ref 373. Copyright 2024 the Authors, published by American Chemical Society, under CC BY.

In addition to detecting mass changes, QCM with dissipation monitoring (QCM-D) provides insights into the viscoelastic properties of the adsorbed layer by analyzing the dissipation factor, which reflects energy loss during oscillation.^374–376^ This feature is particularly valuable for studying soft or viscous layers such as polymers or biological molecules.^73,377,378^ By integrating data on mass and dissipation, QCM-D enables detailed assessment of Fdh adsorption, desorption, assembly, and orientation. Moreover, the quartz crystal oscillator can be functionalized with a thin layer of electrode material,^379,380^ such as self-assembled monolayers or metal oxides, allowing it to function as a working electrode in an electrochemical cell. This technique, known as electrochemical QCM (EQCM),^381–383^ is a powerful tool for real-time monitoring of mass changes associated with Fdh–electrode interactions during electrochemical reactions.

The first application of QCM in Fdh research was for the construction of a mediated enzyme film formate biosensor, where EQCM was employed to monitor the deposition of constituent layers.^384^ This biosensor composed of a layer of polypyrrole film embedded with Fdh from *Candida boidinii* and NAD^+^, as well as a Prussian blue (PB) film which acts as the mediator. Through monitoring the simultaneous current and frequency changes via EQCM, two distinct electrodeposition mechanisms with PB deposited above or below the polypyrrole film were studied. The layered structure with PB under the polypyrrole film was found to be superior, achieving very stable current and frequency responses during deposition. The bilayer structure served as a formate biosensor where the Fdh in the polypyrrole film oxidizes formate to CO_2_ and reduces NAD^+^ to NADH, and subsequently the underlying PB film regenerates NAD^+^ from NADH, mediating the electron transfer to the electrode which is recorded as a current signal.

In contrast to MET systems, direct interfacing of Fdh with an electrode offers a straightforward DET configuration, achievable through rational interfacial modifications such as covalent bonding and electrostatic interactions. The covalent immobilization of *Nv*FdhAB on chemically modified gold and graphite electrodes for bioelectrocatalytic CO_2_-to-formate conversion has been investigated using QCM-D.^336^ This process involves forming a self-assembled monolayer of 4-aminothiophenol on a gold-coated quartz crystal surface, where the positively charged amino groups link with the negatively charged carboxyl groups of *Nv*FdhAB, achieving an enzyme coverage of 8.6 ± 0.2 pmol cm^–2^. The resulting compact Fdh layer on the electrode surface exhibits a current density of −200 μA cm^–2^ at −0.66 V vs SHE (pH 6), with nearly 100% FE in a three-electrode configuration.

Fdh–electrode interactions have been further investigated using EQCM on five different thiol-based self-assembled monolayers on gold electrodes, confirming that the synergy between electrostatic interactions and hydrogen bonding enhances the electrocatalytic activity of *Nv*FdhAB.^385^ Interfacing *Nv*FdhAB with 2-dimethylammoniumethanethiol, which possesses both a positive charge (p*K*_a_ = 7.6) and hydrogen bonding capabilities, results in the highest current density of 21 μA cm^–2^ at +0.5 V vs RHE for formate oxidation. When the enzyme is correctly oriented electrostatically, the addition of viologen-based redox mediators shows no increase in current density, indicating nearly quantitative binding of the enzymes in the correct orientation for DET, as evidenced by a near-unity DET/MET current density ratio.

A more complex system that combines porosity and electrostatics is demonstrated by functionalizing a quartz crystal chip with CNTs featuring either positive (tertiary amine) or negative (carboxylic) surface groups.^338^ Despite similar *Nv*FdhAB loadings on both positive CNTs (7.7 ± 0.47 pmol cm^–2^) and negative CNTs (6.6 ± 0.63 pmol cm^–2^) after 2 h, the immobilized Fdh on carboxylic CNTs shows no DET currents. In contrast, tertiary amine CNTs support *Nv*FdhAB for the reversible electrocatalytic interconversion of CO_2_ and formate, achieving >90% FE with a current density of −247 μA cm^–2^ at −0.6 V vs SHE for CO_2_ reduction and +246 μA cm^–2^ at +0.1 V vs SHE for formate oxidation.

In addition to carbonaceous materials, metal oxides have shown significant promise for binding Fdh in its electroactive orientations. The interaction of *Nv*FdhAB with TiO_2_ for electrocatalysis and photocatalysis has been investigated using QCM analysis, revealing a loading of 3.5 pmol cm^–2^ on a planar TiO_2_-coated quartz chip after 2 h.^324^ The strong binding between *Nv*FdhAB and TiO_2_ is demonstrated by a series of washing experiments: after rinsing the QCM cell with an enzyme-free solution for 1 h, 94% of the preloaded *Nv*FdhAB remains adsorbed. Even with KCl concentrations increased to 0.5–3.0 M, only 30–40% of *Nv*FdhAB desorbed from the TiO_2_ surface, indicating strong chemisorption, likely involving amino acids such as aspartic and glutamic acid.

QCM studies can also guide the loading of *Nv*FdhAB on porous ITO electrodes, revealing a saturated surface coverage of 4.2 pmol cm^–2^ on a planar ITO-coated quartz chip.^339^ A mesoporous ITO electrode, with a geometric surface area of 0.19 cm^2^ and an absolute surface area of 139 cm^2^, can accommodate a theoretical maximum of 530 pmol *Nv*FdhAB. Co-immobilization of *Nv*FdhAB and carbonic anhydrase on an ITO-coated QCM chip demonstrates stable enzyme coloading of up to 4.5 pmol cm^–2^ over 2 h. Likewise, QCM analysis of *Nv*FdhAB interactions with SrTiO_3_:La,Rh shows a two-stage adsorption process: a rapid initial adsorption over 13 min followed by a slower approach to saturation at 3.8 pmol cm^–2^.^373^ This process is a standard kinetic behavior governed by surface availability, molecular diffusion, and structural reorganization. The strong binding at the SrTiO_3_:La,Rh|Fdh interface is evidenced by a subsequent 1 h washing section, during which only 18% of *Nv*FdhAB is is desorbed (Figure 24c).

### 5.4 Infrared Spectroscopy

Infrared (IR) spectroscopy is a widely utilized analytical technique in enzyme studies, offering detailed insights into enzyme structure, function, and dynamics.^386–389^ By measuring the absorption of IR light by molecular bonds, IR spectroscopy provides a molecular fingerprint that reflects the specific vibrational modes of various functional groups present in Fdh.^271,390,391^ Fourier-transform IR (FTIR) spectroscopy allows for the collection of high-resolution spectra over a wide range of wavenumbers in real-time.^392–395^ This is beneficial for monitoring dynamic processes in Fdh at a molecular level, such as conformational changes that occur upon enzyme immobilization.^283,396,397^ FTIR can track these changes by observing shifts in specific IR bands, offering insights into the structural dynamics of Fdh under various conditions. It is particularly useful for studying metal cofactors, Fdh–substrate binding, Fdh–electrode interactions, and changes in secondary structure.^117,398–400^

By monitoring the vibrational modes of specific bonds within the active site, IR spectroscopy provides valuable insights into substrate and inhibitor binding, complex formation, and the mechanisms of catalysis and inhibition. Notably, azide, an inhibitor of Fdh, also serves as an effective IR probe, enabling vibrational spectroscopic investigations of Fdh dynamics on the femtosecond time scale.^117,401–403^ Changes in the IR spectra can reveal the formation or breaking of chemical bonds during reactions, offering direct evidence of intermediate states and the overall catalytic or inhibitory pathways. For instance, a recent IR spectroscopy study on Mo-dependent Fdh from *Rc*Fdh identified distinct competitive and noncompetitive binding sites within the enzyme’s secondary coordination sphere, using azide and cyanate as model inhibitors.^283^ Site-directed mutagenesis revealed the involvement of key amino acids near the bis-MGD cofactor in these binding interactions, particularly Arg587 and His387. This study suggests that these inhibitors can serve as models for understanding substrate binding in Fdh, potentially stabilizing formate/CO_2_ during catalysis and protecting the enzyme from oxidative damage.

IR spectroscopy is also useful for examining the secondary structure of enzymes (Figure 25). Techniques such as attenuated total reflectance FTIR (ATR-FTIR) can provide information about the structural motifs by analyzing the IR absorption of amide I band and amide II band.^387,404^ The amide I band, which occurs in the range of 1700–1600 cm^–1^, arises primarily from the C=O stretching vibrations of the peptide bonds. This band is highly sensitive to the secondary structure of the protein, such as α-helices and β-sheets. The amide II band, found in the range of 1600–1500 cm^–1^, results primarily from N–H bending and C–N stretching vibrations in the peptide bonds. In Fdh research, the analysis of amide I and II bands using ATR-FTIR spectroscopy is essential for understanding the structural integrity and dynamics of Fdh.^405^ Changes in these bands can indicate conformational changes in the enzyme that are associated with substrate binding, catalysis, or inhibition. By functionalizing the ATR-FTIR prism with electrode materials, this technique can offer valuable insights into the immobilization and infiltration process of Fdh on the electrode surface in real time.

**Figure 25:**
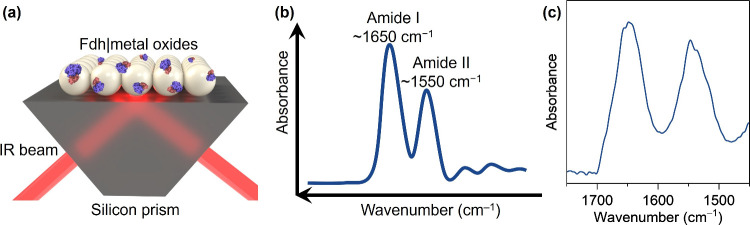
(a) Schematic illustration of a functionalized IR prism. (b) An idealized schematic IR spectrum for studying the secondary and tertiary structures of enzymes. (c) A representative experimental IR spectrum of Fdh immobilization on a CNT-functionalized IR prism. Numerical data adapted from ref 338. Copyright 2022 the Authors, published by American Chemical Society, under CC BY.

For example, the *Nv*FdhAB adsorption on TiO_2_ surface was studied using ATR-FTIR spectroscopy on a 100 nm planar or a 400 nm mesoporous TiO_2_ layer-coated Si prism.^324^ After adding *Nv*FdhAB, characteristic amide I (1650 cm^–1^) and II (1545 cm^–1^) bands were detected, indicating the enzyme backbone’s structure. The adsorption process was monitored in situ for 2 h, showing minimal changes in the amide bands, suggesting a largely retained *Nv*FdhAB structure on TiO_2_ surfaces. Amide band intensities increased over time, and most *Nv*FdhAB remained adsorbed even with increased ionic strength, indicating strong associations between *Nv*FdhAB and TiO_2_ beyond purely electrostatic interactions.

To confirm *Nv*FdhAB’s structural integrity upon adsorption on carbonaceous electrodes, ATR-IR spectroscopy was conducted using Si prisms coated with positively or negatively charged CNT films.^338^ The thin CNT membrane (∼76 nm) allowed immediate detection of *Nv*FdhAB upon adsorption. Amide I and II bands at 1647 and 1541 cm^–1^ confirmed *Nv*FdhAB adsorption on both CNT films with different charges. By plotting the amide bands intensities overtime, the adsorption kinetics showed most loading occurred within the first 20 min, with retained secondary structure evident from consistent amide band shapes. Comparatively, a control with denatured *Nv*FdhAB showed significant amide I band broadening due to loss of the secondary structure.

### 5.5 Electrochemical Impedance Spectroscopy

Electrochemical impedance spectroscopy (EIS) is a transfer-function measurement used to measure the impedance of an electrochemical system over a range of frequencies.^406–409^ By applying a small alternating current (AC) voltage perturbation (typically sinusoidal modulation) to a working electrode, EIS records the resulting current response. Based on the Ohm’s law, the impedance (*Z*(ω)) is calculated as the ratio of the complex voltage (*Ṽ*(ω)) to the current (*Ĩ*(ω)):^410–413^

$$Z\left(\omega \right)=\frac{Ṽ\left(\omega \right)}{Ĩ\left(\omega \right)}=\left|Z\right|\left(\cos\varphi \left(\omega \right)+i\;\sin\varphi \left(\omega \right)\right)=Z_{r}+iZ_{i}$$

where ω is the angular frequency, |*Z*| is the magnitude of impedance, φ is the phase angle shift between the input voltage and the output current, *i* is the imaginary number, and *Z*_*r*_ and *Z*_*i*_ are the real and imaginary part of the impedance, respectively.

EIS data are typically presented in the forms of Nyquist plot or Bode plot (Figure 26).^414–417^ The Nyquist plot displays *Z*_*r*_ on the *x*-axis and *Z*_*i*_ on the *y*-axis. Each point on the plot corresponds to the impedance value at a specific frequency, with the highest frequencies typically near the origin and lower frequencies further along the curve. Note that Nyquist plots should have equally scaled *x*-axis and *y*-axis to allow the assessment of circularity.^409,415^ The Bode plot is classified as the magnitude (|*Z*|) plot or the phase angle (φ) plot, both of which represent impedance as a function of frequency. The *x*-axis, typically displayed on a logarithmic scale, represents the frequency, while the *y*-axis shows |*Z*| in the Bode magnitude plot and φ in the Bode phase plot.

**Figure 26:**
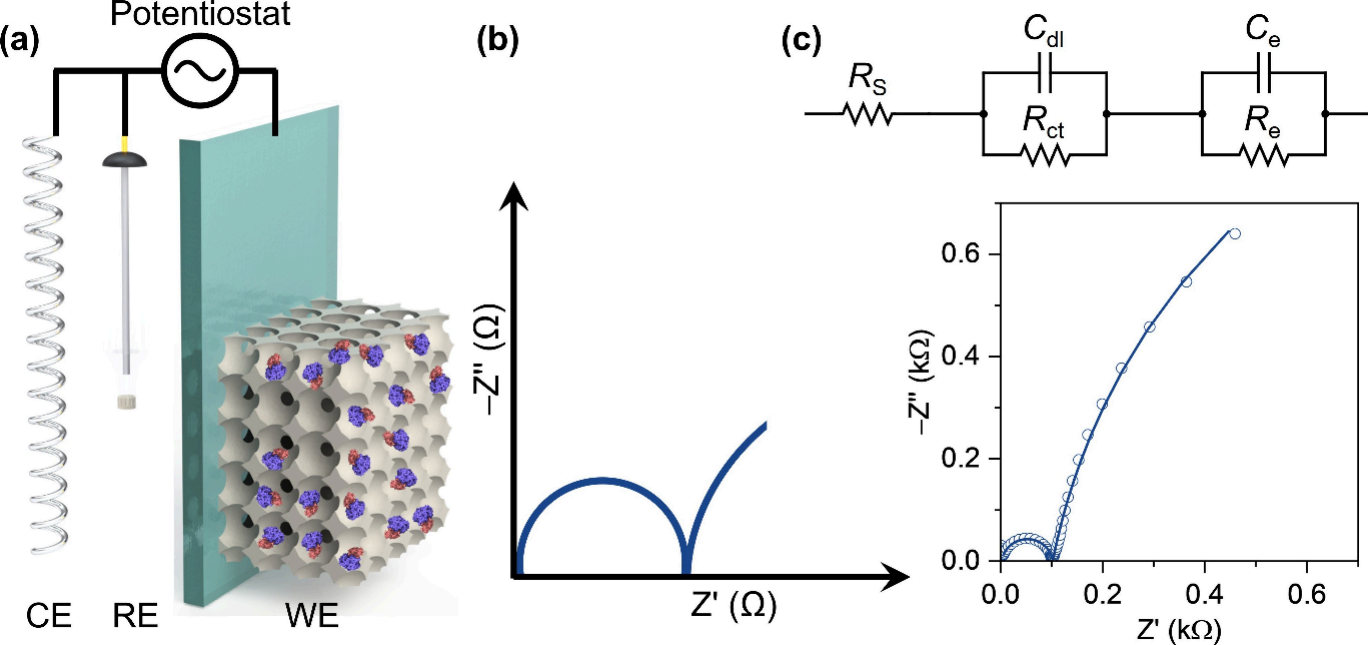
(a) Schematic illustration of a 3-electrode EIS setup consisting of an IO-TiO_2_|Fdh (PDB: 6SDV)^51^ working electrode (WE), a Pt counter electrode (RE), and a Ag/AgCl reference electrode (RE). (b) An idealized schematic impedance response in Nyquist plot. (c) A representative experimental Nyquist plot and corresponding equivalent circuit to quantitatively evaluate charge transfer and recombination at interfaces. Conditions: 500 kHz to 0.1 Hz and a 25 mV sinusoidal amplitude, −0.2 V vs RHE. Numerical data adapted from ref 342. Copyright 2025 the Authors, published by Elsevier Inc., under CC BY.

EIS data can be interpreted using both qualitative and quantitative methods.^409,418^ Qualitative interpretation involves analyzing the shape and features of Nyquist plots to understand the overall behavior of the system. In a Nyquist plot, each semicircle typically corresponds to a distinct electrochemical process, such as charge transfer at the enzyme–electrode interface,^419–421^ electrical double layer behavior,^422–424^ or electrode resistance.^425–427^ The diameter of each semicircle is indicative of the resistance associated with that process, making it a widely used approach for estimating charge transfer resistance (*R*_*ct*_). The high-frequency intercept (HFI) is the point where the impedance response intersects the real axis in the high-frequency region (MHz–kHz) of a Nyquist plot.^428–430^ It typically represents the series resistance (*R*_*S*_), which includes contributions from cables and electrolyte resistance. In contrast, the low-frequency region (Hz–mHz) of the Nyquist plot is dominated by mass transport and slower kinetic processes.^431–433^ A characteristic feature in this region is a straight line with a slope close to 45-degree, which is indicative of a diffusion-controlled process known as Warburg impedance.^434–436^

Qualitative EIS analysis investigated Fdh bioelectrocatalysis on a CNT-modified graphite electrode with a polyethylenimine (PEI) protective layer, achieving a current density of up to 0.23 mA cm^–2^ and stability for 11 h.^421^ Subsequent EIS measurements revealed the role of each modification layer in influencing the *R*_*ct*_, which is estimated from the semicircle diameter in a Nyquist plot. The deposition of CNTs on the graphite electrode reduced *R*_*ct*_ from 24 Ω to 10 Ω, indicating enhanced conductivity. Modification of the electrode with nitrophenyl (NP) functional groups using 2 mM diazonium salt increased *R*_*ct*_ to 26 Ω. Following the reduction of these groups to positively charged aminophenyl functional groups, *R*_*ct*_ decreased to 9 Ω. However, using a higher concentration of diazonium salt (20 mM) significantly increased *R*_*ct*_ to 330 Ω, suggesting the formation of insulating nitroaryl multilayers. The immobilization of Fdh led to a slight increase in *R*_*ct*_ due to the insulating nature of peptide chains, while the final addition of a PEI protective layer reduced *R*_*ct*_.

Quantitative interpretation of complex impedance data relies on equivalent circuit fitting, which models the electrochemical system as a combination of electrical components such as resistors, capacitors, inductors, and Warburg elements.^409,412,437–440,440^ By fitting the experimental impedance data to this model, various electrochemical processes including charge transfer, diffusion, recombination, and charge storage at the electrical double layer can be quantitatively described in terms of resistance and capacitance.^441–443^ To account for nonideal capacitive behavior, often caused by surface roughness or frequency-dependent dielectric constants,^444–446^ a constant phase element (CPE) is frequently employed in equivalent circuit fitting to interchange with a capacitor.^447–449^ Selecting an appropriate equivalent circuit model requires a thorough understanding of the electrochemical system and should accurately represent all the expected electrochemical processes during bioelectrocatalysis. The model must be carefully chosen to avoid overfitting, which, despite potentially yielding a better perceived fitting result, can lead to results that lack physical meaning.^450,451^ The Randles circuit comprising *R*_*S*_, *R*_*ct*_, and double-layer capacitance (*C*_*dl*_), with a Warburg element (*Z*_*W*_) included if diffusion processes are significant, is the most commonly used model for describing bioelectrocatalytic systems.^452^

Quantitative EIS analysis of Fdh electrochemistry was performed to elucidate charge carrier behavior on an Fdh-immobilized IO-TiO_2_ electrode (Figure 26).^342^ The analysis employed three distinct equivalent circuits that consists of electrical components such as a *R*_*S*_ to describe cell resistance; a *C*_*dl*_ in parallel with a series combination of a *R*_*ct*_ and a *Z*_*W*_ to elucidate the electrical double layer; and an RC circuit (*R*_*e*_, *C*_*e*_) to depict electron transfer in the Faradaic process. These circuits were used to fit impedance data for the bare IO-TiO_2_ electrode, the IO-TiO_2_|Fdh electrode before the onset potential, and the IO-TiO_2_|Fdh electrode after the onset potential, respectively. The results showed that *R*_*e*_ on the IO-TiO_2_|Fdh electrodes decreased with increasing cathodic potential due to insulating nature of TiO_2_ and further decreased in the presence of carbonic anhydrase. Additionally, *C*_*dl*_ of IO-TiO_2_|Fdh electrodes increased as the potential shifted toward more cathodic values, suggesting an increased local HCOO^–^ ion concentration due to the bioelectrocatalytic CO_2_ reduction to formate. The presence of CA, which consumes CO_2_ and water to maintain the local proton concentration during formate production, effectively stabilized the *C*_*dl*_ from +0.2 to −0.4 V vs RHE, indicating its role in maintaining the local environment of an electrochemical system for CO_2_ reduction.

## 6 Deployment of Fdh Systems in CO_2_ Electrolysis Devices

While electrodes (Section 5.1), using PFE using a well-controlled three-electrode configuration serves as a useful characterization technique for studying and optimizing Fdh-immobilized electrodes, the established understanding and insights also provide a foundation for the application and further development of Fdh systems in larger scale or device-type CO_2_ electrolysis.

CO_2_ electrolyzers are an essential pivot for advancing the use of electrocatalysts (e.g., heterogeneous catalysts, small molecule catalysts) toward greater technological relevance. Some parallels can be drawn between the architecture of a typical CO_2_ electrolyzer and the structure of Fdh (Figure 27),^55^ for example, the flow and housing plates that holds the gas diffusion electrodes (GDE) and polymer electrolyte membrane are analogous to the outer protein scaffold, [4Fe–4S] clusters and ion transport channels, while the hydrophobic gas diffusion layers of the GDE is akin to the hydrophobic channels in enzymes, and finally, the catalyst and other additives deposited on the are similar to the enzymatic active site and secondary coordination sphere.

**Figure 27:**
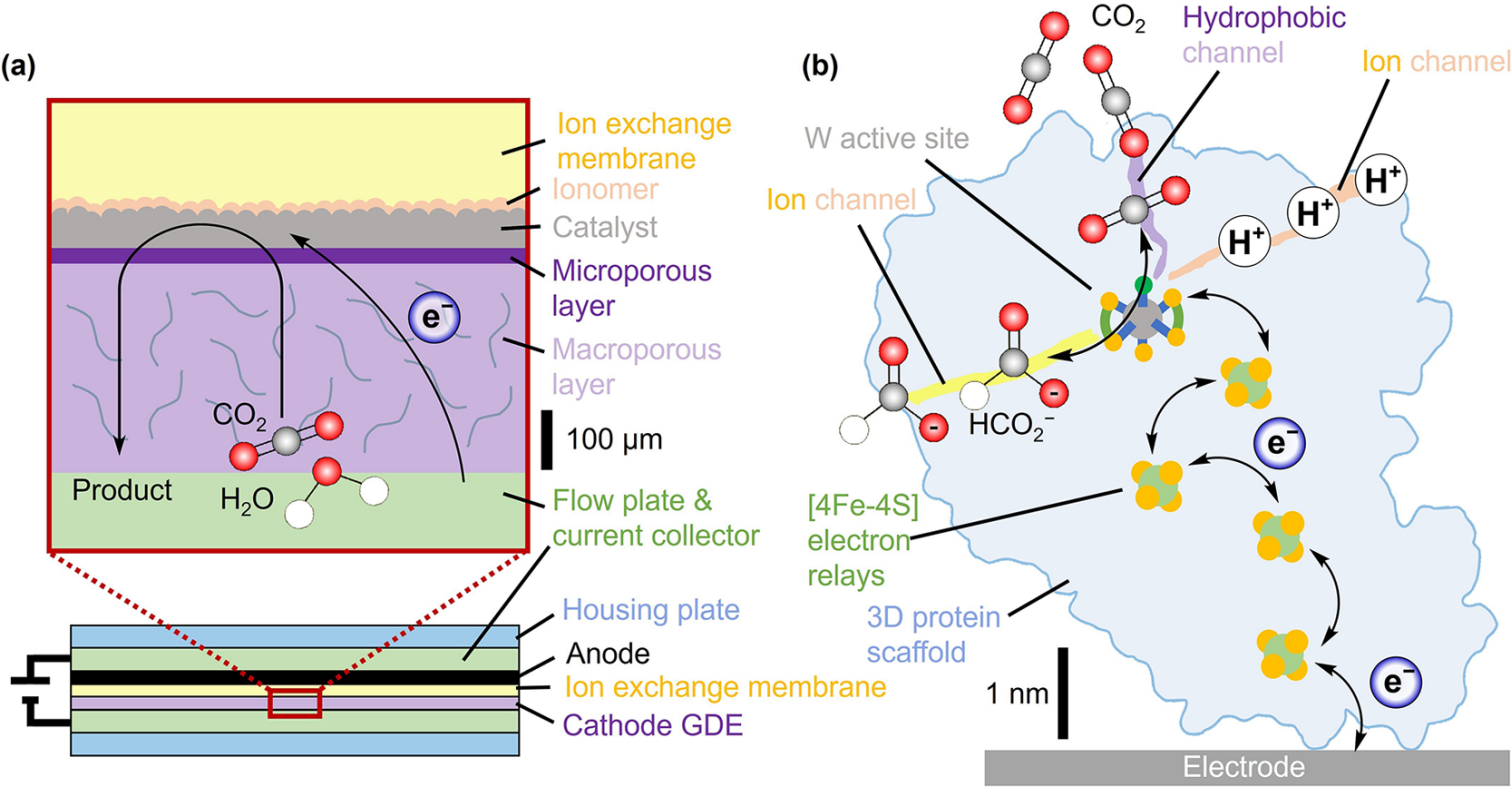
Analogous comparison between (a) the schematic of a CO_2_ electrolyzer with the identified key components and (b) the schematic of W-Fdh immobilized on an electrode, emphasizing the substrate, product and ion channels as well as electron transport relays. The parallel roles are highlighted by the same color coding (e.g., the gray catalyst in (a) is analogous to the gray W active site in (b)). In (b), the bicolored ion channel (yellow and salmon) depicts its bifunctional correspondence to the ion exchange membrane (yellow) and ionomer layer (salmon) in (a). Adapted from ref 55. Copyright 2023 the Authors, published by Wiley-VCH Verlag GmbH & Co. KGaA, under CC BY.

This section describes notable works that progressed Fdh systems from fundamental studies to scale-up application, by achieving CO_2_ electrolysis in a two-electrode electrolysis configuration, long-term formate production, or the transition to flow systems.

Leveraging on the reversibility of *Nv*FdhAB and hydrogenase, an *in vitro* enzymatic-metal oxide system was developed to mimic the biological FHL complex (Figure 6, Figure 28a). This semiartificial system which links the two reversible redox enzymes (H_2_ase and Fdh) on a pair of IO-ITO electrodes is capable of performing the interconversion of H^+^/CO_2_ and H_2_/formate.^151^ By wiring the enzymes in a two-electrode configuration in the presence of all substrates and products, a marginal positive or negative voltage was sufficient to drive the reaction in either direction, demonstrating the reversible unbiased electrocatalysis. Upon applying a voltage of 0.2 V (in the presence of formate) or −0.2 V (in the presence of H_2_), H_2_ or formate could be generated with a FE of 79% and 81%, respectively. This reversibility and interconversion were also achieved with Fdh and H_2_ase coassembled on ITO nanoparticles without external electrochemical wiring. This concept of a semiartificial FHL system that couples the two half-reactions provides a roadmap for on demand H_2_ storage and release.

**Figure 28:**
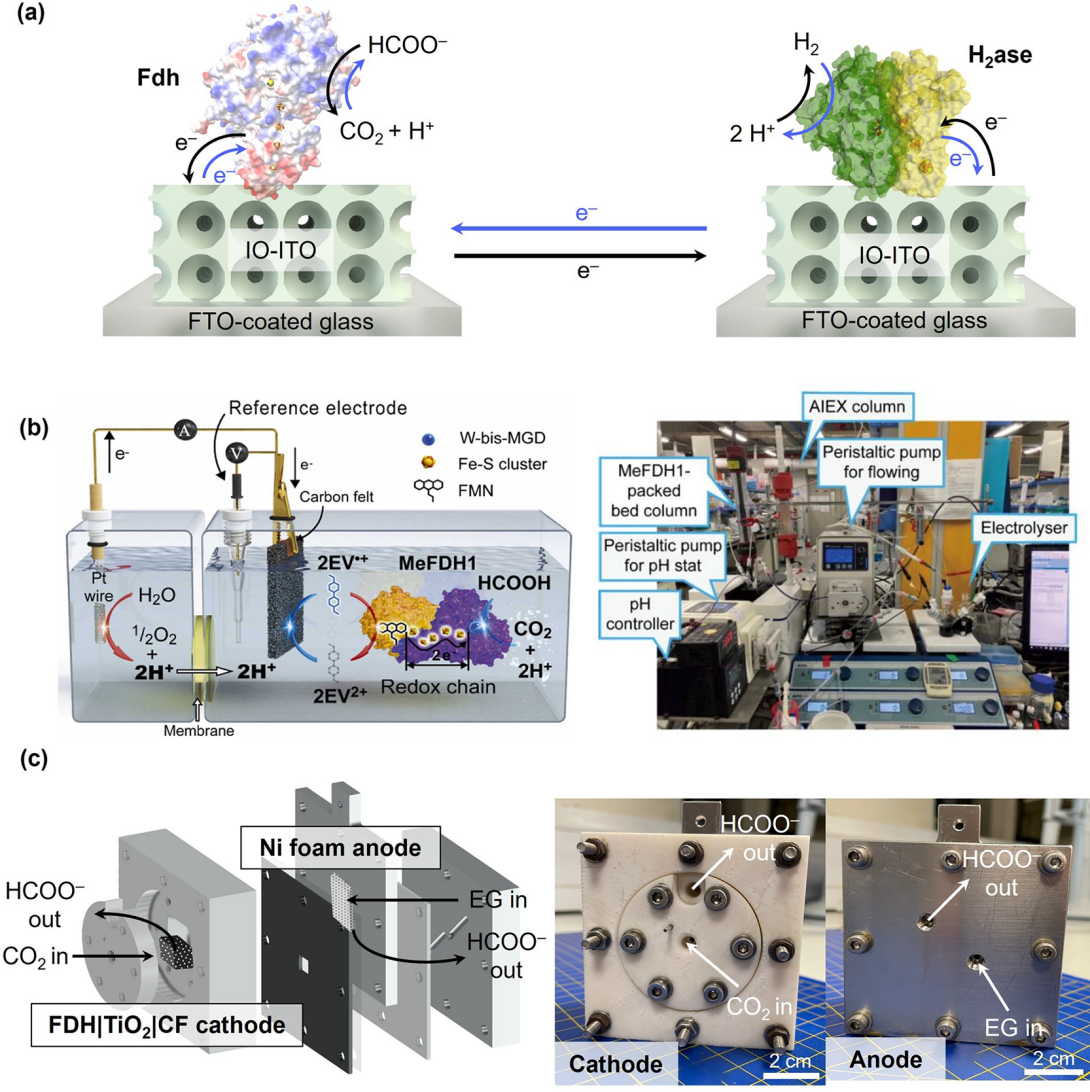
(a) Representation of semiartificial FHL complex mimic consisting of Fdh (PDB: 6SDV)^51^ and hydrogenase (H_2_ase, PDB: 5JSK)^264^ immobilized on IO-ITO electrodes for the reversible interconversion of formate to H_2_ and CO_2_.^151^ (b) Schematic of the mediated-enzymatic CO_2_ reduction to HCOOH by MeFdh1 as well as a photographic image of the overall flow system featuring the MeFdh1-immobilized packed bed column and anion-exchange resin (AIEX) column for *in situ* formate separation. Reproduced from ref 453 with permission. Copyright 2024 the Authors, published by Elsevier Ltd., under CC BY. (c) Schematic and images of an enzymatic flow electrolyzer highlighting the cathode consisting of Fdh immobilized onto TiO_2_ coated carbon felt (Fdh|TiO_2_|CF) and the Ni foam anode for paired CO_2_ and ethylene glycol (EG) electrolysis to formate. Adapted from ref 343. Copyright 2025 the Authors, published by Wiley-VCH Verlag GmbH & Co. KGaA, under CC BY.

Expanding upon the developments in electrode engineering to optimize the performance of CO_2_ reduction by Fdh, recent works have started to explore systems where Fdh can be employed for long-term operation with the aim of continuous formate production. One example is the MET-based flow reactor system established using Fdh1 from *M. extorquens* AM1 (*Me*Fdh1). Reduced ethyl viologen (EV^·+^) was continuously supplied as the electron mediator through electrochemical regeneration and flowed to MeFdh1 immobilized on Ni^2+^–nitrilotriacetic acid agarose (Figure 28b).^453^ As compared to using unbound MeFdh1, the immobilization of MeFdh1 alleviates the denaturation of enzymes by the shear stress of CO_2_ bubbling. The resulting MeFdh1-packed plug flow reactor obtained an optimal formate production at near-unity FE for over 200 h, with a final formate concentration of >1.7 M. Despite the remarkable performance obtained, the use of freely diffusing mediators can result in crossovers and back reactions which may present issues in the development of paired electrolysis devices (i.e., CO_2_ reduction with an anodic reaction in 2-electrode configuration).

Similarly, in earlier work,^454^ the developed gas-diffusion-type biocathode modified with [W]-Fdh from *M. extorquens* AM1 attained a high current density toward CO_2_ reduction but required freely diffusible viologen-based mediators, rendering the cathode challenging for device fabrication.

To circumvent the use of freely diffusing redox mediators, a redox-polymer/enzyme-modified gas diffusion electrode was fabricated.^335^ The constructed polymer matrix not only allows large amount of enzymes to be loaded but also effectively wired to accept electrons from the carbon cloth electrode substrate. Furthermore, the gas diffusion layers enable the use of gaseous CO_2_ as substrate, hence eliminating mass transport limitations. Despite attaining a stable current for 45 h, FE of formate was far less (∼3.7%) than expected and and only the qualitative formation of formate was reliably observed.

While the above-mentioned works have demonstrated how Fdh can be utilized for long-term operation or integrated in gas-diffusion-electrodes, the scope of the applications is still restricted to half-cell electrolysis reaction in in three-electrode configurations. To address this limitation, a DET-based flow enzymatic electrolyzer was developed, which pairs CO_2_ reduction by Fdh to waste (plastic and biomass) oxidation for paired formate production (Figure 28c).^343^ Utilizing a cathode composed of *Nv*FdhAB-immobilized on TiO_2_-sintered carbon felt and a commercial nickel foam as anode, the device completely eliminates the use of mediators and achieves near-unity FE toward formate for both half reactions (i.e., cell FE of ∼200%). Leveraging on the low overpotential requirement of *Nv*FdhAB, the enzymatic electrolyzer could be operated at low full-cell voltage (−1.5 V) to produce formate continuously for over 122 h.

The deployment of Fdh in these flow or device systems has marked significant progress from the characterization of Fdh-electrode interface to the application of these enzymes for fuel production. This extends the role of Fdh from a model electrocatalyst that paves the design of synthetic CO_2_ reduction electrocatalysts, that may become an electrocatalyst that can be integrated for CO_2_ electrolysis for long-term fuel production.

## 7 Fdh Photoelectrochemistry

This section will focus on the use of Fdhs as model cocatalysts for photoelectrochemical CO_2_ reduction to formate. This will cover immobilized and homogeneous Fdh on various photoelectrodes for light-driven CO_2_ fixation.

### 7.1 Overall Design of a Photoelectrochemical Cell

A photoelectrochemical (PEC) cell directly couples light absorption with chemical transformations, enabling solar-driven energy conversion and storage.^455–462^ Its core components typically include a semiconducting photoelectrode that harvests photons, a cocatalyst to facilitate the desired interfacial catalytic redox reaction, and an electrolyte containing the relevant substrates. To investigate fundamental processes, a conventional three-electrode configuration is often employed, comprising the illuminated photoelectrode as the working electrode, a reference electrode to control the potential, and a dark counter electrode to complete the circuit. This arrangement is particularly suited for probing half-reactions at either a photoanode or a photocathode under controlled bias conditions. For the demonstration of overall solar-to-chemical conversion, particularly under bias-free operation, more integrated device architectures and components are required. In such cases, Fdh-based photoelectrochemistry can be implemented in a conventional two-compartment cell using different designs. These include: (i) a single light absorber system employing either a photocathode or a photoanode, (ii) a dual light absorber configuration, in which a photoanode and photocathode are coupled in a tandem arrangement to complement light absorption, and (iii) an integrated semiartificial leaf, which is a fully assembled wireless standalone device. Despite significant advancements in synthetic cocatalysts for electrochemical CO_2_ reduction, their application in PEC systems is often limited due to potential-dependent selectivity, high overpotentials, high cost, and fabrication challenges (e.g., high temperatures).^463–467^ In contrast, the biocatalyst Fdh offers several advantages, including near-zero overpotential, exceptional selectivity with near unity FE independent of potential, simple processability (e.g., drop-casting), and optimal performance under mild conditions. These attributes make Fdh an ideal model cocatalyst for CO_2_ reduction in photoelectrochemical devices.

### 7.2 Redox Mediated-Photoelectrochemistry

The first photoelectrochemical CO_2_ fixation with Fdh was reported in 1984, using a p-type indium phosphide (p-InP) photocathode in the presence of a MV redox mediator (Figure 29a, Table 5).^468^ Thanks to the suitable conduction band position and excellent semiconducting properties of p-InP, a photocurrent density of 9 mA cm^–2^ was achieved at −0.06 vs SHE under 50–60 mW cm^–2^ illumination from a 150 W tungsten-halogen lamp under ambient conditions. This setup produced 0.6 mM formate with a FE of 80–93%. The use of 23 nmol Fdh led to a high TON of 21000. In 2016, integration of *Thiobacillus* sp. Fdh (*Ts*Fdh) with a hydrogen-terminated silicon nanowire photocathode for solar-driven formate synthesis from CO_2_ and water was demonstrated.^469^ The photocathode efficiently delivered hydrides to an Rh-based organometallic electron mediator, enabling regeneration of the NADH cofactor that activates *Ts*Fdh, achieving a FE of 16% at an applied bias of 1.8 V. By further coupling the photocathode with a cobalt phosphate-coated triple-junction silicon photoanode, solar-to-chemical conversion under natural sunlight using a solar-tracking system was demonstrated. However, like other MET systems employing soluble redox mediators, this configuration faced challenges in product separation and the toxicity of MV. To this end, a compactly integrated bioelectrode was developed in 2016 for light-driven CO_2_ reduction.^470^ In this system, Fdh from *Candida boidinii* and NADH were coimmobilized within a polydopamine thin film on a GCE. The immobilized NADH cofactor acted as an electron mediator, addressing issues of MV toxicity and product separation. Pairing this biocathode with a CoP_i_|BiVO_4_ photoanode achieved unassisted solar formate production with simultaneous OER, maintaining a stable unbiased photocurrent density >0.1 μA cm^–2^ for 24 h. This system generated 16 μM formate with a near unity FE of 99.18 ± 6.77% in a two-electrode configuration.

**Figure 29:**
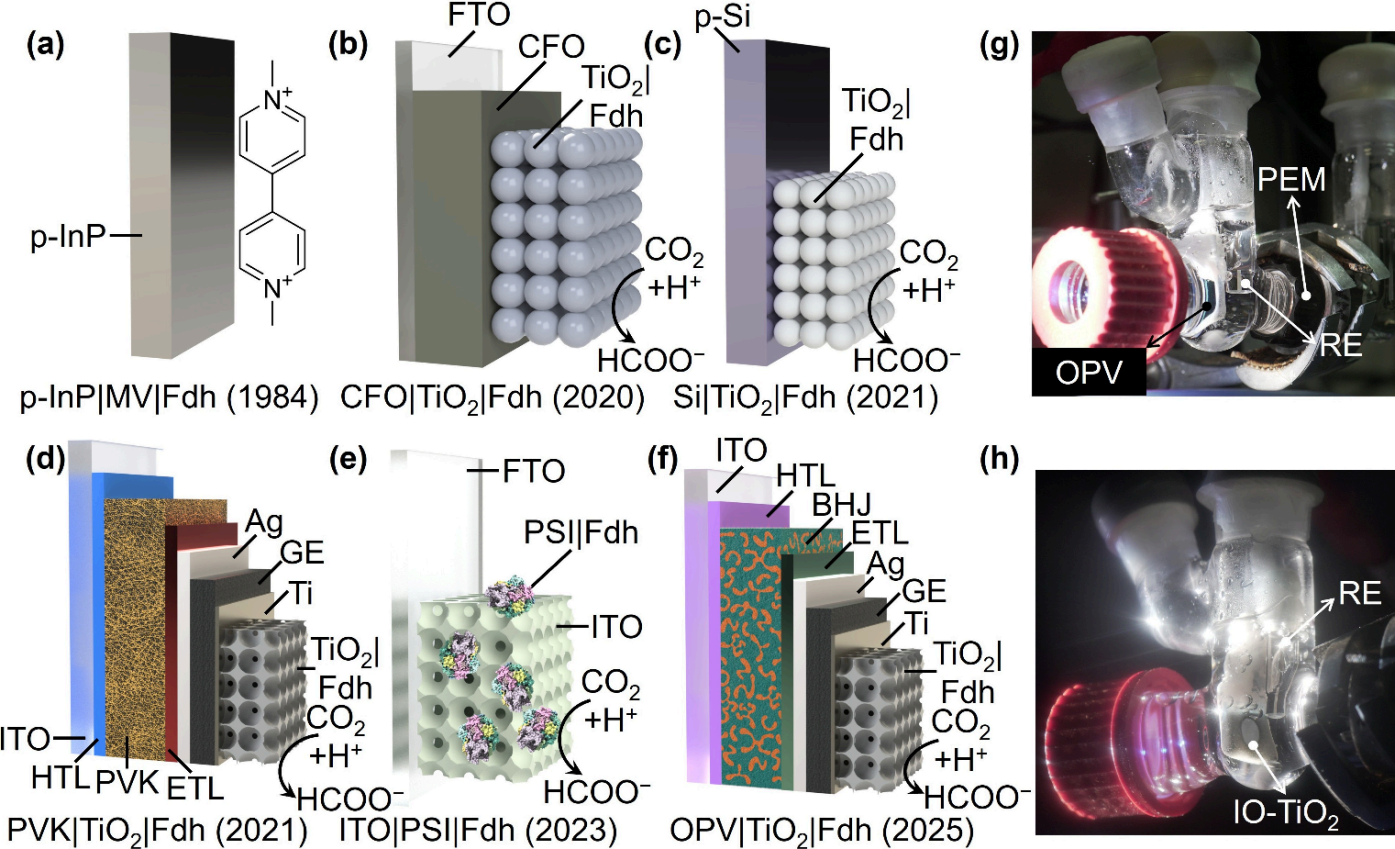
Development of Fdh-based semiartificial photocathodes for light-driven CO_2_ fixation with focus on DET to Fdh: (a) p-InP|MV (1984),^468^ (b) CFO|TiO_2_ (2020),^478^ (c) Si|TiO_2_ (2021),^479^ (d) PVK|TiO_2_ (2021),^337,475^ (e) ITO|PSI (PDB: 1JB0,[Bibr ref480] 2023),^471^ (f) OPV|TiO_2_ (2025).^342,353,365^ The first Fdh-based photoelectrochemistry using p-InP|MV for MET is listed for comparison. (g) Front and (h) back photographic images of (f) an OPV|TiO_2_ photocathode with immobilized Fdh under operation. Reproduced from ref 342. Copyright 2025 the Authors, published by Elsevier Inc., under CC BY. Numerical data can be found in Table 5.

**Table 5:** Summary of Fdh-Based Photocathodes for CO_2_ Reduction to Formate

Year	Photocathode	Organism	Enzyme (loading)	Onset (V_RHE_)	–J (mA cm^–2^) at E (V_RHE_)	Stability (h) at E (V_RHE_) (post J%)	FE (%)	Electrolyte	Irradiance (mW cm^–2^)	Wavelength (nm)	ref
1984	p-InP|MV	*Pseudomonas oxalaticus*	Fdh (23 nmol)	0.7	0.6 at 0.45	2 at 0.45 (N/A)	80–93	Phosphate (0.5 M), NaHCO_3_ (0.5 M), MV (2 mM), pH 6.8	50–60	<900	^468^
2020	FTO|CFO|TiO_2_	*Clostridium ljungdahlii*	Fdh (1 U)	0.9	1.2 at 0.2	6 at 0.39 (N/A)	13.5	Phosphate (0.1 M), HCO_3_^‑^ (50 mM), pH 6.5	100	>420	^478^
2021	p-Si|TiO_2_	*Nv*H	FdhAB (160 pmol)	N/A	0.07 at – 0.1	12 at −0.1 (67%)	99 ± 12	KHCO_3_ (0.5 M), pH 7.3	100	>400	^479^
2021	PVK|TiO_2_	*Nv*H	FdhAB (104 pmol)	1.1	3.7 at 0.35	10 at 0.4 (55%)	80 ± 10	MOPS (0.1 M), CsCl (50 mM), NaHCO_3_ (1 mM), pH 4.6	100	AM1.5G	^337^
2023	ITO|PSI	*Methylobacterium extorquens*	AM1Fdh (704 pmol)	0.65	0.007 at 0.35	22 at 0.35 (10%)	15	KHCO_3_ (50 mM)	100	400–800	^471^
2025	OPV|TiO_2_	*Nv*H	FdhAB (100 pmol)	1.0	5.7 at −0.1	10 at 0.6 (52%)	98 ± 2	NaHCO_3_ (50 mM), KCl (50 mM), pH 6.45	100	AM1.5G	^342^
2025	OPV|TiO_2_	*Nv*H	FdhAB (125 pmol)	1.0	6 at 0	12 at 0.8 (80%)	54	MOPS (0.1 M), NAD^+^ (1 mM), acetophenone (50 mM), pH 6	100	AM1.5G	^353^
2025	OPV|TiO_2_	*Nv*H	FdhAB (500 pmol)	1.0	10 at 0	10 at 0.6 (73%)	90 ± 6	NaHCO_3_ (50 mM), KCl (50 mM), pH 6.45	100	AM1.5G	^365^

### 7.3 Establishing DET in PEC

Drawing upon the advances in interfacial and materials engineering for Fdh electrochemistry, including strategies such as electrostatic interactions, chemisorption, and covalent binding, substantial efforts have been devoted to establishing DET between photoelectrodes and Fdh to enable the assembly of bias-free solar devices. To this end, the predominant approach has involved the utilization of porous materials, which provide an enlarged surface area for enzyme immobilization and catalysis to produce considerable amount of formate. In 2018, a notable advancement involved the development of a PEC tandem device, pairing Photosystem II immobilized on a dpp (phosphonated diketopyrrolopyrroles) dyesensitized IO-TiO_2_ photoanode with a *Nv*FdhAB-immobilized IO-TiO_2_ dark biocathode for CO_2_ photoreduction paired with water oxidation to O_2_ (Figure 30a, Table 6).^325^ This device required only a small applied bias of 0.3 V to drive the overall reaction. Under visible-light irradiation for 1 h, a half-lifetime of approximately 8 min was observed, attributed to the photodegradation of Photosystem II. Despite this limitation in stability, the system achieved formate production of 0.185 ± 0.017 μmol cm^–2^ with a FE of 70 ± 6%. In addition to photosystem II, the use of photosystem I was reported as a biological light absorber to activate Fdh from *Methylobacterium extorquens*.^471^ In this system, photosystem I and Fdh were sequentially assembled on an IO-ITO electrode (Figure 29e), enabling direct transfer of photogenerated electrons to Fdh for CO_2_-to-formate conversion. Remarkably, the setup achieved an operational stability of 22 h and with a FE of ∼15%.

**Figure 30:**
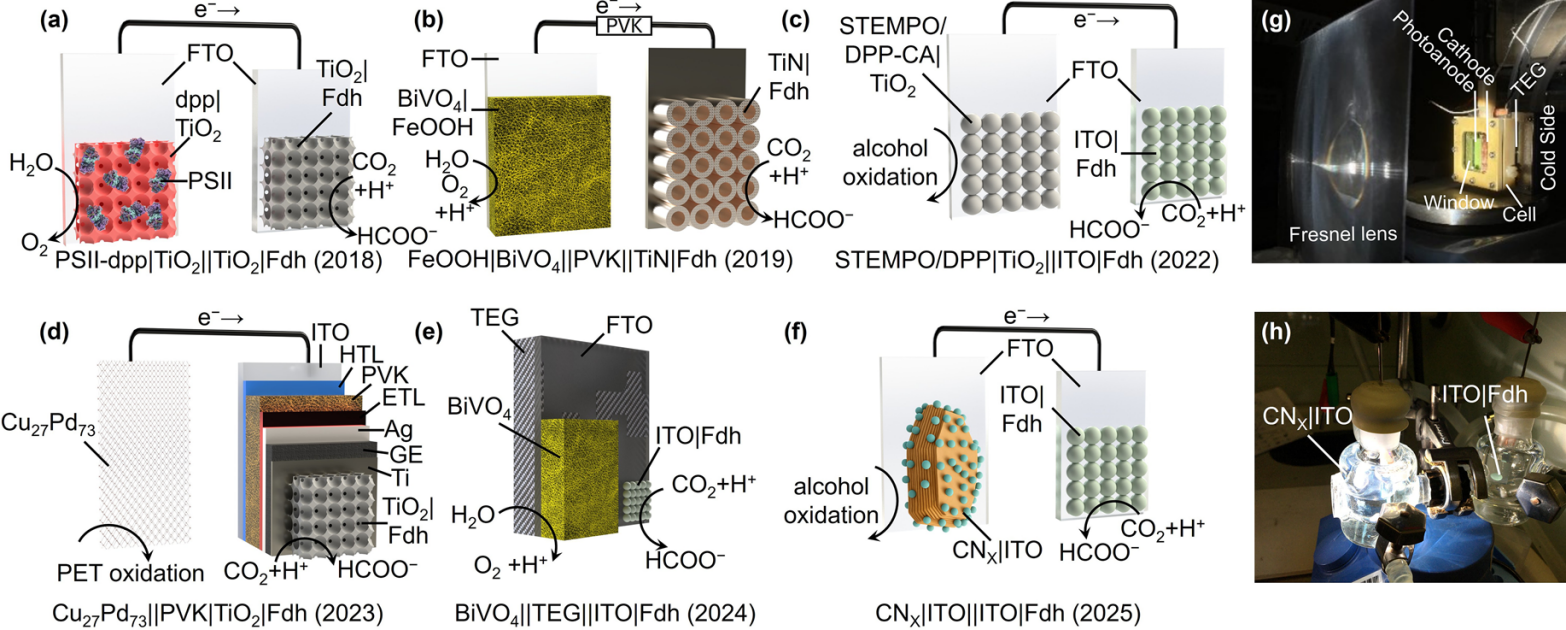
Development of Fdh-based semiartificial tandem devices featuring single dark electrode for light-driven CO_2_ fixation: (a) PSII-dpp|TiO_2_||TiO_2_ (PDB: 3WU2,[Bibr ref472] 2018),^325^ (b) TiN||BiVO_4_|FeOOH||PVK (2019),^473^ (c) STEMPO/DPP-CA|TiO_2_||ITO (2022),^474^ (d) TiO_2_|PVK||Cu_27_Pd_73_ (2023),^475^ (e) ITO||BiVO_4_||TEG (2024),^476^ (f) ITO||CN_X_–ITO (2025).^477^ (g) Photo of (e) the BiVO_4_–TEG device. Reproduced from ref 476. Copyright 2024 the Authors, published by Elsevier Inc., under CC BY. (h) Photo of (f) the 3 configurations of CN_X_–ITO devices. Reproduced from ref 477. Copyright 2025 the Authors, published by American Chemical Society, under CC BY. Numerical data can be found in Table 6.

**Table 6:** Summary of Fdh-Based PEC Tandem Devices Featuring Single Light Absorbers for Unbiased Reactions

Year	Photocathode||Anode	Organism	Enzyme (loading)	Onset (V)	–J at 0 V (mA cm^–2^)	Stability (h) at 0 V (post J%)	FE (%)	STF (%)	Electrolyte	Irradiance (mW cm^–2^)	Wavelength (nm)	ref
2023	TiO_2_|PVK||Cu_27_Pd_73_	*Nv*H	FdhAB (100 pmol)	–0.6	1	10 (95%)	96 ± 4	N/A	MOPS (0.5 M), pH 6.4	100	AM1.5G	^475^

Year	Cathode||Photoanode||Booster	Organism	Enzyme (loading)	Onset (V)	–J at 0 V (mA cm^–2^)	Stability (h) at 0 V (post J%)	FE (%)	STF (%)	Electrolyte	Irradiance (mW cm^–2^)	Wavelength (nm)	ref
2018	TiO_2_||TiO_2_|dpp-PSII	*Nv*H	FdhAB (34 pmol)	0	0.092 (0.3 V)	1 (0.3 V, 8%)	70 ± 6	N/A	NaHCO_3_ (0.1 M), KCl (50 mM), pH 6.5	100	AM1.5G	^325^
2019	TiN||BiVO_4_|FeOOH||PVK	*Clostridium ljungdahlii*	Fdh (0.5 U)	N/A	0.12	8 (79%)	83.1	0.08	NaPi (0.1 M), HCO_3_^–^ (50 mM), pH 6.5	100	>420	^473^
2022	ITO||TiO_2_| STEMPO/DPP-CA	*Nv*H	FdhAB (52 pmol)	–0.2	0.03	6 (99%)	74 ± 17	N/A	NaHCO_3_ (50 mM), pH 6.4	100	>420	^474^
2024	ITO||BiVO_4_||TEG	*Nv*H	FdhAB (400 pmol)	N/A	0.08	10 (21%)	97 ± 5	0.032	NaHCO_3_ (67 mM), pH 7	300	AM1.5G	^476^
2025	ITO||CN_X_–ITO	*Nv*H	FdhAB (100 pmol)	–0.2	0.075	10 (67%)	94 ± 3	N/A	NaHCO_3_ (0.1 mM), KCl (50 mM), pH 6.7	100	AM1.5G	^477^

A dark biocathode was developed in 2019 using a 3D titanium nitride (TiN) nanoshell electrode, which offers attractive conductivity, porosity, and stability (Figure 30b).^473^ When *Cl*Fdh was immobilized, a high current density of nearly 1 mA cm^–2^ with a FE of 94% was achieved. This biocathode was integrated into a PEC tandem device comprising a perovskite photovoltaic and a FeOOH|BiVO_4_ photoanode, enabling unassisted solar formate production at an average rate of 0.78 μmol h^–1^ with a FE of 77%. However, incorporating an additional photovoltaic element into a metal oxide photoanode markedly increases the overall system complexity, thereby underscoring the need for an integrated PEC cell design. To address this limitation, an integrated photocathode was fabricated by loading 160 pmol Fdh onto mesoporous TiO_2_-coated silicon (Figure 29c), delivering a photocurrent density of 0.07 mA cm^–2^ and a FE of 99 ± 12% over 12 h.^479^

The first Fdh-based PEC tandem cell featuring dual light absorbers was developed in 2020, consisting of a CuFeO_2_|CuO|TiO_2_|*Cl*Fdh photocathode (Figure 29b) and a FeOOH|BiVO_4_ photoanode (Figure 31a, Table 7).^478^ In a two-electrode configuration, this bioelectrocatalytic cell reduced CO_2_ to formate using water as the electron donor, delivering an unbiased photocurrent density of 0.15 mA cm^–2^ with no significant degradation for 12 h. The formate production rate was 0.10 μmol h^–1^ cm^–2^, with a FE of 34% and a solar-to-formate conversion efficiency (STF) of 0.01%. Additionally, a monolithic FeOOH|BiVO_4_||CFO|TiO_2_|Fdh device was fabricated, representing a wireless semiartificial leaf (Figure 31a). Under simulated AM 1.5G illumination, it operated continuously for 24 h, producing 3.1 μmol of formate with an O_2_ evolution rate of 1.57 μmol h^–1^ cm^–2^. However, the limited Fdh loading was identified as one of the performance bottlenecks, which could be addressed by employing hierarchically structured scaffolds.

**Table 7:** Summary of Fdh-Based PEC Tandem Featuring Dual Light Absorbers for Unbiased Reactions

Year	Photocathode||Photoanode	Organism	Enzyme (loading)	Onset (V)	–J at 0 V (mA cm^–2^)	Stability (h) at 0 V (post J%)	FE (%)	STF (%)	Electrolyte	Irradiance (mW cm^–2^)	Wavelength (nm)	ref
2020	TiO_2_|CFO||BiVO_4_|FeOOH	*Clostridium ljungdahlii*	Fdh (1 U)	N/A	0.15	12 (87%)	33.5	0.008	Phosphate (0.1 M), HCO_3_^–^ (50 mM), pH 6.5	100	>420	^478^
2021	TiO_2_|PVK||BiVO_4_|TiCo	*Nv*H	FdhAB (104 pmol)	–0.5	0.8	10 (75%)	83 ± 5	0.8	MOPS (86 mM), NaHCO_3_ (50 mM), CsCl (50 mM), pH 6.4	100	AM1.5G	^337^
2025	TiO_2_|OPV||BiVO_4_|TiCo	*Nv*H	FdhAB (500 pmol)	–0.8	0.55	10 (73%)	98 ± 8	0.6 ± 0.1	NaHCO_3_ (50 mM), KCl (50 mM), pH 6.45	100	AM1.5G	^342^
2025	TiO_2_|OPV||Fe_2_O_3_|Ni(OH)_*x*_	*Nv*H	FdhAB (500 pmol)	–0.3	1.1	10 (62%)	97	N/A	NaHCO_3_ (50 mM), KCl (50 mM), pH 6.45	100	AM1.5G	^365^

**Figure 31:**
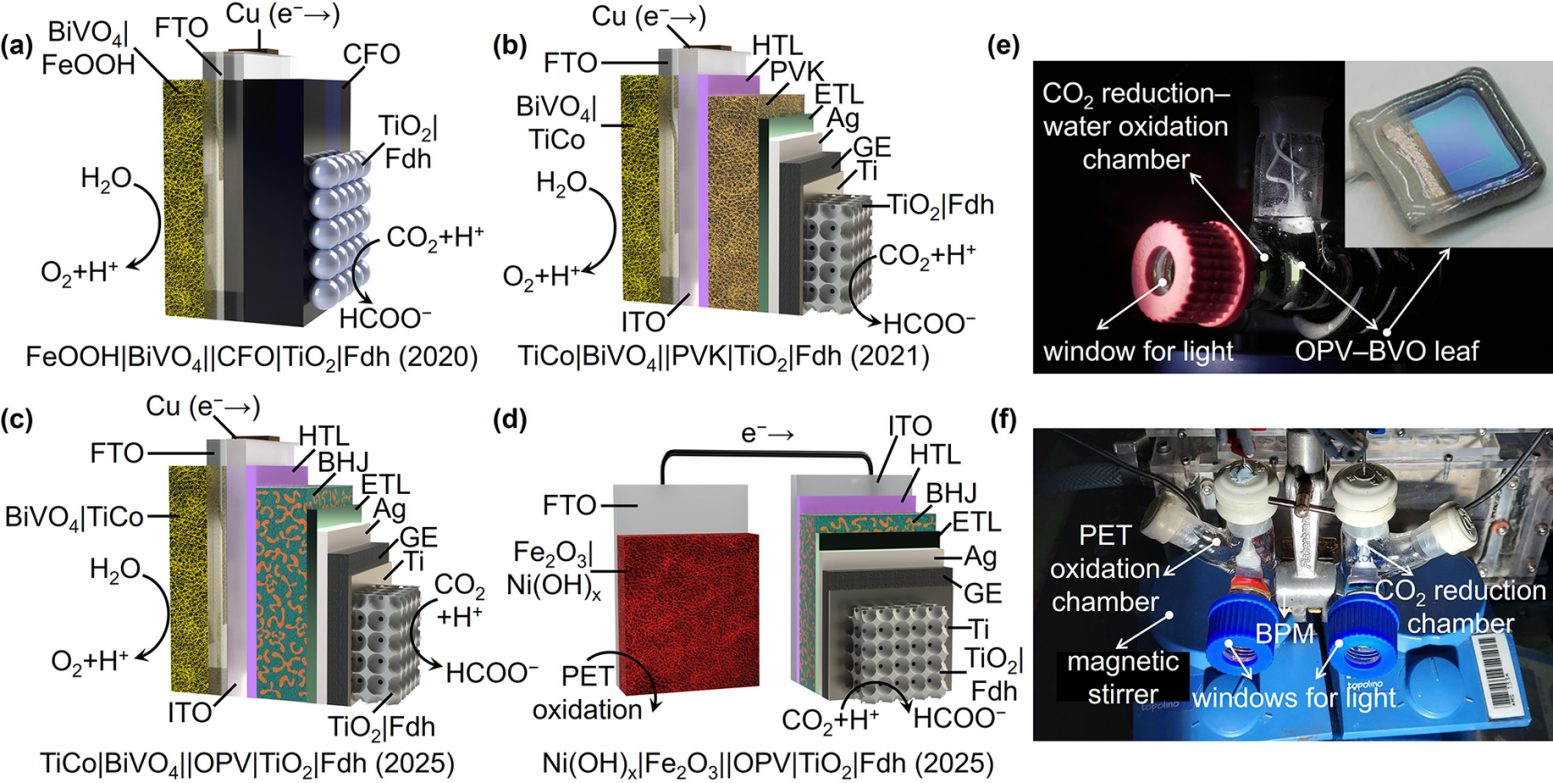
Development of Fdh-based semiartificial tandem devices featuring dual light absorbers for light-driven CO_2_ fixation: (a) FeOOH|BiVO_4_||CFO|TiO_2_ (2020),^478^ (b) TiCo|BiVO_4_||PVK|TiO_2_ (2021),^337^ (c) TiO_2_|OPV||Fe_2_O_3_|Ni­(OH)_*x*_ (2025),^365^ (d) TiO_2_|OPV||BiVO_4_|TiCo (2025).^342^ (e) Photo of (c) the OPV–BVO tandem. Adapted from ref 342. Copyright 2025 the Authors, published by Elsevier Inc., under CC BY. (f) Photo of (d) the OPV–hematite tandem. Reproduced from ref 365. Copyright 2025 the Authors, published by the Royal Society of Chemistry, under CC BY. Numerical data can be found in Table 7.

Combining semiconducting photoelectrodes with Fdhs immobilized on hierarchically structured IO-TiO_2_ scaffolds represents a state-of-the-art DET configuration that balances high enzyme loading with efficient charge transfer. For example, a STF efficiency of 0.8% was achieved using a combination of an encapsulated perovskite photocathode (Figure 29d) and a BiVO_4_ photoanode (Figure 31b).^337^ In a three-electrode configuration, the perovskite photocathode was functionalized with a thick layer of IO-TiO_2_ scaffold to host *Nv*FdhAB, resulting in a high photocurrent density of 4 mA cm^–2^ at 0.4 V vs RHE. The unassisted TiCo|BiVO_4_|perovskite|IO-TiO_2_|Fdh semiartificial leaf produced 70 ± 20 mmol cm^–2^ of formate with a FE of 83 ± 5% and a TOF of 4 s^–1^ during 10 h of operation, using only sunlight, CO_2_, and water as inputs (Figure 31b). The large photovoltage generated by the perovskite|IO-TiO_2_|Fdh photocathode enables the construction of an unassisted PEC tandem cell when paired with a Cu_27_Pd_73_ dark anode (Figure 30d).^475^ Under a two-electrode configuration, the system exhibited an onset potential of – 0.6 V and a photocurrent density of 1 mA cm^–2^ at 0 V applied bias to facilitate CO_2_ reduction coupled with the simultaneous oxidative valorization of pretreated PET plastic, maintaining stable operation for 10 h.

The IO-TiO_2_|Fdh scaffold has also been integrated with organic photovoltaic (OPV) photocathodes in bias-free devices (Figure 29f–h).^365^ To avoid the use of noninnocent additives such as Good’s buffers, 100 pmol CA was coimmobilized with 500 pmol Fdh to mitigate the steep pH gradient generated during CO_2_ conversion and thereby stabilize the local environment. This system delivered a photocurrent density approaching 10 mA cm^–2^ with near-unity selectivity for formate production in a three-electrode configuration (Figure 31c,e). Furthermore, coupling the semiartificial OPV photocathode with a nanostructured hematite photoanode modified with a Ni-based catalyst enabled unbiased PEC comproportionation of CO_2_ and pretreated PET plastic into formate over 10 h (Figure 31d,f).

In addition to biological and semiconducting light absorbers, co-catalysts and dye molecules such as STEMPO, DPP, and RuP have been integrated into mesoporous TiO_2_ to construct dye-sensitized photoanodes (Figure 30a,c).^474^ When coupled with an Fdh-loaded mesoporous ITO dark cathode, this system enabled unbiased solar CO_2_ reduction to formate coupled with selective alcohol oxidation, achieving a bias-free photocurrent density of up to 30 μA cm^–2^ over 6 h and a TON_Fdh_ of 4,532 ± 945. The coupling of CO_2_ reduction with selective alcohol oxidation has recently been extended to a carbon nitride (CN_X_)|ITO photoanode.^477^ When paired with an Fdh|mesoITO dark cathode, the system achieved a bias-free photocurrent density of 65 ± 15 μA cm^–2^ over 10 h, producing 10.6 ± 0.1 μmol cm^–2^ of formate with a FE of 94 ± 3% (Figure 30f,h).

Recently, a thermoelectric generator (TEG) was paired with a BiVO_4_ photoanode integrated with a *Nv*FdhAB loaded mesoITO biocathode (Figure 30e,g), aiming to harness the full solar spectrum and manage waste heat.^476^ The BiVO_4_ photoanode absorbed UV–visible light while the TEG utilized the IR range, enabling complementary light management. This integrated device achieved a photocurrent density up to 0.47 ± 0.05 mA cm^–2^ for CO_2_ photoreduction, producing 42 ± 8 mmol of formate after 10 h of continuous operation.

With the aim of extending semiartificial CO_2_ fixation on Fdh beyond formate, OPV|IO-TiO_2_|Fdh devices have been coupled with both biological and synthetic catalysts to enable CO_2_-mediated enantioselective organic synthesis, as well as using an engineered *E. coli* for biomass production (Figure 32). A triple-enzyme cascade on an ITO-coated carbon felt electrode was demonstrated, where formate produced from CO_2_ reduction by *Nv*FdhAB was directly utilized by *Candida boidinii* Fdh for NADH regeneration.^353^ The regenerated cofactor was then used by an alcohol dehydrogenase to reduce acetophenone to chiral 1-phenylethanol, achieving an enantiomeric excess of 93% and a conversion yield of 38%.

In a complementary approach, renewable formate generated from Fdh-catalyzed CO_2_ reduction was used to drive the asymmetric hydrogenation of acetophenone with a synthetic Noyori-Ikariya catalyst, obtaining a high yield of 78% and excellent enantioselectivity of 94% for (R)-1-phenylethanol.^342^ A semiartificial leaf with an engineered *E. coli* has been established for solar-driven CO_2_-to-biomass conversion, where adapted *E. coli* was shown to efficiently utilize formate as an energy source for growth, demonstrating electrode–microbe compatibility and biomass production. This integrated two-stage design mimics natural photosynthesis, with abiotic light-driven formate generation and biotic carbon fixation, presenting a formate bioeconomy approach for coupling renewable energy with microbial production.^481^ These examples illustrate the potential of integrating Fdh-based CO_2_ reduction with downstream synthetic, enzymatic, and microbial transformations to produce high-value, complex chemicals in a sustainable and selective manner.

**Figure 32:**
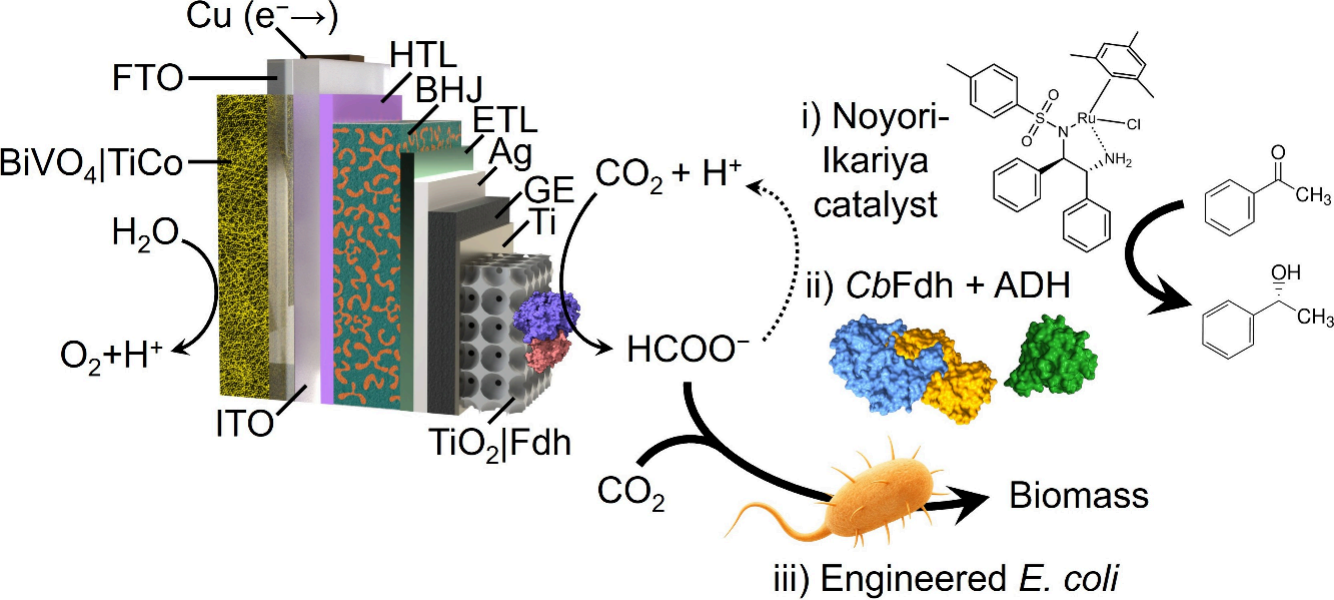
Semiartificial cascade for CO_2_-mediated enantioselective organic synthesis of chiral 1-phenylethanol from acetophenone using either (i) a synthetic Noyori-Ikariya catalyst^342^ or (ii) biological catalysts containing *Candida boidinii* fdh (PDB: 5DN9)^117^ and alcohol dehydrogenase (PDB: 1ZK4).^353^ CO_2_ photoreduction was achieved using *Nv*FdhAB (PDB: 6SDV).^51^ (iii) Semiartificial photosynthesis of biomass using formate from CO_2_ reduction and an engineered *E. coli*.^481^

Four decades after the first MV-mediated semiartificial CO_2_ fixation using Fdh, research has clearly shifted toward DET. This strategy offers distinct advantages in device integration and minimizing enzyme demand. While early DET systems delivered photocurrent densities below 1 mA cm^–2^, within just five years the state-of-the-art DET photocathodes now achieve ∼10 mA cm^–2^ with nearly 100% FE, requiring 2 orders of magnitude less Fdh than MET-based photocathodes.

## 8 Fdh Photochemistry

This section will focus on the use of Fdh as a model cocatalyst for photochemical CO_2_ reduction to formate. This will cover Fdh in solution, suspension, and sheets for CO_2_ reduction, coupling Fdh with organic and inorganic light absorbers. This will also highlight strategies for binding Fdh with light absorbers.

### 8.1 Light Absorbers

Integrating Fdh with photocatalysts represents an innovative semiartificial approach that combines the high selectivity of enzymes with energy-efficient, light-driven reactions.^65,66,68^ These biohybrid assemblies show significant potential for harnessing sunlight to convert CO_2_ into formate under mild conditions, using simple components and device configuration. A typical semiartificial photocatalytic system consists of a light absorber, biocatalyst, substrate, buffer solution, and electron donor. Upon light irradiation, light absorbers such as semiconductors or dyes convert absorbed photons into photoexcited electrons that are localized in the conduction band or LUMO, respectively.^486–488^ The energy levels of conduction band or LUMO must be higher than the redox potential of the target reaction, such as the interconversion between CO_2_ and formate, to thermodynamically allow electron transfer and enable the reaction bidirectionally with the Fdh (Figure 33). Substrates like CO_2_ or formate are introduced into the solution by purging or dissolving prior to photocatalysis. Electron donors are usually sacrificial reagents, though replacing them with value-added reactions (e.g., waste valorization) or oxygen evolution reaction is highly desirable.^489–492^ The pH of the solution is maintained in the neutral range (pH 6–8) using the Good’s buffer or an inorganic buffer to ensure high enzyme activity.^357^ Recent studies have revealed that Good’s buffer can also act as an electron donor in photocatalytic reactions, thereby complicating the oxidation pathway.^342^ To address this issue, efforts have been directed toward employing CA and/or inorganic buffers to enable cleaner solar-driven chemistry (see Section 5.2).

**Figure 33:**
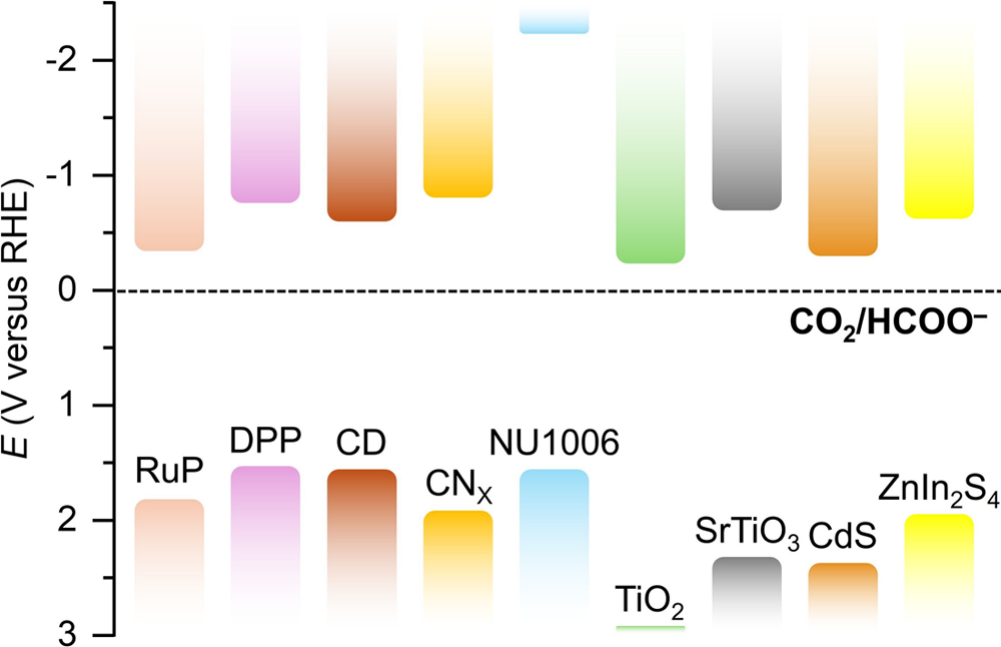
Energy band positions of light absorbers for construing a Fdh biohybrid. Data source: RuP,^493^ DPP,^493^ carbon dot (CD),^338^ CN_X_,^494,495^ NU1006,^496^ TiO_2_,^497^ SrTiO_3_,^373^ CdS,^498^ ZnIn_2_S_4_.^483^ The redox potential for CO_2_/HCOO^–^ catalyzed by Fdh is displayed as reference.^61^ The energy band positions and redox potential are converted to RHE.

### 8.2 Photocatalysis Using Electron Mediators

Photochemical activation of Fdh via DET has been an important challenge in the field, early studies have predominantly focused on developing mediated photocatalytic systems that employ soluble redox mediators, e.g. using Fdh from *Saccharomyces cerevisiae*.^499–502^ In 2002, photocatalytic CO_2_-to-formate conversion using this Fdh was reported in a MV-mediated system with zinc tetrakis­(4-methylpyridyl) porphyrin (ZnTMPyP) as the light absorber and triethanolamine (TEOA) as the sacrificial electron donor.^499^ This system produced 0.1 mM formate after 4 h of visible-light irradiation, corresponding to a 10.4% yield for CO_2_ to formate conversion. The same system has been further optimized and characterized in 2004,^500^ producing 62 μM after 3 h in the presence of 30 μM MV. In 2006, a similar MV-mediated system was constructed using chlorophyll-a as a photosensitizer and NADPH as an electron donor, generating 0.056 mM formate after 4 h of irradiation.^501^ To replace the NADH cofactor, a series of artificial cofactors were synthesized in 2017 to evaluate how ionic groups in viologen derivatives influence Fdh catalytic activity.^502^ Among them, the reduced form 1,1′-diaminoethyl-4,4′-bipyridinium salt exhibited the highest catalytic efficiency, reaching 0.25 μM^–1^ min^–1^.

In 2012, a rhodium complex was employed to mediate electron transfer from a graphene-based photocatalyst for the photochemical regeneration of NADH from NAD^+^.^503^ Using TEOA as a sacrificial electron donor, visible-light-driven CO_2_ reduction produced 111 μmol of formic acid within 2 h. Thus, recent efforts to eliminate soluble mediators have led to the development of an integrated mediated system in 2020.^496^ In this approach, the electron mediator Cp*Rh­(2,2′-bipyridyl-5,5′-dicarboxylic acid)Cl was anchored onto a metal–organic framework (MOF) NU-1006, with Fdh immobilized within the MOF pores. This semiartificial system achieved a turnover frequency of 865 h^–1^ over 24 h in the presence of NADH as an electron donor. However, the use of diffusional electron mediators is costly due to the increased system complexity, and they cannot be easily separated from the formate product in the liquid form. Additionally, the most widely used MV compounds are toxic, presenting significant drawbacks for practical applications.

### 8.3 Interfacial Engineering for Direct Electron Transfer

Establishing DET between the light absorber and Fdh presents a promising component and energy efficient strategy for photocatalytic CO_2_ reduction to formate due to system simplicity. The first such configuration was reported in 2019, employing a ruthenium tris-2,2’-bipyridine dye-sensitized TiO_2_ colloidal system (Figure 34a, Table 8).^324^ This approach benefited from the unique advantages of metal oxides, for example, low cost, scalability, and the ability to bind enzymes in their electroactive orientations.^347^ Comprehensive QCM and ATR-IR studies demonstrated the strong and stable binding affinity between *Nv*H Fdh and TiO_2_. As a result, a benchmark TOF of 11 ± 1 s^–1^ was achieved using TEOA as a sacrificial electron donor, without the need for an electron mediator using Ru-dye sensitized TiO_2_. This work shows the importance of metal oxide as a scaffold in effectively immobilizing enzymes and establishing efficient DET with directly wired Fdh, paving the way for developing more controlled immobilization of enzymes and more efficient semiartificial photosynthetic systems. The active TiO_2_|Fdh interface enabled the development of floating semiconductor–enzyme photoreforming catalysts (Figure 34b).^504^ By immobilizing TiO_2_ onto silica-based hollow glass microspheres, the resulting floating photocatalyst allows vertical solar illumination and facilitates product separation. This composite supports simultaneous solar-driven CO_2_ reduction coupled to pretreated cellulose oxidation for 24 h, producing up to 1.16 ± 0.04 mmol g^–1^ of formate as the sole product through a comproportionation reaction.

**Table 8:** Summary of Fdh-Based Photocatalytic CO_2_ Reduction Systems

Year	Photocatalysis	Organism	Enzyme (loading)	TOF (h^–1^)	Stability (h)	Electrolyte	Irradiance (mW cm^–2^)	Wavelength (nm)	ref
2019	RuP|TiO_2_	*Nv*H	FdhAB (12 pmol)	40,000	24	NaHCO_3_ (0.1 M), TEOA (0.1 M), pH 6.5	100	AM1.5G (UV-filtered)	^324^
2019	DPP|TiO_2_	*Nv*H	FdhAB (12 pmol)	18,000	24	NaHCO_3_ (0.1 M), TEOA (0.1 M), pH 6.5	100	AM1.5G (UV-filtered)	^324^
2022	*a*-CD	*Nv*H	FdhAB (40 pmol)	2,100	24	NaHCO_3_ (0.1 M), DTT (10 mM), pH 6.7	100	AM1.5G	^338^
2024	BiVO_4_-SrTiO_3_	*Nv*H	FdhAB (50 pmol)	1,274	10	NaHCO_3_ (0.1 M), Co(bpy)_3_ (0.5 mM), pH 6.7	100	AM1.5G	^373^
2025	Ru-micelle	*Nv*H	FdhAB (20 pmol)	333	24	NaHCO_3_ (0.1 M), NaHAsc (0.1 M), pH 6.7	100	AM1.5G	^482^
2025	ZnIn_2_S_4_	*Nv*H	FdhAB (50 pmol)	375	8	NaHCO_3_ (0.1 M), PP-ol (1 mg/mL), pH 6.7	100	AM1.5G	^483^
2025	CN_*X*_–ITO	*Nv*H	FdhAB (40 pmol)	35,000	10	NaHCO_3_ (0.1 M), KCl (50 mM), MBA (7.5 mM), pH 6.7	100	AM1.5G	^477^
2026	CPE-FBI hydrogel	*Nv*H	FdhAB (50 pmol)	2,000	48	NaHCO_3_ (0.1 M), NaHAsc (0.1 M), pH 6.7	100	AM1.5G	^484^
2026	pBP-DB+	*Nv*H	FdhAB (50 pmol)	27,488	48	NaHCO_3_ (0.1 M), MBA (50 mM), pH 6.7	100	AM1.5G	^485^

**Figure 34:**
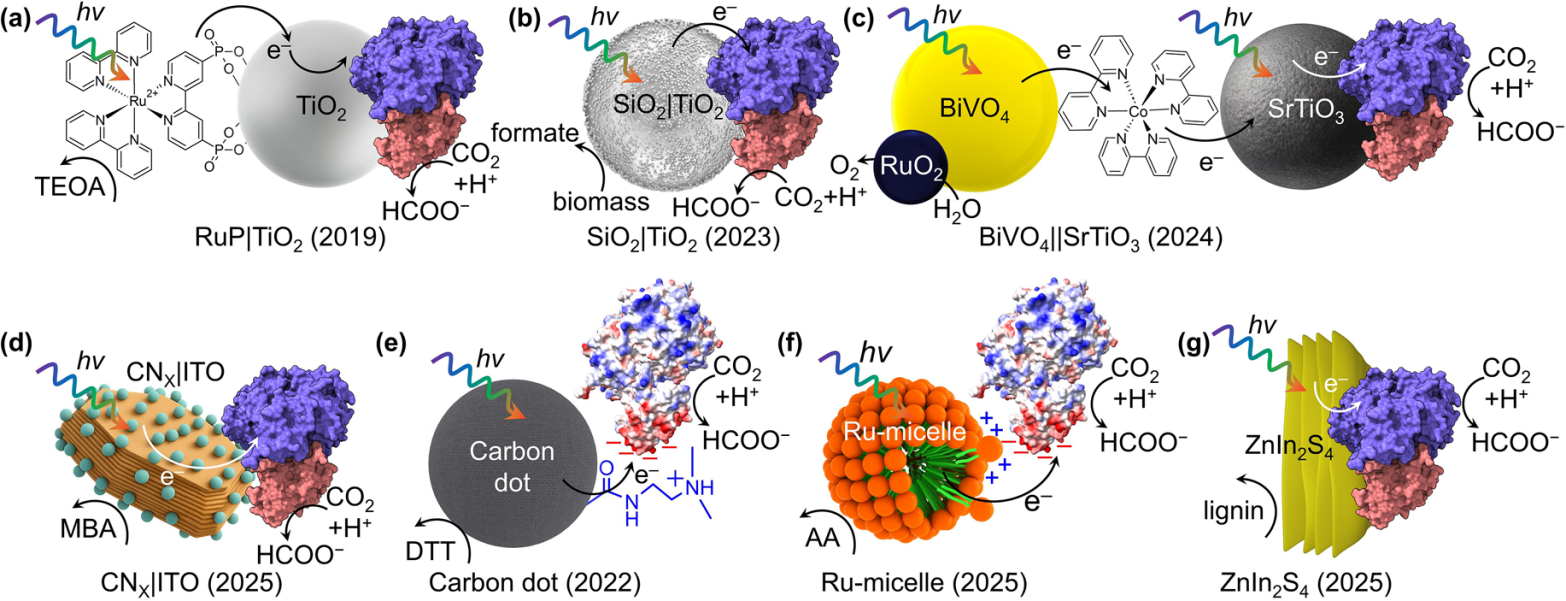
Interfacing Fdhs (PDB: 6SDV)^51^ with light absorbers for semiartificial photocatalysis. Representative metal oxides systems: (a) Ru|TiO_2_ (2019),^324^ (b) floating SiO_2_|TiO_2_ (2023),^504^ (c) Z-scheme BiVO_4_||SrTiO_3_ (2024),^373^ (d) CN_X_|ITO (2025).^477^ Representative electrostatic association systems: (e) carbon dot (2022),^338^ (f) Ru-micelle (2025).^482^ Representative metal sulfide system: (g) ZnIn_2_S_4_ (2025).^483^ The electron donors are triethanolamine (TEOA), 4-methylbenzyl alcohol (MBA), dithiothreitol (DTT), and ascorbate acid (AA). Numerical data can be found in Table 8.

Inspired by the metal oxide–Fdh interactions, the first colloidal Z-scheme system for semiartificial photosynthesis was developed by combining SrTiO_3_:La,Rh, BiVO_4_:Mo, and a [Co­(bpy)_3_]­SO_4_ complex (Figure 34c).^373^ The spatial separation of oxidation and reduction semiconductors enabled the use of water as an electron donor, achieving overall water splitting and eliminating the need for a sacrificial electron donor. The assembled Fdh|SrTiO_3_:La,Rh|[Co­(bpy)_3_]^3+/2+^|BiVO_4_:Mo|RuO_2_ system continuously generated formate, while simultaneously producing molecular oxygen with the input of only sunlight, water, and carbon dioxide. This system yielded 319 ± 27 μmol formate g^–1^ with a TON of 12740 over 10 h. QCM analysis revealed that the strong immobilization of Fdh on SrTiO_3_:La,Rh was key to enabling efficient and stable DET.

Beyond adopting the semiconducting properties of metal oxides, conductive oxides also serve as efficient electron relay materials, enabling enzymes to be loaded in their electroactive orientations. In this context, ITO was employed to bridge CN_X_ and Fdh (Figure 34d),^477^ facilitating the construction of colloidal photocatalysts, photosheets, and PEC devices.^477^ Among the tested oxides such as SiO_2_, TiO_2_, and ZrO_2_, ITO proved to be the most effective oxide support, with an optimal composition of 75% ITO in CN_X_. When 40 pmol Fdh was immobilized onto 4 mg of the CN_X_–ITO composite to form a colloidal photocatalyst, a formate areal activity of 0.70 ± 0.06 μmol cm^–2^ was achieved over 10 h.

Besides metal oxides, the emerging metal sulfide semiconductor ZnIn_2_S_4_ (ZIS) has demonstrated the ability to directly transfer photogenerated electrons to Fdh for solar-driven CO_2_ reduction (Figure 34g),^483^ achieving a formate TON of 2,200 after 4 h, corresponding to an overall quantum yield of 0.1%. This reductive process was coupled with the selective oxidation of a lignin model compound PP-ol to PP-one, achieving a high conversion yield of approximately 80% after 24 h. The interactions between ZIS and Fdh were further investigated using QCM and PEIS. Fdh adsorption on a ZIS-coated quartz chip occurred rapidly, reaching saturation within 6 min at a surface coverage of 6.8 pmol cm^–2^. The binding was also strong, as evidenced by only 18% Fdh desorption during subsequent buffer washing process. Equivalent circuit fitting of the Nyquist plots revealed a decrease in *R*_*ct*_ from 160 kΩ to 76 kΩ, confirming the catalytic contribution of Fdh for CO_2_ reduction.

Given that the protein surrounding the distal Fe–S cluster of Fdh consists of negatively charged amino acids, such as aspartic and glutamic acids, functionalizing a light absorber with positively charged terminals is a promising strategy for establishing DET via electrostatic interactions. This strategy enables the mimicry of the positively charged natural redox partner cytochrome *c*_3_ through the use of functionalized light absorbers. The first mediator-free, homogeneous photocatalytic system for CO_2_ reduction to formate using Fdh was developed on amorphous carbon dots (a-CDs, Figure 34e).^338^ By chemically modifying a-CDs with positively charged amine groups, efficient DET was achieved with a TOF of 3,500 h^–1^. The electrostatic binding was systematically characterized using QCM, ATR-IR, and electrochemical techniques, highlighting the critical role of surface charge in semiartificial photosynthesis. Additionally, the rational selection of a sacrificial electron donor is crucial for optimizing performance. For instance, the use of ethylenediaminetetraacetic acid (EDTA), a commonly used sacrificial electron donor, can shield the surface charge of a-CDs thereby disrupting electrostatic interactions.^338^ In contrast, the use of neutral DTT doubled the photocatalytic performance compared to EDTA.

Inspired by the natural enzyme-membrane interface, a biohybrid assembly was developed using supramolecular surfactants as light absorbers for CO_2_-to-formate conversion.^482^ The Ru-based photosensitizer self-assembled into micellar structures, with positively charged Ru head groups that electrostatically associated with the protein surface near the distal Fe–S cluster of Fdh (Figure 34f) thereby facilitating efficient DET in the presence of sodium ascorbate as the sacrificial electron donor. This biomimetic enzyme–micelle assembly achieved a TON of 8,000 over 24 h. The strong association between the Ru micelles and Fdh was confirmed by nanosecond time-resolved absorption and emission spectroscopy, which indicated structural changes in the micellar assemblies.

Similar electrostatic strategies have been used in interfacing *Nv*FdhAB with positively charged organic semiconductors such as CPE-FBI hydrogel and pBP-DB+ photosheet,^484,485^ achieving a TON (48 h) of 2,000 and 27,488, respectively. Time-resolved absorption spectroscopy revealed the reaction kinetics of the biohybrid assemblies.

## 9 Scale-Up Applications of Fdhs

Challenging the common assumption that enzymes are fragile, limited in stability for real-world practicality, this section highlights the scale-up potential of metal-dependent Fdh and their proof-of-principle-use in prototype devices.

*Nv*FdhAB was integrated with a CN_*x*_–ITO composite photocatalyst sheet of 50 cm^2^ geometrical surface area for CO_2_ reduction to formate coupled with 4-methylbenzyl alcohol oxidation to the corresponding aldehyde (Figure 30f).^477^ The CN_*x*_–ITO|Fdh photocatalyst sheet was contained in an airtight and transparent photoreactor and submerged in CO_2_-saturated solution, enabling outdoor operation under natural sunlight (Figure 35a). This work highlighted the facile fabrication of such photocatalyst sheets and demonstrated the high areal and specific activity toward formate and aldehyde production. The construction of this semiartificial photoreactor device and its operation for over 3 days showcases the possibility for deploying enzymes under real-world conditions for solar fuel production.

**Figure 35:**
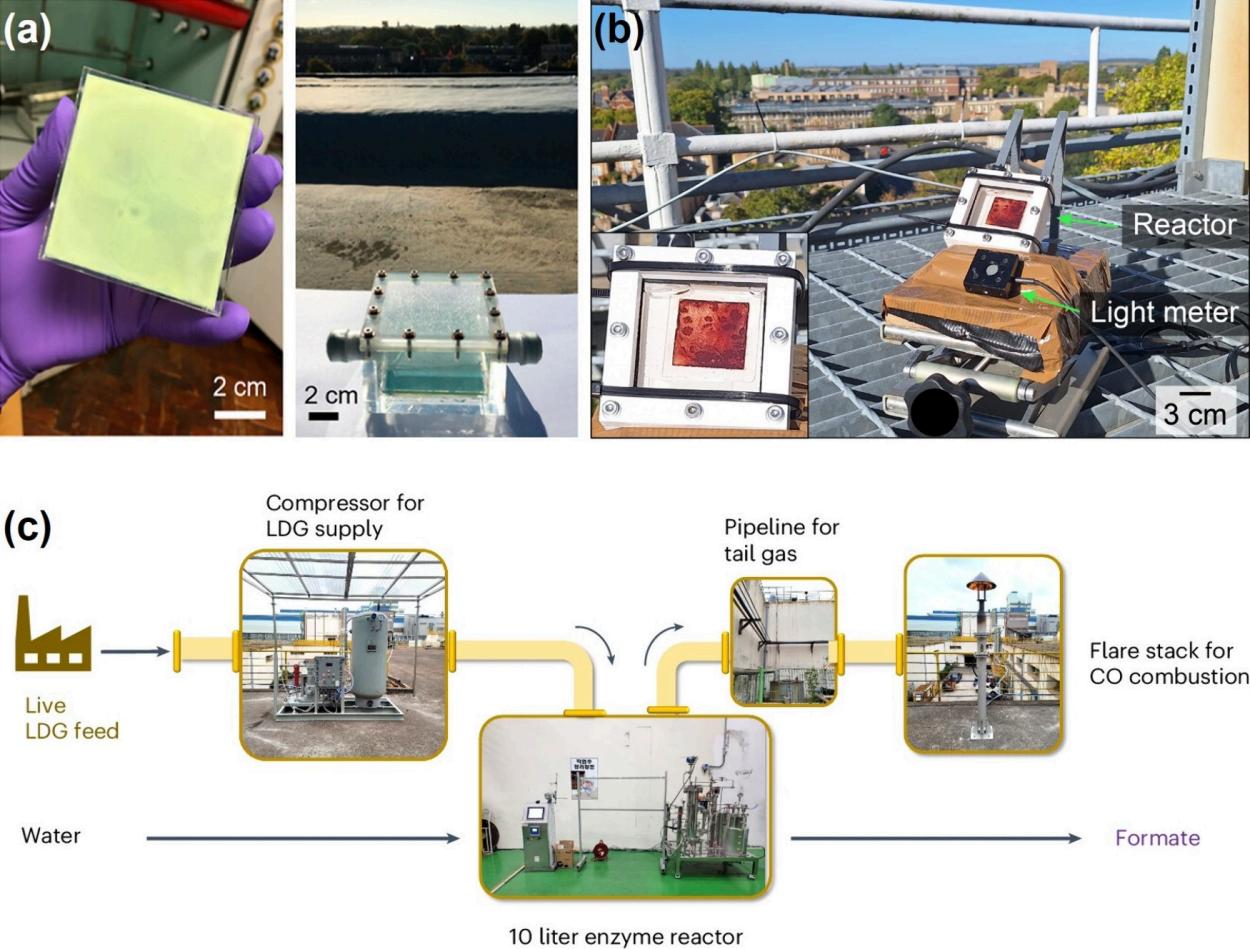
(a) Images depicting the 50 cm^2^ CNx–ITO|Fdh photocatalyst sheet (deposited on FTO substrate) which was housed in a Perspex (poly­(methyl methacrylate)) photoreactor for outdoor experiments under natural sunlight. Reproduced from ref 477. Copyright 2025 the Authors, published by American Chemical Society, under CC BY. (b) Images of COF|CN_*x*_|Fdh photopanel architecture and the outdoor experiment utilizing an individual 3 × 3 cm^2^ photopanel in a 3D-printed reactor. Photographs taken by Dr Suvendu Karak.^505^ (c) Schematic of the scaled-up enCOH using the 10-L enzyme-immobilized reactor with a continuous feed of LDG for formate production. Reproduced from ref 17. Copyright 2024 the Authors and Springer Nature Limited, under CC BY.

An azo-functionalized β-ketoenamine-based covalent organic framework (COF) integrated with functionalized CN_*x*_ in a composite (COF|CN_*x*_) provides a versatile photosheet scaffold.^505^ The system integrates *Nv*FdhAB for CO_2_-to-formate generation coupling with the oxidative valorization of ethylene glycol derived from polyethylene terephthalate (PET) waste. The use of spatially defined, pixelated photopanels enables independent tuning of enzymatic reactions, thereby overcoming intrinsic kinetic mismatches between biocatalysts and allowing control over product distributions. These systems have been validated under real-world conditions, including rooftop operation using scalable photopanels (active area of 9 cm^2^) for the selective generation of formate and glycolaldehyde.^505^ The combination of modularity, scalability and compatibility with waste-derived feedstocks highlights the potential of such platforms for decentralized solar-driven refineries.^506^ Biocompatible and metal-free photosensitizers such as conjugated polyelectrolytes have also been developed as photocatalytic biohybrid sheets through self-assembly with NvFdhAB, which achieved a TOF of h^–1^ during the stable operation over 48 h.^485^

Apart from solar devices, the *Me*Fdh1 enzyme was employed in tandem with a CO dehydrogenase (*Ch*COdh2) for enzymatic CO hydration (enCOH) in a 10-L-scale reactor, converting industrial gas emissions into formate (Figure 35c).^17^ The enCOH involved the conversion of CO into CO_2_ by *Ch*COdh2 and subsequently into formate by *Me*Fdh1, mediated by the EV_ox_/EV_red_ redox partner. Notably, the scaled up 10-l reactor immobilized with both enzymes was operated at mild conditions (room temperature and neutral pH) and could utilize a live feed of Linz–Donawitz gas (LDG) directly without pretreatment. This work hence featured the real-world applicability of Fdh and presented enCOH as a promising avenue for valorizing industrial flue gas.

## 10 Concluding Remarks and Future Perspectives

The growing urgency to mitigate anthropogenic CO_2_ emissions and develop sustainable energy solutions has driven extensive research into catalytic CO_2_ fixation. Metal-dependent Fdhs, particularly those containing Mo or W cofactors, have emerged as promising CO_2_ reduction model catalysts for sustainable formate production. The direct integration of Fdhs into semiartificial photosynthetic platforms provides a versatile and inspirational framework for harnessing renewable electrons for CO_2_ conversion. Such biohybrid systems not only offer a pathway to sustainable chemical synthesis and solar energy conversion, but also exemplify how insights from natural enzymology can inspire the rational design of next-generation synthetic catalysts for CO_2_ fixation.

Despite these advances, several challenges remain in the deployment of Fdh-based systems. The oxygen sensitivity of many metal-dependent Fdhs limits their operational stability, and difficulties and cost in enzyme expression, purification, and functional immobilization continue to hinder scalability. Electron-transfer bottlenecks at the abiotic–biotic interface, kinetic imbalances between CO_2_ reduction and formate oxidation, as well as mass transport limitations further constrain overall performance. Additionally, the full potential of Fdh in semiartificial systems is yet to be realized due to limited understanding of interfacial phenomena, including enzyme orientation, reaction intermediates, and the mechanisms governing charge transfer and recombination. Addressing these fundamental and engineering challenges will be critical for improving efficiency, scalability, and durability in semiartificial CO_2_ fixation systems.

Looking forward, several avenues hold promises for advancing Fdh-based semiartificial CO_2_ fixation. First, protein engineering via directed evolution could generate Fdh variants with enhanced oxygen tolerance, higher stability, and improved CO_2_ reduction activity.^507–509^ Rational modification of the enzyme cofactor may also improve catalytic rates, improve bias favored CO_2_ reduction, and reduce inhibition. Second, advances in materials science, including high-performance light absorbers, combined with interfacial engineering strategies to improve charge transfer and reduce surface recombination, can enable more efficient integration of Fdhs with photoelectrodes,^510–512^ molecular photosensitizers,^513–516^ and nanostructured scaffolds.^517–519^ In particular, the development of hierarchically structured porous supports that maximize enzyme loading at their electroactive orientation is expected to improve overall system performance.^365,520,521^ Third, in situ and operando characterization techniques, including spectroelectrochemistry,^522–525^ infrared,^526–528^ Raman,^529–532^ and X-ray spectroscopy,^533–536^ and advanced optical microscopy,^537–540^ will be instrumental in uncovering the mechanistic details of Fdh–material interactions and the charge carrier dynamics at the abiotic–biotic interface. Such insights can guide the rational design of more robust, efficient, and scalable biohybrid systems.

Reducing the reliance on redox mediators, sacrificial electron donors, and noninnocent buffers in semiartificial systems is a crucial step toward the practical application of Fdh-based biohybrids. This approach not only simplifies system design but also enables cleaner chemical production. Establishing DET between synthetic and Fdh materials eliminates the need for toxic or costly mediators and minimizes photovoltage loss between the mediator and the Fdh active site.^541–543^ It also opens avenues for interfacial engineering through electrostatic association, covalent binding, and nanoconfinement. Although most studies on Fdh photocatalysis have relied on sacrificial electron donors that generate mixed, low-value oxidation products, recent advances have demonstrated the use of photogenerated holes in productive oxidation reactions such as water oxidation,^342,373^ organic transformations,^365,477^ and biomass valorizations.^483,504^ These efforts highlight the potential for fully exploiting photogenerated electron–hole pairs, thereby enhancing solar-to-chemical conversion efficiencies beyond systems limited to reductive processes. Good’s buffers are widely used in biological applications owing to their suitable pH range, high solubility, optical transparency, and inertness to enzymatic reactions.^357–359^ However, these organic zwitterionic compounds are readily oxidized,^339,360,476^ restricting their compatibility with demanding oxidation reactions in photocatalysis and reducing oxidation FEs in photoelectrochemical systems. The search for alternatives to such noninnocent buffers has therefore gained increasing attention, leading to the recent development of semiartificial platforms for CO_2_-mediated asymmetric synthesis.^342^

Expanding the scope of Fdh-based CO_2_ conversion beyond formate through domino chemistry presents exciting opportunities for producing higher-value multicarbon products. Coupling Fdh-catalyzed formate production with downstream microbial, enzymatic, or chemical cascades could establish integrated or consecutive CO_2_ valorization pathways, bridging solar energy conversion with sustainable chemical synthesis.^342,345,353,481,544^ Integration with solar-driven or electrochemical systems also enables on-demand and decentralized CO_2_ fixation. Future efforts can be put into optimizing enzymatic activity while maintaining long-term operational stability and enabling large-scale, continuous formate production in flow systems for downstream applications. Furthermore, the real-world applicability of Fdh can be further established with the use of industrial flue gas, i.e., improving the performance of Fdh under low CO_2_ concentrations present with trace amounts of O_2_.

Thus, semiartificial photosynthesis using metal-dependent Fdhs represents a unique and powerful approach for sustainable CO_2_ fixation. While challenges remain, advances in protein/materials engineering and operando characterization provide clear pathways for overcoming these barriers. Continued interdisciplinary research will be essential for realizing the full potential of Fdh-based semiartificial CO_2_ fixation. These efforts hold promise of transforming CO_2_ from a persistent greenhouse gas into a sustainable carbon feedstock for energy storage and chemical synthesis, contributing both to climate mitigation and the development of a circular economy.
